# Prediction of Verbal Abilities From Brain Connectivity Data Across the Lifespan Using a Machine Learning Approach

**DOI:** 10.1002/hbm.70191

**Published:** 2025-03-25

**Authors:** Deborah Früh, Camilla Mendl‐Heinisch, Nora Bittner, Susanne Weis, Svenja Caspers

**Affiliations:** ^1^ Institute of Neuroscience and Medicine (INM‐1) Research Centre Jülich Jülich Germany; ^2^ Institute for Anatomy I, Medical Faculty & University Hospital Düsseldorf Heinrich Heine University Düsseldorf Düsseldorf Germany; ^3^ Institute of Neuroscience and Medicine, Brain and Behaviour (INM‐7) Research Centre Jülich Jülich Germany; ^4^ Institute of Systems Neuroscience, Medical Faculty & University Hospital Düsseldorf Heinrich Heine University Düsseldorf Düsseldorf Germany

**Keywords:** connectivity, language functions, life span, ML analyses, prediction

## Abstract

Compared to nonverbal cognition such as executive or memory functions, language‐related cognition generally appears to remain more stable until later in life. Nevertheless, different language‐related processes, for example, verbal fluency versus vocabulary knowledge, appear to show different trajectories across the life span. One potential explanation for differences in verbal functions may be alterations in the functional and structural network architecture of different large‐scale brain networks. For example, differences in verbal abilities have been linked to the communication within and between the frontoparietal (FPN) and default mode network (DMN). It, however, remains open whether brain connectivity within these networks may be informative for language performance at the individual level across the life span. Further information in this regard may be highly desirable as verbal abilities allow us to participate in daily activities, are associated with quality of life, and may be considered in preventive and interventional setups to foster cognitive health across the life span. So far, mixed prediction results based on resting‐state functional connectivity (FC) and structural connectivity (SC) data have been reported for language abilities across different samples, age groups, and machine‐learning (ML) approaches. Therefore, the current study set out to investigate the predictability of verbal fluency and vocabulary knowledge based on brain connectivity data in the DMN, FPN, and the whole brain using an ML approach in a lifespan sample (*N* = 717; age range: 18–85) from the 1000BRAINS study. Prediction performance was, thereby, systematically compared across (i) verbal [verbal fluency and vocabulary knowledge] and nonverbal abilities [processing speed and visual working memory], (ii) modalities [FC and SC data], (iii) feature sets [DMN, FPN, DMN‐FPN, and whole brain], and (iv) samples [total, younger, and older aged group]. Results from the current study showed that verbal abilities could not be reliably predicted from FC and SC data across feature sets and samples. Thereby, no predictability differences emerged between verbal fluency and vocabulary knowledge across input modalities, feature sets, and samples. In contrast to verbal functions, nonverbal abilities could be moderately predicted from connectivity data, particularly SC, in the total and younger age group. Satisfactory prediction performance for nonverbal cognitive functions based on currently chosen connectivity data was, however, not encountered in the older age group. Current results, hence, emphasized that verbal functions may be more difficult to predict from brain connectivity data in domain‐general cognitive networks and the whole brain compared to nonverbal abilities, particularly executive functions, across the life span. Thus, it appears warranted to more closely investigate differences in predictability between different cognitive functions and age groups.

## Graphical abstract

Functional and structural connectivity data was used to predict verbal fluency and vocabulary knowledge in a lifespan sample using two machine learning algorithms. The predictability of verbal compared to nonverbal abilities in younger and older adults was found to be low across whole‐brain and network‐specific functional and structural imaging data.

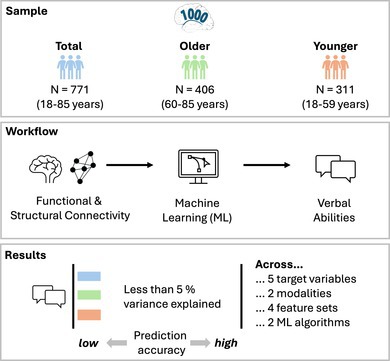

## 1 Introduction

As the aging population continues to grow, research on the age‐related decline of cognitive abilities has become increasingly important. To this end, studies have shown that not all older adults are affected in the same way and that different cognitive domains decline at different rates (Hedden and Gabrieli 2004; Salthouse 2004; Salthouse et al. 2003). While executive functions, for example, processing speed, working memory, and attention, are affected more severely and decline earlier in life, language‐related cognition generally appears to remain more stable until later in life (Hedden and Gabrieli 2004; Salthouse 2004). Nevertheless, it has also been shown that not all language‐related processes may be equally affected by the aging process (Baciu et al. 2016; Gonzalez‐Burgos et al. 2019). While semantic and vocabulary knowledge appear more resistant to decline and may even improve with age, verbal fluency tends to be more vulnerable to the aging process (Gonzalez‐Burgos et al. 2019).

Generally, language functions seem to be grounded in an extended network of frontal, parietal, and temporal regions and to be mainly left lateralized in the great majority of the population (Friederici and Gierhan 2013; Nakajima et al. 2020; Tomasi and Volkow 2020). In this regard, individual areas do not act in isolation, but are embedded in large‐scale brain networks to give rise to complex cognitive abilities through coordinated activity with other brain regions (Binder et al. 1997; Gaudet et al. 2020; Tremblay and Dick 2016; Turken and Dronkers 2011; Turker et al. 2023). In this context, regions are functionally, via functional connectivity (FC), or anatomically, via structural connectivity (SC), linked to other parts of the brain to form brain networks subserving higher cognitive functioning, including language functions.

Beyond the contribution of core language areas, for example, inferior frontal gyrus, posterior temporal cortex, several brain regions have been implicated in support of language functions (Liu et al. 2022; Tremblay and Dick 2016). This extended network seems to additionally comprise, for example, the middle and superior frontal gyri, the insula, the cingulate cortex, and parietal regions (Arrigo et al. 2024), as well as the precuneus (Wagner et al. 2014). Additional activations during verbal tasks, especially in the frontal cortices, have been reported in older adults, with right hemispheric activations possibly related to performance loss (Meinzer et al. 2009, 2012). In contrast, the right fronto‐parietal network (FPN) has specifically been suggested to support executive abilities to compensate successfully for age‐related verbal performance loss (Gonzalez‐Burgos et al. 2019, 2020). Furthermore, engagement of right hemispheric regions related to verbal fluency is not always restricted to older adults but can also be found in younger adults (Arrigo et al. 2024; Martin et al. 2022) and might be more related to performance differences than age itself, hinting at a possible importance of right hemispheric contributions.

To shed further light on this, the involvement of additional, more domain‐general but functionally linked cognitive systems has been discussed (Dick et al. 2010; Liu et al. 2022; Tremblay and Dick 2016). Here, (posterior) parts of the cingulate cortex, the precuneus, and other regions belonging to the Default mode Network (DMN) seem to be particularly supportive (Paschoal et al. 2021). On the one hand, it has been discussed that the DMN enables language comprehension actively by building a cooperative system with core language and executive functions (Liu et al. 2022), as well as verbal fluency by its strong coupling with core language regions during task performance. On the other hand, its anticorrelated or deactivated state during specific cognitive tasks might facilitate successful language processing (Anticevic et al. 2012).

Furthermore, a recent cross‐sectional study (Stumme et al. 2020) from our group found specifically FC between the FPN and the DMN associated with a cognitive component reflective of verbal fluency and memory performance in older adults (age range: 55–85) from the 1000BRAINS study. Using the modality of brain structure, another study (Peitz et al. 2023) found longitudinal evidence for the association between gray matter volume of the FPN and language abilities, that is, bilingualism, across the life span (age range: 19–79), both lending support to the involvement of the DMN and FPN in language abilities.

Especially in older adults, more than just the classic perisylvian language network appears to contribute to language abilities. Research findings challenge the traditional view of a clearly restricted core language network and instead point to a network‐based approach in which multiple systems contribute to language performance (Fedorenko and Thompson‐Schill 2014). On the one hand, the classical language system is extended by additional frontal (e.g., middle and superior frontal lobes) and parietal regions of the FPN. On the other hand, networks that control supporting cognitive processes, such as the right FPN and the DMN, could play a role. Therefore, functional network patterns of both networks—the DMN and FPN—including their right hemispheric homologs may provide valuable information about cognitive language performance across the lifespan.

Nevertheless, it should also be acknowledged that language processing is not only subserved by activity in the grey matter, but also crucially depends on white matter pathways, that is, SC (Friederici and Gierhan 2013; Turken and Dronkers 2011). For instance, SC measured by streamline counts in the whole brain, the cingulum bundle, the fornix, and left superior longitudinal fascicle III have been associated with language performance, for example, picture vocabulary score and semantic memory task, in two large samples of young adults from the Human connectome project (HCP) (Lin et al. 2020; Zekelman et al. 2022). Thus, it appears that SC in fibre bundles, which have been associated with the DMN and FPN, appears to be linked to verbal performance (Hirsiger et al. 2016; Li et al. 2016). Along the lines in neurodegenerative disorders, diffusion‐weighted imaging measurements, for example, fractional anisotropy, radial diffusivity, and streamline counts from the cingulum and parahippocampal bundles were found to predict performance in memory, attention, working memory (WM) and, importantly, language in mild Alzheimer's Disease (AD) (Weiler et al. 2014) as well as to differentiate between patients with mild cognitive impairment who convert to AD and nonconverters (Magalhães et al. 2022). Hence, it can be argued that not only FC, but also SC of specific fiber bundles that are part of networks relevant for language, including the DMN and FPN, may be linked to language abilities across the lifespan in both health and disease. Thus, both FC and SC may be potentially informative of differences in verbal abilities at the individual level across the lifespan.

Language functions are essential for us to engage with and navigate the world. They have not only been related to other cognitive functions and been implicated in neurodegenerative disorders, but have also been linked to levels of quality of life across the life span (Beltrami et al. 2018; Boyle et al. 2021; Constantinidou et al. 2015; Eyigoz et al. 2020; Orimaye et al. 2017). Thus, maintenance of verbal abilities and the prevention of significant decline in these abilities appears similarly desirable to that of nonverbal abilities, for example, executive functions (Heckner et al. 2023), over time. A first step in this direction would be the development of a marker that captures language functioning in different age groups to prospectively identify those who might be most vulnerable to decline, enabling early interventions (Tomasi and Volkow 2020). In this context, machine learning (ML) approaches may be useful tools to address this task given their ability to deal with high‐dimensional data and to derive individual‐level predictions (Davatzikos 2019; Orrù et al. 2012; Varoquaux and Thirion 2014). Initial studies so far have reported mixed prediction results of verbal abilities for particular age groups, modalities, and functions. For example, language functions, such as reading and vocabulary comprehension, could be successfully predicted from task‐based and resting‐state functional connectivity, particularly in the DMN and FPN, in large samples of young adults using different ML approaches (Jiang et al. 2020b; Tomasi and Volkow 2020). In turn, findings from two multimodal studies in young adults from the HCP showed that FC and SC data may predict crystallized abilities, that is, picture vocabulary performance and reading recognition, better than fluid abilities, that is, executive function and processing speed (Dhamala et al. 2021; Rasero et al. 2021). Turning to findings in older adults, Kwak et al. (2021) showed that performance on a semantic fluency task could be predicted from FC measures with accuracies ranging between *r* = 0.18–0.28 in a large sample from the OASIS‐3 project (age range: 42–95 years).

In contrast, language abilities (*r* = 0.12–0.23) could be predicted to a smaller degree compared to executive functions and attention (*r* = 0.25–0.37) from SC in two large cohorts across the life span, that is, BARBI and HCP‐A (age range: 36–100) (Feng et al. 2022). Along these lines, prior findings from our group suggested that different domain‐specific cognitive profiles, including a verbal memory and language component, could be predicted only to a limited extent from FC estimates alone and from multimodal imaging data, that is, grey matter volume, FC, and SC estimates, in older adults from the 1000BRAINS study (age range: 55–85) (Krämer et al. 2023, 2024). Thus, it appears that specific language abilities may be successfully predicted from specific modalities and in specific age groups. Nevertheless, it remains challenging to draw more general conclusions and reconcile the different findings across studies due to differences in language targets, age groups, modalities, and ML frameworks used. To generalize findings and make more universal claims about the predictability of language functions from imaging data across the life span, it seems warranted to address this topic in a common ML framework and cohort and to systematically compare prediction performance for verbal abilities across age groups, imaging modalities, and feature sets.

The current study thus aimed at an in‐depth investigation of the predictability of language abilities from structural and functional connectivity data across the life span and different analytic choices using an ML approach. Specifically, it set out to examine if predictability differences emerge between (a) verbal fluency and vocabulary knowledge, (b) FC and SC, (c) a network‐specific, that is, FPN and DMN, and whole‐brain approach, as well as (d) the whole life span, younger and older adults across different ML algorithms in a large sample from the 1000BRAINS study.

## 2 Methods

### 2.1 Study Population

The research question was investigated in a sample from the 1000BRAINS study (Caspers et al. 2014), which is a population‐based cohort study that recruited 1314 subjects from the German Heinz Nixdorf Recall Study and the Heinz Nixdorf Recall MultiGeneration Study (Schmermund et al. 2002). The main analysis was conducted in a subset of 717 participants (327 females; Mean_age_ = 59.1; SD_age_ = 13.6; age range: 18–85). Participants were excluded from the study due to the following criteria: (i) missing values for demographic data (6), (ii) missing DemTect score (18), (iii) at least one or more missing scores on the predicted cognitive tasks (46), (iv) DemTect Score ≤ 8 indicating signs of dementia or mild cognitive impairment (Kalbe et al. 2004) (9), (v) missing brain data, preprocessing failure, or abnormalities in imaging data detected by quality control (280), (vi) outlier scores defined as participants scoring ±3 SD from the mean in at least one of the cognitive variables (35), and (vii) family relationship (in case of more than one person per family, only one family member was included in the current analysis) (203). To investigate the impact of aging on the predictability of language functions, subjects were divided into two groups based on age, that is, younger (< 60 years of age; *N* = 311, 142 females, Mean_age_ = 46.9 ± 10.9, Mean_ISCED_ = 7.2 ± 1.7) and older (> 60 years of age, *N* = 406, 185 females, Mean_age_ = 68.4 ± 5.7, Mean_ISCED_ = 6.5 ± 2.0). The cut‐off at age 60 between the two groups was chosen as performance loss within the chosen language tasks appeared around the age of 60. This content‐related decision was based on a prior analysis to capture the time point at which age‐related decline in language functions starts to emerge. The study protocol of 1000BRAINS was approved by the ethics committee of the University of Duisburg‐Essen, and all subjects provided written informed consent before inclusion. The study procedures comply with the Declaration of Helsinki (Table 1).

**TABLE 1:** Demographic information of the total, old, and young sample.

	N	Age (SD)	Age range	Edu (SD)	DemTect (SD)
Total	717 (390 M, 327 F)	59.1 (13.6)	18–85	6.8 (1.9)	15.3 (2.4)
Younger	311 (169 M, 142 F)	46.9 (10.9)	18–59	7.2 (1.7)	15.5 (2.4)
Older	406 (221 M,185 F)	68.4 (5.7)	60–85	6.5 (2.0)	15.1 (2.3)

*Note:* Edu = education level measured by ISCED; F = females; M = males.

### 2.2 Cognitive Performance

All subjects underwent extensive neuropsychological testing through their participation in the 1000BRAINS study (Caspers et al. 2014). For the investigation of verbal functioning, we focused on vocabulary knowledge, which taps into crystallized intelligence, and verbal fluency, which addresses more fluid abilities. In this context, it should be stressed that these two functions may be differentially impacted by the aging process. Fluid abilities, for instance, verbal fluency, appear to be more strongly impacted by aging than crystallized abilities such as vocabulary (Baciu et al. 2016; Gonzalez‐Burgos et al. 2019). The Word‐hoard‐test was used as a measure of vocabulary (VOC) knowledge and word recognition (Schmidt and Metzler 1992). In the test, participants were asked to find one meaningful word among five nonsense, distractor words. For the operationalization of language production, the Regensburger Wortflüssigkeitstest (Aschenbrenner et al. 2000) was used, to assess verbal fluency. Both phonematic (PF) and semantic verbal fluency (SF) were measured. In the semantic condition, participants were asked to name as many words as possible within the category professions (in German: Berufe) in two minutes. In the phonematic condition, subjects were required to produce as many German words as possible beginning with the letter B in 2 min. The two conditions, that is, PF and SF, were used separately as targets in the ML pipeline due to differential behavior throughout the aging process and their involvement of distinct brain regions (Gonzalez‐Burgos et al. 2019; Vigneau et al. 2011; Wagner et al. 2014). To capture general verbal fluency abilities, a combined language production score, that is, a verbal fluency (VF) score, was calculated as the arithmetic mean of the PF and SF scores. Along the same lines, a composite language performance score (VER) was derived by combining performance on the vocabulary and verbal fluency tests to assess general language abilities, which feature both crystallized and fluid abilities. Finally, all scores were standardized and transformed into Z‐scores for each sample separately.

To evaluate if prediction results are specific to verbal functions, we also investigated the predictability of two nonverbal cognitive measures, that is, processing speed and visual working memory, which have been frequently reported to be successfully predicted from connectivity data across various samples (Avery et al. 2020; Dhamala et al. 2021; Gao et al. 2020; Pläschke et al. 2020). As a measure of processing speed, the Trail Making Test A (TMT‐A) was chosen, in which participants are asked to connect numbers with lines in ascending order, and the time to complete the task is taken (Morris et al. 1989). For interpretation purposes, scores were inverted to indicate higher scores equal better performance as well as standardized using Z‐scores similar to the language scores. Furthermore, we examined a visual working memory score (vWM) based on the arithmetic mean of the performance on three different tasks, that is, Corsi Block Tapping Test (CBTT), Benton Test (BT), and the Visual Pattern Test (VPT) (Benton et al. 2009; Della Sala et al. 1997; Schelling 1997). In the CBTT, participants are asked to reproduce a tapping sequence demonstrated by the researcher in reverse order (Schelling 1997). In the BT, participants are shown 20 geometric patterns and asked to reproduce those exactly from memory (Benton et al. 2009). The performance score is measured as the number of errors. In the VPT, participants are asked to reproduce a pattern of black and white squares arranged in a matrix on a blank grid (Della Sala et al. 1997). The performance score is represented by the number of correct solutions. Since scales differed between the three tests, all scores were Z‐score normalized similar to the language scores. Scores on BT were inverted, as higher scores indicated worse performance.

### 2.3 MRI Data Acquisition

The 1000BRAINS data set comprises a detailed magnetic resonance imaging (MRI) protocol with structural and functional data. All MR imaging was performed using a 3 Tesla Siemens Tim‐TRIO scanner with a 32‐channel head coil. Structural scans include a 3D T1‐weighted MPRAGE sequence and three diffusion‐weighted sequences. For the SC analysis, high‐angular resolution diffusion imaging (HARDI) data with the following parameters were used: (i) 120 directions dataset, EPI, TR = 8 s, TE = 112 ms, 13 b_0_‐images (interleaved), 120 images with *b* = 2700 s/mm^2^, voxel resolution = 2.4 × 2.4 × 2.4 mm^3^; (ii) 60 direction subset (out of 120 direction dataset), EPI, TR = 6.3 s, TE = 81 ms, 7 b_0_‐images (interleaved), 60 images with *b* = 1000s/mm^2^, voxel resolution = 2.4 × 2.4 × 2.4 mm^3^. For the surface reconstruction, a T1‐weighted magnetization prepared rapid acquisition gradient‐echo (MPRAGE) anatomical scan was used: 176 slices, slice thickness = 1 mm, repetition time (TR) = 2250 ms, echo time (TE) = 3.03 ms, field of view (FoV) = 256 × 256 mm^2^, flip angle = 9°, voxel resolution = 1 × 1 × 1 mm^3^ (Caspers et al. 2014). Functional resting state scans were acquired for about 11:30 min with closed eyes, while participants were asked to let their minds wander without thinking of anything in particular. A blood‐oxygen‐level‐dependent (BOLD) gradient echo planar imaging (EPI) sequence with 36 transversally oriented slices with the following specification was used: slice thickness = 3.1 mm, TR = 2200 ms, TE = 30 ms, FoV = 200 × 200 mm^2^, voxel resolution = 3.1 × 3.1 × 3.1mm^3^ (Caspers et al. 2014).

### 2.4 Image Processing to Derive Structural Connectivity Data

T1‐weighted imaging data was used to compute tissue probability maps for grey matter, white matter, and corticospinal fluid using the Computational Anatomy Toolbox (CAT12) (Gaser and Dahnke 2016). By superimposing these three probability maps and thresholding them at 0.5, the brain could be extracted from the T1 data. This brain image was then bias field corrected, rigidly aligned to MNI152 template space, and resampled to 1.25 mm isotropic voxel size. Diffusion MRI data was corrected for eddy current and motion artifacts (Andersson et al. 2016) and visually quality controlled for ghosting, remaining signal dropout, and noisy data. Based on Wells et al. (1996), dMRI‐T1 alignment was performed by extracting the first b0‐images from each dMRI data with b1000 and b2700 and rigidly aligning them to the T1 dataset using mutual information as a cost function. Consequently, there were two separately registered diffusion images for the two *b*‐values. Anisotropic Power Maps (APM; Dell'Acqua et al. 2014) were calculated as a basis for image registration. These APMs were subsequently used to compute the nonlinear transformation from diffusion to anatomical space, as well as to transform the tissue probability maps to diffusion space. After the b1000 and b2700 data sets were merged into one, they were corrected for different echo times through a voxel‐wise multiplication of the b2700 data with the ratio of the nondiffusion‐weighted data. The MRtrix software (Tournier et al. 2012) was used for local modeling and probabilistic streamline tractography. Constrained Spherical Deconvolution (CSD) was performed using multi‐tissue CSD of multi‐shell data (Jeurissen et al. 2014), with all shells and a maximal spherical harmonic order of 8. Using the probabilistic iFOD2 algorithm with a maximal length of 250 mm and a cut‐off value of 0.06, 10 million streamlines were computed for each subject.

### 2.5 Image Processing for Functional Connectivity Data

For functional image preprocessing, the FSL toolbox (Jenkinson et al. 2012) was used. To remove the influence of the scanner magnetization not being stabilized, the first four volumes for each participant were discarded (Soares et al. 2016). In addition, functional images were corrected for head movement by first aligning all images to the first image. Based on this alignment, a mean image was created, to which then all volumes were aligned. ICA‐based Automatic Removal Of Motion Artifacts (ICA‐AROMA) (Pruim et al. 2015), combined with global signal regression, was used as a second step to identify all motion‐related components. After bandpass filtering (0.01–0.1 Hz) to remove physiological and scanner‐related noise, fMRI images were registered to the standard space template (MNI152) using the Nonlinear Image Registration tool FNIRT. Furthermore, images were checked for each participant's volume‐wise severe intensity dropouts through the generation of *p* values for spikes (Afyouni and Nichols 2018). Participants for which more than 10% of a total of 300 volumes were classed as dropouts were excluded. Finally, images were checked for potential misalignment by using the “check sample homogeneity using standard deviation across sample”‐function provided by the CAT12 toolbox (Gaser and Dahnke 2016). Participants marked as outliers were excluded.

### 2.6 Parcellation

The brain was parcellated into 400 cortical regions using the Schaefer parcellation (Schaefer et al. 2018). These 400 parcels can be assigned to the 7 network parcellation (Yeo et al. 2011), comprising the visual (VN), sensorimotor (SMN), limbic (LN), frontoparietal (FPN), default mode (DMN), dorsal (DAN), and ventral attention network (VAN). For SC, nonlinear warps of the spatial T1 registration were combined with an MNI152 template and distortion correction with the APMs to warp the parcellation template to the individual diffusion space. Then, streamline counts between each pair of nodes were converted into weighting factors using SIFT‐2 (Smith et al. 2015) as a cross‐sectional area multiplier, and all values were log_10_ transformed. For FC, the mean time series of the preprocessed resting‐state fMRI data were extracted node‐wise. Here, the time series of all voxels corresponding to one node were averaged. To calculate the connectivity between two nodes, Pearson's product–moment correlation of the respective average BOLD time series was used. The observed time series were randomized by taking its Fourier transform, scrambling its phase, and then inverting the transform. This whole procedure was repeated 1000 times, and a permutation test was performed, aiming at minimizing the number of edges caused by noise. Nonsignificant edges were set to 0. The resulting matrix was Z‐score transformed through Fisher r‐to‐z transformation, with positive as well as negative correlations. Both positive and negative edges were included as previous research has suggested that despite problems with interpretability, negative correlations may carry meaningful biological information (Fox et al. 2005; Sporns and Betzel 2016). For more details on connectivity data and the parcellation procedure, please refer to Stumme et al. (2022). No further thresholding of the connectivity matrices was performed.

Input to ML constituted the connectivity strength (FC: Fisher r‐to‐z‐transformation correlation coefficients; SC: SIFT‐2 transformed streamline counts) between each pair of nodes in different feature sets. Due to the role of FPN and DMN in language functions and their importance for the prediction of language abilities, the current analysis focused on connectivity data from these two language‐related networks (i.e., FPN [52 nodes], DMN [91 nodes]) in comparison to whole‐brain connectome data (Dick et al. 2010; Jiang et al. 2020a; Tomasi and Volkow 2020). Connectivity data from both hemispheres was used in the analyses. While a strong left hemispheric dominance has been reported for language abilities, information from the right hemisphere may also be relevant, particularly in light of aging processes and compensatory mechanisms (Agarwal et al. 2016; Cabeza 2002; Hoyau et al. 2018) as more deeply discussed in the introduction. For a schematic overview of the parcellation (i.e., network plots) and a detailed description of the nodes, please refer to the Supplement. In total, this resulted in the extraction of the following four feature sets from the connectivity matrices (including only information from the upper triangle of the matrix; N(N−1)/2) for both modalities to be used in ML: (1) all connections of the FPN (# of features = 1326), (2) all connections of the DMN (# of features = 4095), (3) all connections in FPN and DMN (# of features =10,153), and (4) connections in the whole brain (# of features =79,800).

### 2.7 ML Prediction Framework

**FIGURE 1:**
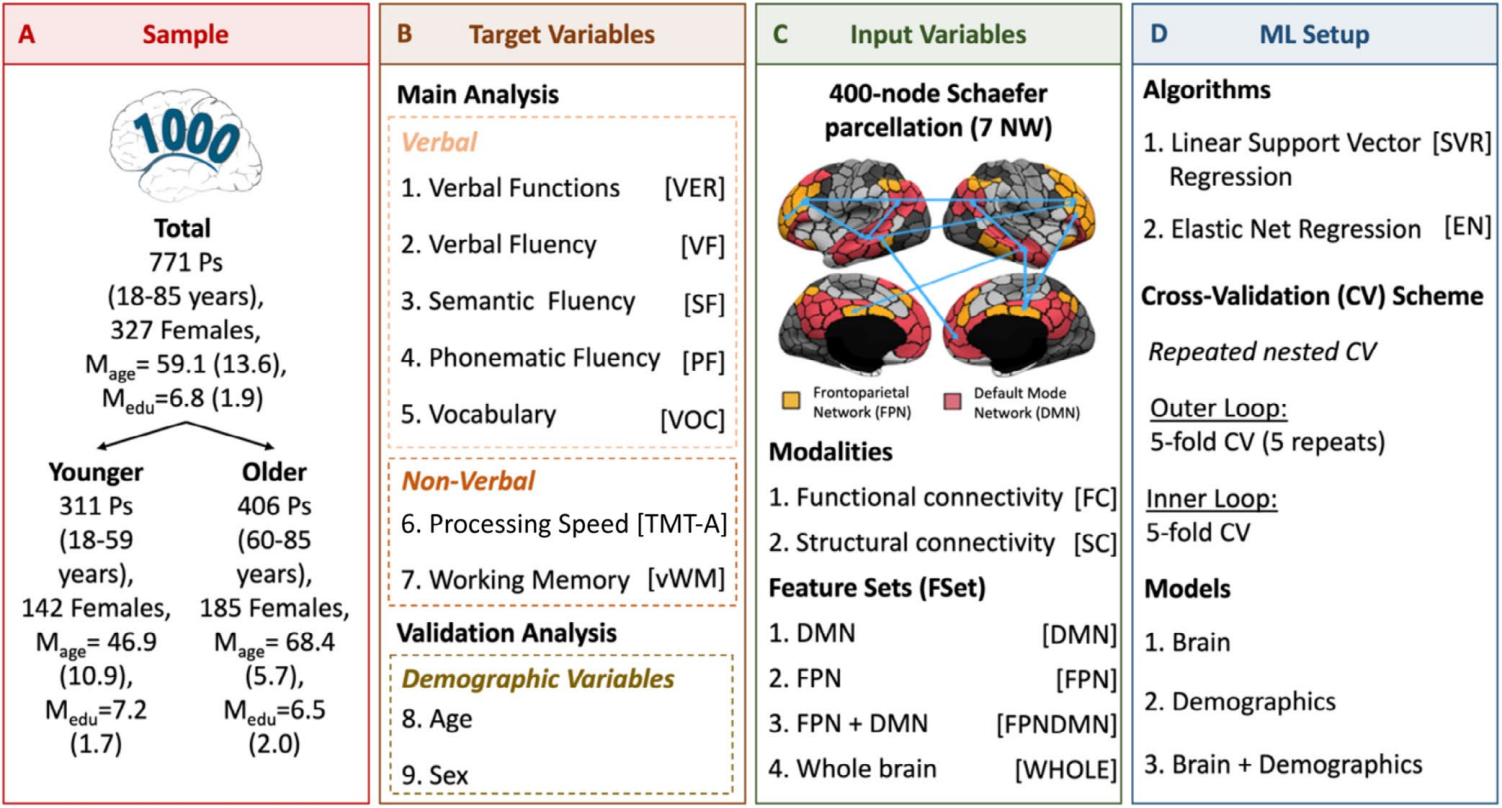
Schematic overview of workflow.

To compare differences in predictability of distinct language functions, an ML approach was used. A schematic overview of the workflow is shown in Figure 1. All ML analyses were performed using the scikit‐learn library (Version: 0.22.1) in Python (Pedregosa et al. 2011). Prediction performance for verbal (i.e., VF, SF, PF, VOC, VER) and nonverbal cognitive targets (i.e., PS, vWM) was examined for two modalities, that is, FC and SC, and four feature sets, that is, DMN, FPN, FPNDMN, and WHOLE brain (Figure 1B,C). Predictions for all combinations of input features and targets were obtained separately for the total sample, the younger, and older groups (Figure 1A). A focused comparison of two well‐established ML algorithms was conducted as the efficacy of different ML regression algorithms for different imaging modalities is still not fully elucidated (Jollans et al. 2019): linear Support Vector Regression (SVR) and Elastic Net (EN) regression, which were selected since they are commonly used in the literature (Figure 1D) and include different degrees of feature selection (Cui and Gong 2018; Jollans et al. 2019). While SVR does not inherently include feature selection, EN incorporates an embedded feature selection step through regularization (Jollans et al. 2019). Thus, this allows the assessment of the impact of the inclusion of a feature selection step on ML results. No additional feature selection was performed. For each input–output combination, a separate SVR and EN model was trained.

For performance estimation, a repeated nested 5‐fold cross‐validation (CV) was employed (5 repeats). All hyperparameters were optimized within the inner CV loop (5‐fold) to avoid data leakage. The following hyperparameters were optimized in the inner folds: (i) regularization parameter C for SVR {C: 10^−4^ to 10^1^, 10 steps, logarithmic scale}, (ii) the regularization parameter lambda, λ, and alpha, α, for EN (λ:10^−1^ to 10^2^, 10 steps, α: 0.1–1, 10 steps). Prediction performance was assessed using the following measures: the coefficient of determination (*R* ^2^) and the Mean Absolute Error (MAE). For completeness, the Pearson's correlation between true and predicted scores (*r*) is reported as well and can be found in the Supplement.

### 2.8 Confounder Analyses

ML performance may be influenced by different confounding variables, such as age, sex, and education. To assess their impact on prediction results, additional analyses were performed in the current study. Particularly, demographic variables were used as extra features for our ML models following previous studies (Dadi et al. 2021; Krämer et al. 2024; Rasero et al. 2021). Thus, we additionally investigated the predictability of cognitive targets from age, sex, and education alone as well as jointly with brain connectivity data. Finally, we compared prediction performance across these different models (see Figure 1D).

### 2.9 Validation Analysis

To validate the ML‐based prediction approach, two well‐investigated demographic variables, that is, age and sex, were predicted from the brain data. Prior research has shown that both variables could be reliably predicted from connectivity data and, thus, may be used to assess whether similar results can be achieved using the ML framework and the same data (Dhamala et al. 2020; Lancaster et al. 2018; H. Li et al. 2018; Liem et al. 2017; Vergun et al. 2013; Weis et al. 2020). Age and sex predictions were carried out following the steps outlined in the main analysis, with the only exception that for sex classification a Support Vector Classifier (SVC) and Ridge Classifier were used, and ML performance was measured by accuracy (Acc.). Furthermore, extreme group classifications were performed based on FC and SC data (same as in the main analysis) to validate our findings in the total, younger, and older samples. Thereby, 25% of the top and lowest scorers on each language variable were selected as extreme groups (Krämer et al. 2024). For additional information on the extreme groups, please refer to Table S4. As for the sex classification, classification accuracies were compared across SVC and a Ridge classifier. To assess the influence of parcellation resolution on prediction results, we also investigated ML performance based on FC and SC data from a more fine‐grained parcellation (i.e., 800‐node Schaefer parcellation). Analyses were carried out for all cognitive variables in the total sample using network‐specific data and the same ML approach as in the main analysis. To investigate the impact of hemispheric specialization (e.g., the left hemisphere appears to be particularly important for language functions) on prediction results, we also carried out ML analyses for each cognitive variable based on connectivity data from the left versus right hemisphere in the total, younger, and older sample. As the total intracranial volume (TIV) may have a substantial impact on prediction results, we further performed analyses in which we controlled for estimated TIV. Specifically, we regressed eTIV out of the target within the CV scheme and re‐ran all analyses of the main analyses (Krämer et al. 2024; Rasero et al. 2021). As eTIV was not available for all participants, validation analyses were performed on a slightly smaller sample (*N* = 697, 323 females, Mean_age_ = 59.18 ± 13.47, Mean_ISCED_ = 6.84 ± 1.89).

### 2.10 Statistical Analyses

Statistical analyses were performed using R (R Core Team 2024) and Python (version 3.12.4). Correlations between connectivity data, cognitive tests as well as demographic variables were calculated as Pearson's correlations, and independent samples *t*‐tests were used to investigate sex and age differences in cognitive tests and connectivity in the total sample. To reduce the number of statistical tests, connectivity data was aggregated into graph‐theoretical network parameters (Krämer et al. 2023; Stumme et al. 2020). Specifically, within‐network connectivity was extracted for the DMN and FPN for both functional and structural connectivity data. Additionally, between‐network connectivity between the DMN and FPN was computed and used in the statistical analyses (Krämer et al. 2023; Stumme et al. 2020). For a more detailed description of the extraction of the network parameters, please refer to Stumme et al. (2020).

## 3 Results

### 3.1 Relationship Between Cognitive and Demographic Variables

Mean performance scores on cognitive tests can be found in Table 2. Correlational analysis showed that performance on all cognitive tests decreased significantly with age, except for vocabulary (Table 3). The strongest negative associations with age were found for nonverbal abilities, that is, processing speed and visual working memory. Concerning language‐related tests, semantic fluency was most strongly negatively correlated with age (Tables 3, S5, and Figure S2). Closer examination of age‐test performance scatter plots revealed that cognitive performance became more variable with higher ages (Figure S2). From the scatter plots, no floor or ceiling effects could be observed in the data (Figure S2). Independent samples t‐tests further support these results. Younger and older participants significantly differed from each other in all cognitive tests (*t*(628–710) = −16.1 to −2.20, *p* < 0.05; see Table S6). Older participants showed lower performance on verbal and nonverbal cognitive tests compared to younger participants (see Table S5 and Figure S2). Education levels showed positive associations with test performance, that is, better test performance was associated with a higher educational level (Table 3). Males and females differed significantly in their performance on the following cognitive tests: VOC, CBTT, BT, and VPT (*t*(692–714) = −6.09 to −2.70, *p* < 0.05). In all cases, males outperformed females.

**TABLE 2:** Mean cognitive performance on all cognitive tests across samples.

	SF	PF	VOC	PS	CBTT	BT	VPT
Total	25.6 (7.0)	19.4 (6.0)	31.6 (3.8)	34.4 (11.9)	10.0 (1.1)	13.3 (7.8)	8.6 (2.1)
Males	25.6 (7.4)	19.0 (5.9)	32.0 (3.8)	34.6 (12.5)	5.1 (1.1)	12.6 (7.8)	9.0 (2.0)
Females	25.5 (6.4)	19.9 (6.1)	31.2 (3.8)	34.2 (11.1)	4.8 (1.0)	14.3 (7.8)	8.1 (2.0)
Younger	27.6 (6.7)	20.4 (5.9)	32.0 (3.5)	27.9 (9.3)	5.4 (1.1)	9.3 (6.2)	9.7 (1.9)
Older	24.6 (6.8)	18.7 (6.0)	31.4 (4.0)	39.4 (11.2)	4.7 (1.0)	16.5 (7.5)	7.7 (1.7)

*Note:* Standard deviations (SD) are displayed in parentheses.

**TABLE 3:** Pearson's correlations between cognitive tests and demographic variables in the total sample.

	SF	PF	VF	VOC	VER	PS	CBTT	BT	VPT	vWM
Age	−0.25**	−0.14**	−0.23**	−0.06	−0.20**	−0.59**	−0.35**	−0.53**	−0.53**	−0.58**
Edu	0.27**	0.23**	0.30**	0.48**	0.42**	0.19**	0.17**	0.34**	0.29**	0.33**

*Note:* **p < 0.001.

**FIGURE 2:**
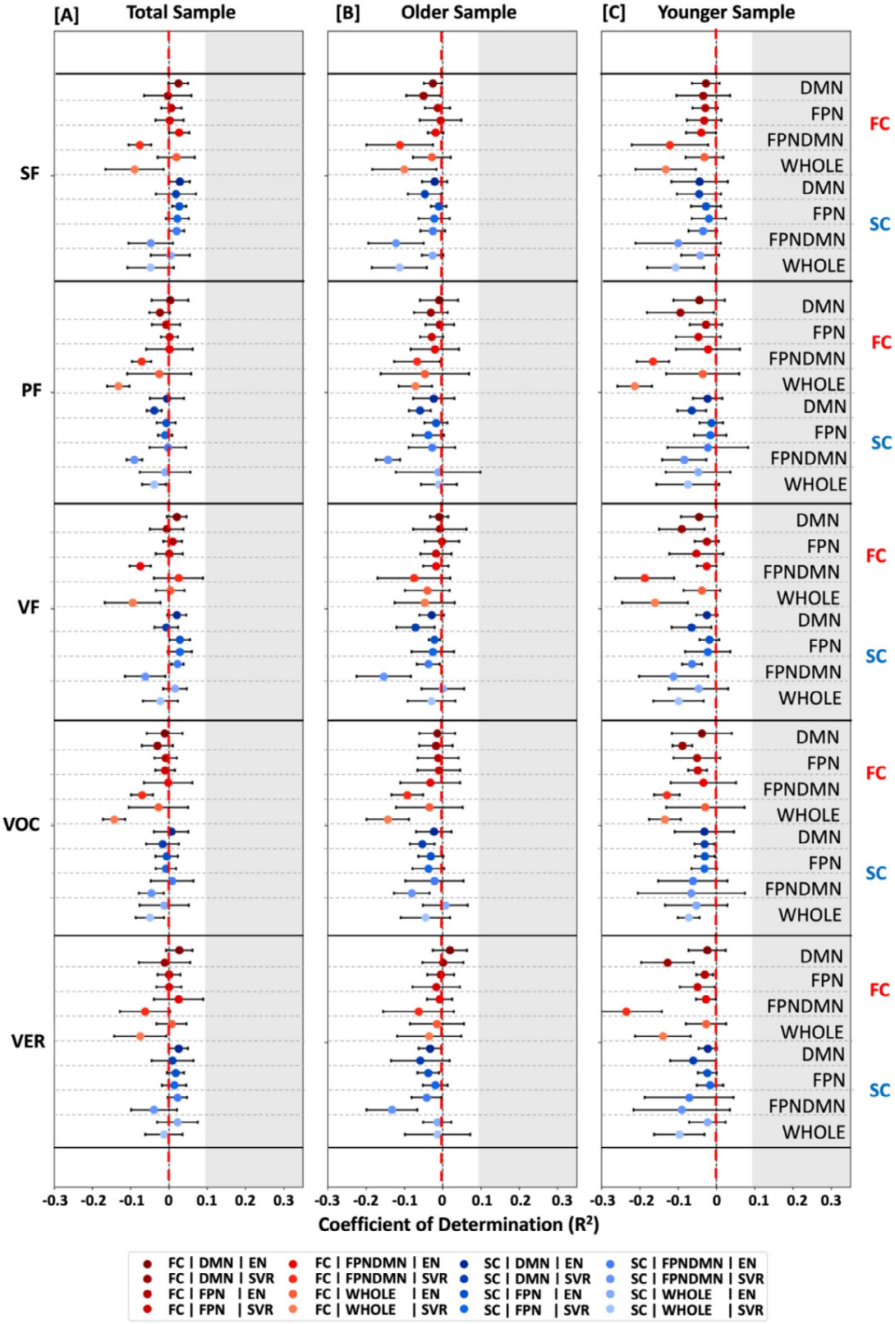
Prediction results for language performance tests. The mean coefficient of determination (*R* ^2^) across folds is displayed for the prediction of verbal performance, that is, semantic fluency (SF), phonematic fluency (SF), combined verbal fluency (VF), vocabulary (VOC) and combined verbal functions (VER), from brain connectivity data, that is, FC (red) and SC (blue), in the default mode network (DMN), frontoparietal network (FPN), in both the FPN and DMN (FPNDMN), and in the whole brain (WHOLE). Results are shown separately for the total [A], old [B], and the young [C] samples. Error bars represent the standard deviation (SD). The following algorithms were used for prediction: Elastic Net (EN) regression and linear Support Vector Regression (SVR). The grey shaded area indicates prediction performance *R* ^2^ > 0.1.

### 3.2 Relationship Between Connectivity Data and Cognitive Variables

Correlational analyses revealed that within‐network functional connectivity of the FPN was significantly correlated with verbal abilities, processing speed, semantic, and verbal fluency (r ranges = 0.09–0.11, *p* < 0.05; Table S5 and Figure S3). All other correlations for FC were found to be nonsignificant. In terms of SC, within‐ and between‐network connectivity of the FPN and DMN were significantly associated with all cognitive variables except for vocabulary (FPN) and phonematic fluency (DMN) (*r* ranges = 0.08–0.25, *p* < 0.05; Table S3 and Figures S4 and S5). In all cases, higher connectivity was associated with better performance.

### 3.3 Relationship Between Connectivity Data and Age

Correlational analyses revealed that age was significantly negatively correlated with all SC network parameters (*r* ranges = −0.31 to −0.42, *p* < 0.001, see Table S5 and Figure S6). As such, within‐network SC was found to decrease with higher ages. No significant associations were discovered for FC network parameters. An independent samples *t*‐test further corroborated these results. Significant differences between the younger and older groups were only encountered for SC parameters (*t*(715) = 7.7–10.6, *p* < 0.001, Table S7). In this context, older participants displayed lower connectivity both within‐ and between‐networks compared to younger participants (Table S7 and Figure S6).

### 3.4 Prediction of Language Functions

The primary aim of this study was to examine the predictability of verbal abilities, that is, verbal fluency and vocabulary, from brain connectivity data, that is, FC and SC, across the life span. Overall, prediction performance was found to be very limited for the different language functions across analytic choices (Figure 2). In this context, models were found to explain nearly no or no variance in the different language targets (SC: *R* ^2^ range = −0.15 to 0.03; FC: *R* ^2^ range = −0.24 to 0.03; Figure 2, Tables S8–S15) across different groups, algorithms, and feature sets. Furthermore, predictability did not seem to differ between the total sample (*R* ^2^ range = − 0.13 to 0.03), the older (*R* ^2^ range = − 0.15 to 0.01), and younger (*R* ^2^ range = −0.24 to 0.01) groups. Along the same lines, merging the three investigated language tests into a combined verbal score (Combined: *R* ^2^ range = −0.12 to 0.03; individual: *R* ^2^ range = −0.24 to 0.03; Figure 2, Tables S8–S15) and using whole‐brain instead of network‐specific information (whole‐brain: *R* ^2^ range = −0.16 to 0.02; network‐specific: *R* ^2^ range = −0.24 to 0.03; Figure 2, Tables S8–S15) resulted in similarly low results. Thus, it appeared that language production and comprehension could not be successfully predicted from brain connectivity data across different age groups (i.e., total vs. older vs. younger), algorithms (i.e., EN vs. SVR), feature sets (i.e., network‐specific vs. whole brain), and modalities (i.e., FC vs. SC) in the current sample from the 1000BRAINS study.

### 3.5 Comparison of Predictability Between Nonverbal Cognitive Measures and Verbal Measures

**FIGURE 3:**
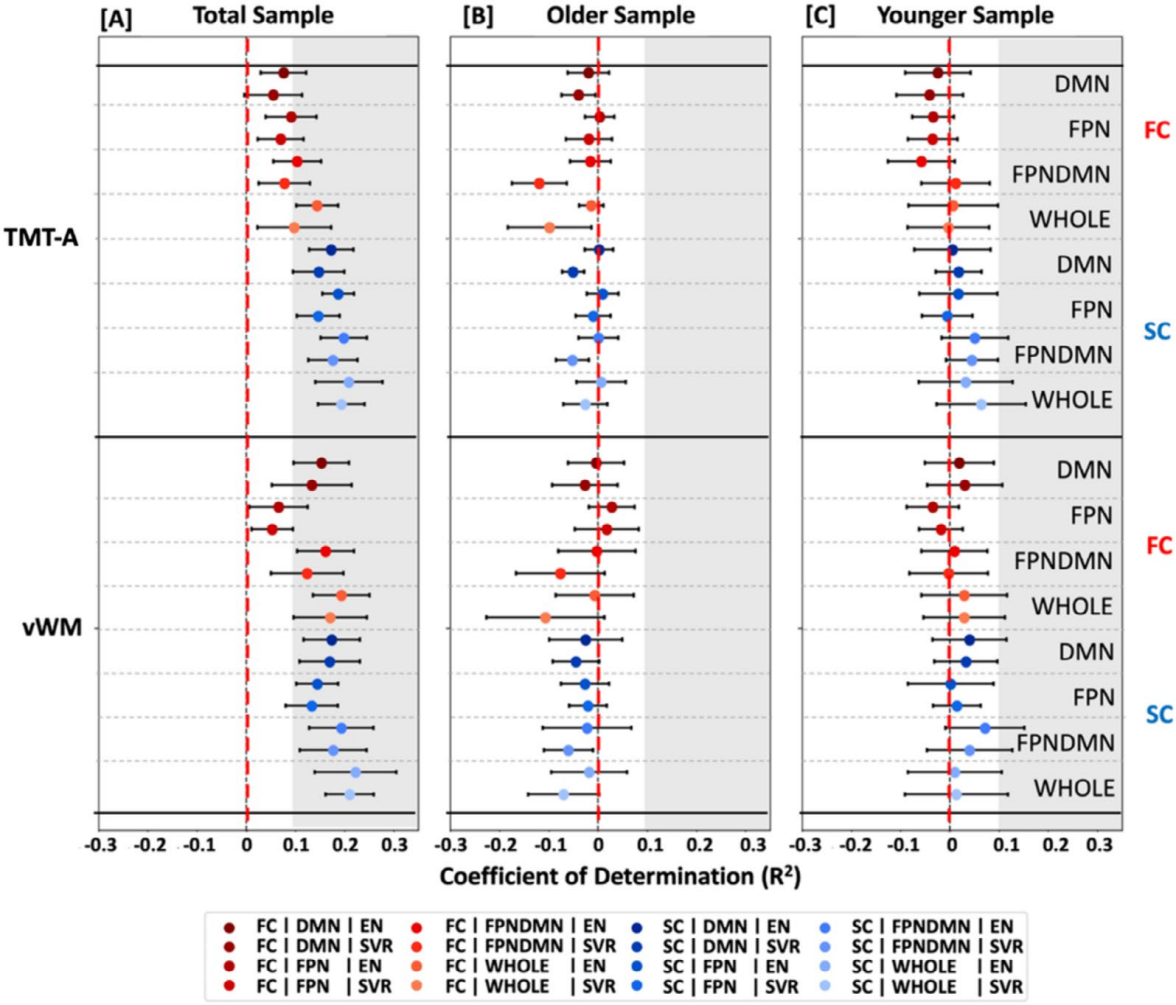
Prediction results for nonverbal cognitive tests. The mean coefficient of determination (*R* ^2^) across folds is displayed for the prediction of processing speed, that is, TMT‐A performance, and visual working memory, that is, vWM, from brain connectivity data, that is, FC (red) and SC (blue), in the default mode network (DMN), frontoparietal network (FPN), in both the FPN and DMN (FPNDMN) and in the whole brain (WHOLE). Results are shown separately for the total [A], old [B], and the young [C] samples. Error bars represent the standard deviation (SD). The following algorithms were used for prediction: Elastic Net (EN) regression and linear Support Vector Regression (SVR). The grey shaded area indicates prediction performance *R* ^2^ > 0.1.

To assess whether these results of nonreliable predictability of language functions were specific to these functions, we further investigated the predictability of nonverbal cognitive measures, that is, processing speed and visual working memory, from connectivity data across age groups, modalities, feature sets, and algorithms. Across analytic options, nonverbal cognitive measures could be better predicted than verbal cognitive measures (Nonverbal: *R* ^2^ range = −0.12 to 0.22; Verbal: *R* ^2^ range = −0.24 to 0.03; Figures 2 and 3, Tables S8–S15). Focusing on nonverbal cognitive functions, predictability differences between groups emerged. Nonverbal cognitive measures could be better predicted in the younger (*R* ^2^ range = −0.09 to 0.07) compared to the older group (*R* ^2^ range = −0.12 to 0.03) and best predicted in the total sample (*R* ^2^ range = 0.05–0.22, Figure 3A, Tables S8–S15) across modalities, feature sets, and algorithms. In the total sample, processing speed and visual working memory could be predicted to a greater extent from SC (*R* ^2^ range = 0.13–0.22) compared to FC (*R* ^2^ range = 0.05–0.19, Figure 3A, Tables S8–S15) and from whole brain (*R* ^2^ range = 0.10–0.22) compared to network‐specific (*R* ^2^ range = 0.05–0.20, Figure 3A, Tables S8–S15) information across algorithms and feature sets. In the younger and older group, differences between modalities (FC: *R* ^2^ range: −0.12 to 0.03; SC: *R* ^2^ range = −0.07 to 0.07) and feature sets (network‐specific: *R* ^2^ range = −0.12 to 0.07; whole‐brain: *R* ^2^ range = −0.11 to 0.06; see Figure 3B,C, Tables S8–S15) were found to be marginal. Hence, it seemed that nonverbal cognitive abilities could be moderately predicted from SC and FC data across analytic choices. This was particularly evident for the total sample and the younger group. In the older group, prediction performance was found to be rather limited.

### 3.6 The Impact of Demographic Variables on ML Predictions

**FIGURE 4:**
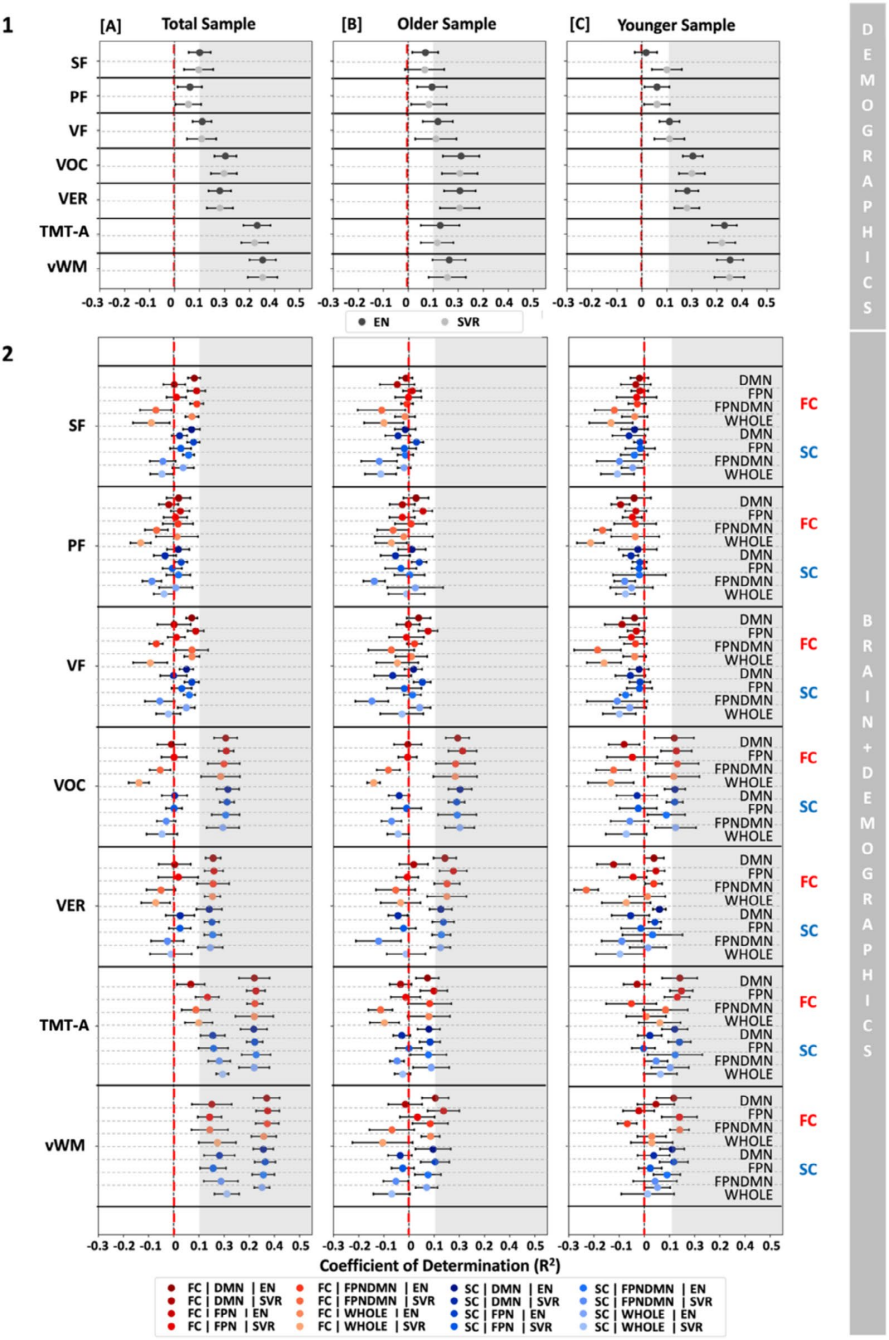
Prediction results for nonverbal and verbal cognitive tests from demographic variables [1], that is, age, sex, and education, and from demographic variables and brain data, that is, FC (red) and SC (blue) [2] in the different feature sets, that is, DMN, FPN, FPNDMN, and WHOLE brain, across samples, that is, total [A], old [B], and young [C]. Error bars represent the standard deviation (SD). The following algorithms were used for prediction: Elastic Net (EN) regression and linear Support Vector Regression (SVR). The grey shaded area indicates prediction performance *R* ^2^ > 0.1.

To assess the impact of demographic variables, that is, age, sex, and education, on ML predictions, we included them as features in our ML models. Particularly, we investigated the predictability of cognitive targets from only demographic variables and in conjunction with brain features (Figure 4). Across analytic options, that is, algorithms and age groups, demographic variables could moderately predict vocabulary (*R* ^2^ range = 0.18–0.23), verbal functions (*R* ^2^ range = 0.18–0.21), processing speed (*R* ^2^ range = 0.11–0.33) and visual working memory (*R* ^2^ range = 0.16–0.39, Figure 4 (1) and Tables S8–S16). In contrast, verbal fluency, that is, SF, PF, and VF, could be predicted to a smaller extent from age, sex, and education across groups and algorithms (*R* ^2^ range = −0.04 to 0.13, see Figure 4 (1) and Tables S8–S16). Combining brain features and demographics did not seem to improve prediction performance (only demographics: *R* ^2^ range = −0.04 to 0.39; demographics and brain features: *R* ^2^ range = −0.21 to 0.37, Figure 4 (2) and Tables S8–S16). Along the same lines, currently employed brain features do not seem to add information beyond that of demographic features to the prediction of different cognitive measures. Furthermore, it potentially hints at the fact that not all cognitive functions are equally impacted by demographic variables.

### 3.7 Validation Analyses

To validate our ML pipeline, we performed age and sex predictions using the same method as in the main analysis. Across algorithms, modalities, and age groups, most feature sets successfully predicted age (*R* ^2^ range = −0.04 to 0.72). In this context, we found that the highest prediction performance was based on the whole brain (*R* ^2^ range = 0.05–0.72), followed by the combination of FPN and DMN (*R* ^2^ range = 0.03–0.62), the DMN (*R* ^2^ range = 0–0.60), and the FPN (*R* ^2^ range = −0.04 to 0.52; Table S17). Furthermore, SC tended to outperform FC in predicting age (FC: *R* ^2^ range = −0.04 to 0.48; SC: *R* ^2^ = 0.21–0.72; Table S17). Additionally, better prediction results were observed in the total group (*R* ^2^ range = 0.25–0.72) followed by the younger group (*R* ^2^ range = −0.04 to 0.45), and the older group (*R* ^2^ range = 0.01–0.37; Table S17). Turning to the sex classification, we found that generally between 57.8% and 86.7% of cases were classified correctly, which is in line with what has been reported in the literature (Dhamala et al. 2020; Weis et al. 2020). Best prediction accuracies could be achieved when predicting from the whole‐brain connectome (Acc. range = 68.6%–86.7%) compared to network‐specific information (Acc. range = 57.8%–83.0%) and from SC (Acc. range = 72.5%–86.7%) compared to FC (Acc. range = 57.8%–72.5%; Table S18). Overall, both age and sex could be predicted to similar extents in the current study as reported in prior literature. Thus, it appeared that low prediction accuracies in the main analysis are most likely not rooted in the currently chosen ML framework. It is more likely that low accuracies seem to be specific to the prediction of language function, their specific representation, or their relation to selected networks or currently chosen brain connectivity data. To validate our results from the main analyses, we further performed extreme group classifications based on the different verbal abilities in the total, younger, and older samples. Results further corroborated the findings from the main analyses. Overall, low classification accuracies were found across extreme groups in all three samples (Acc. range = 44%–65%). There was a slight trend for SC data (Acc. range = 44%–65%) leading to higher accuracies than FC data (Acc. range = 44%–61%; Tables S19–S23). No large differences emerged between samples. Thus, results emphasize the challenging nature of predicting or classifying language abilities based on currently chosen input data. To assess the impact of parcellation granularity, we additionally investigated whether using a more fine‐grained parcellation (i.e., 800‐node Schaefer parcellation) might have an impact on prediction results. Results highlighted that language abilities could be predicted to a similar degree from FC and SC data from both the 400‐node and 800‐node parcellations in the network‐specific approach in the total sample (800‐node: *R* ^2^ range = −0.24 to 0.05; 400‐node: *R* ^2^ range = −0.13 to 0.03; Tables S24 and S25). For nonverbal abilities, greater predictability could be observed in the 800‐node parcellation, particularly based on data from the FPN, for both FC and SC data compared to the 400‐node parcellation (800‐node: *R* ^2^ range = −0.24 to 0.34; 400‐node: *R* ^2^ range = 0.05–0.22; Tables S24 and S25). In this context, results, however, were also found to be more variable. Thus, these results support the general notion from the main analyses, particularly for verbal abilities, and extend it to another granularity. Beyond that, results further emphasized that the choice of parcellation may have an impact on the magnitude of the results. In the next step, we analyzed the impact of hemispheric specialization on prediction results by running separate ML analyses on connectivity data from the right and left hemispheres. Results suggested that connectivity data from the left and right hemispheres yield similar results across cognitive tests and samples (FC: Language abilities: Left hemisphere: *R* ^2^ range = −0.19 to 0.02, Right hemisphere: *R* ^2^ range = −0.26 to 0.05; Nonverbal abilities: Left hemisphere: *R* ^2^ range = −0.11 to 0.18, Right hemisphere: *R* ^2^ range = −0.20 to 0.17; SC: Language abilities: Left hemisphere: *R* ^2^ range = −0.21 to 0.05, Right hemisphere: *R* ^2^ range = −0.18 to 0.04; Nonverbal abilities: Left hemisphere: *R* ^2^ range = −0.12 to 0.23, Right hemisphere: *R* ^2^ range = −0.11 to 0.21; see Tables S26–S29). Furthermore, results were found to be comparable to the main analyses with regard to the *R* ^2^ (Nonverbal: *R* ^2^ range = −0.12 to 0.22; Verbal: *R* ^2^ range = −0.24 to 0.03). Thus, prediction results in the main analyses appeared to not be driven by one specific hemisphere, but instead, both hemispheres seemed to provide similar levels of information for the prediction. Finally, we examined the impact of eTIV on the prediction by running additional ML analyses controlling for it. Findings suggested similar levels of prediction performance to those in the main analyses across samples, cognitive tests, and input data (Controlled for eTIV: FC: Verbal: *R* ^2^ range = −0.24 to 0.03, Nonverbal: *R* ^2^ range = −0.09 to 0.15; SC: Verbal: *R* ^2^ range = −0.12 to 0.04, Nonverbal: *R* ^2^ range = −0.03 to 0.23; Not controlled for eTIV: FC: Verbal: *R* ^2^ range = −0.24 to 0.03, Nonverbal: *R* ^2^ range = −0.12 to 0.19; SC: Verbal: R^2^ range = −0.15 to 0.03, Nonverbal: *R* ^2^ range = −0.07 to 0.22; Tables S30 and S31). Thus, results emphasized that eTIV may only have a marginal impact on prediction results in the current study.

## 4 Discussion

The current study aimed at investigating the predictability of verbal abilities based on connectivity data [FC and SC data] from two domain‐general cognitive networks previously related to language performance [FPN and DMN] and the whole‐brain across the lifespan. Overall, results showed that language functions assessed with two distinct tests could not be reliably predicted from FC and SC data across feature sets and age groups in a large sample of individuals from the 1000BRAINS study. As such, no predictability differences emerged between distinct verbal tests based on currently employed brain features across the life span. In contrast to verbal functions, nonverbal cognitive abilities, that is, processing speed and visual working memory, could be moderately predicted from connectivity data, particularly SC, in the total and younger‐aged groups, but not in the older‐aged group across feature sets. Thus, our results highlight the overall challenges in predicting cognitive abilities in older adults.

In recent years, different cognitive abilities were found to be successfully predicted from connectivity data in younger and older adults (Dhamala et al. 2021; Dubois et al. 2018; Finn et al. 2015; Kwak et al. 2021; Li et al. 2020; Pläschke et al. 2020). Among these, there have also been promising findings for language abilities (Dhamala et al. 2021; Jiang et al. 2020a; Kwak et al. 2021; Rasero et al. 2021; Tomasi and Volkow 2020). Nevertheless, there are also initial findings showing that language abilities captured by a composite score may be predicted to a smaller extent or with lower accuracies from structural connectivity data compared to executive functions and global cognition across the lifespan (age range: 36–100 years; Feng et al. 2022). Results in the current study support these latter results and extend them to the prediction of language functions measured by two distinct tests capturing verbal knowledge and fluency from both FC and SC data in specific networks and the whole brain in younger and older adults from the 1000BRAINS study. One potential explanation for the limited predictability of verbal functions in the current study compared to early promising findings in the literature may pertain to the sample composition. Successful prediction results have been mainly reported for samples derived from the HCP (age range: 22–37 years) or for samples that also included cognitively impaired individuals (OASIS‐3; age range = 42–95 years; Dhamala et al. 2021; Jiang et al. 2020a; Kwak et al. 2021; Rasero et al. 2021; Tomasi and Volkow 2020). The current study included participants spanning an age range between 18 and 85 years from a population‐based cohort, 1000BRAINS, from Western Germany. As such, the samples used in prior studies may differ quite substantially from the currently employed lifespan sample. Additionally, prior studies have mostly used two different language scores as prediction targets, for example, picture vocabulary and oral reading recognition from the NIH toolbox, which may not exactly assess the same abilities as in the current analyses (Dhamala et al. 2021; Jiang et al. 2020a; Rasero et al. 2021; Tomasi and Volkow 2020). It may be the case that these abilities may be better predicted by connectivity data than the ones selected in the current study. Furthermore, it should be accentuated that most prior studies have relied on a whole‐brain approach instead of investigating specific networks for prediction.

Across analytic choices, language abilities could not be reliably predicted from connectivity data in the present study. This was the case for both single verbal test performance and composite verbal measures. Limited prediction results of the verbal composite measures might have been partially driven by an entanglement of fluid and crystallized abilities. Nevertheless, on their own, both fluid and crystallized verbal components similarly yielded limited prediction accuracies. In general, these findings pertained specifically to language functions and did not generalize to other cognitive functions, for example, processing speed and visual working memory, and demographic factors. As such, it may be argued that language functions as a cognitive domain appear to be challenging to predict from the currently chosen brain features in younger and older adults, leading to mixed results across the field, ML approaches, and samples. Results from the validation analyses aiming at extreme group classifications based on verbal functions further support this argument.

One potential explanation for the mixed results across the literature in terms of the predictability of language functions may relate to the potential influence of other factors on performance levels, for example, educational level, executive functions capabilities, and hormonal levels, obscuring the link between language functions and brain features, leading to a less pronounced encoding of language abilities in connectivity data and a stronger association with these other factors (Amunts et al. 2020; Fedorenko and Thompson‐Schill 2014; Griksiene and Ruksenas 2011; Guichet et al. 2024; Opdebeeck et al. 2016). The chosen brain connectivity features might not have carried sufficient information for discerning language performance differences in our sample, while other factors might potentially be more useful for prediction instead. Thus, it appears worthwhile to consider these other factors in future language prediction studies across the lifespan.

Another point to consider in this context is the operationalization of language functions across studies. Building upon one neuropsychological test for assessing verbal fluency and vocabulary knowledge, as done in this as well as various other studies (Cui et al. 2018; Kwak et al. 2021; Tomasi and Volkow 2020) might underestimate the complex and comprehensive nature of language abilities (Desai and Ricciardi 2021). Future research is needed to understand the predictive power of more extensive language paradigms tapping into the comprehension and production of, for example, whole sentences (Desai and Ricciardi 2021). Thus, it appears advisable to use a more diverse set of different neuropsychological tests or natural speech decodings in the future to more completely capture language functioning.

Verbal fluency and vocabulary knowledge have been suggested to follow different aging trajectories and to be associated with different brain regions and circuits (Hedden and Gabrieli 2004; Salthouse et al. 2003). This, in turn, may also hint at potential predictability differences between the two constructs based on imaging data. Against this initial assumption, no predictability differences emerged for vocabulary knowledge and production using brain features as input information in the current study. Instead, low prediction performance could be observed based on FC and SC data for both. Differences in predictability between vocabulary and verbal fluency surfaced only in the additional analyses using the demographic variables as input features. In this case, vocabulary could be successfully predicted from age, sex, and education (*R* ^2^ range = 0.18–0.23), while for verbal fluency a moderate level of prediction performance could only be partially reached (*R* ^2^ range = −0.04 to 0.13). As such, it appeared that verbal fluency is less influenced by age, sex, and education. Thus, it may be the case that other factors may explain a relevant portion of the variance in verbal fluency not considered in the current analyses. One potential candidate in this context may be executive functioning, which has been related to verbal fluency performance and has even been found to successfully predict verbal fluency scores (Amunts et al. 2020). Hence, it should be pointed out that verbal fluency and vocabulary knowledge not only seem to relate differently to brain parameters but also to nonbrain factors, which might influence prediction results.

Cognition may be viewed as a multidimensional construct composed of different domains (e.g., language being one) and specialized forms of processing. Given that cognitive functions are not a unitary concept and differentially relate to the brain, predictability differences between cognitive functions may emerge from imaging data (Harvey et al. 2019), which we also addressed in the current study. Results from the present investigation revealed that nonverbal cognitive functions could be moderately predicted across analytic choices compared to the poor prediction performance for language functions. Prediction performance, however, did not exceed 10% explained variance (*R* ^2^) (correlation between true and predicted scores: *r* < 0.28) for nonverbal cognitive functions in the younger and older groups in the current study. Only in the total sample was prediction performance for nonverbal functions found to be larger than *R* ^2^ > 0.1 (correlation between true and predicted scores: *r* < 0.47) for some modality, feature, and algorithm combinations. These results correspond to recent findings reported in the literature across different samples, particularly based on FC data (Avery et al. 2020; Ferguson et al. 2017; Greene et al. 2018; He et al. 2020; Heckner et al. 2023; Kraljević et al. 2024; Kwak et al. 2021). For example, processing speed, working memory, and fluid intelligence were predicted with a prediction accuracy (*r*) between 0.11 and 0.31 in younger and older adults from whole‐brain FC in multiple different samples across the life span (e.g., HCP cohort, OASIS‐3 cohort, Philadelphia Neurodevelopmental Cohort; Ferguson et al. 2017; He et al. 2020; Heckner et al. 2023; Kwak et al. 2021). Current results extend these prior findings to the modality of SC and emphasize that it remains unclear what amount of variance in cognition can actually be predicted from currently employed brain features (Easley et al. 2023; Genon et al. 2022; Schulz et al. 2022; Woo et al. 2017). Differences between samples, total versus younger and older groups, may be related to the larger sample size leading to an increase in signal and the addition of valuable variability from young to old that may result in clearer patterns of brain‐behavior relationships ultimately enhancing the accuracy of the predictive models. As such, it may be argued that nonverbal cognitive functions may be predicted to a greater extent from connectivity data than verbal functions in a large sample across the life span from the 1000BRAINS study, whereas achieving reliable and highly accurate predictions, particularly in older adults in the realm of cognition remains challenging.

Brain structure and function undergo manifold changes during the aging process, which are typically accompanied by age‐related cognitive decline (Hedden and Gabrieli 2004). Given that aging is a time of tremendous changes, the nature of brain‐behavior relationships may also be altered, leading to potential predictability differences in cognitive abilities between younger and older adults. Thus, we further investigated prediction performance in a younger and older age group in the current study. While no age group effects were encountered for language functions due to overall nonsuccessful prediction, differences between age groups emerged for the nonverbal cognitive functions, particularly based on SC data. In more detail, it was observed that 4% more variance could be explained in nonverbal cognitive measures in the younger (max. *R* ^2^ = 0.07) compared to the older group (max. *R* ^2^: 0.03). These results provide further support to prior studies hinting at higher predictability of specific cognitive functions in younger individuals and the challenging nature of individual‐level predictions in older adults (Kandaleft et al. 2022; Omidvarnia et al. 2024). For instance, Kandaleft et al. (2022) found that intelligence could be predicted in younger adults, but not in middle‐aged or older adults. Similarly, limited prediction power of different cognitive abilities (*r* < 0.25) based on rsfMRI parameters has been observed in a large sample from the UK Biobank (Omidvarnia et al. 2024). Hence, it may be reasoned that the higher inter‐individual variability in older adults may challenge concepts of a clear brain‐behavior relationship, leading to lower prediction performance. Nevertheless, it should also be mentioned in this context that there are some studies reporting better prediction of cognitive functions, for example, executive functions and working memory, in older compared to younger adults from connectivity data (Heckner et al. 2023; Pläschke et al. 2020). Differences in results between these findings and the current study could relate to differences in sample size. While both Heckner et al. (2023) and Pläschke et al. (2020) used relatively small samples, that is, *N* < 120, current analyses were based on data from *N* > 700 individuals allowing for a more realistic approximation of true predictability (Marek et al. 2022). Thus, it may be argued that cognition prediction may not perform equally well across the lifespan but may differ between younger and older age groups.

Specific cognitive functions may be related to processing in circumscribed brain networks. In this context, prior research has suggested that the FPN and DMN may be particularly related to cognitive control, working memory, attention, language abilities, and higher‐order cognition across the life span (Chenot et al. 2021; Smallwood et al. 2021; Zanto and Gazzaley 2013). Based on univariate findings showing links between specific behavioral constructs and networks in the literature, various prediction studies have taken up on this point and evaluated prediction performance differences arising from using brain features from specific relevant networks compared to those from the whole brain (Heckner et al. 2023; Nostro et al. 2018; Pläschke et al. 2020). Focusing on specific relevant features might also reduce the potential drawbacks of the curse of dimensionality, where a high number of data dimensions in relation to a lower number of datapoints can lead to reduced model performance. Following this and the initially partly promising univariate results in the current study, we here investigated prediction performance from connectivity information in two networks [DMN and FPN] that have been frequently associated with a diverse set of cognitive functions and compared it to a whole brain approach (Mwangi et al. 2014). For those ML models with moderate prediction performance, it appeared that network‐specific models did not outperform the whole‐brain approach in the current setup. Even a slight advantage for the whole brain approach could be observed across algorithms, modalities, and targets (network‐specific: max *R* ^2^ = 0.20; whole brain: max *R* ^2^ = 0.22), which is in line with recent findings showing that whole brain information led to better cognitive prediction results than network‐specific FC (Heckner et al. 2023). Thus, it may be argued that a single network perspective may leave out relevant information that may be available from the whole brain or the network perspective is not specific enough to effectively boost prediction performance. Hence, it may be advisable in prospective studies to compare different approaches to choose the one that best describes the specific data. Interestingly, the here investigated network‐specific information showed differential capability of predicting specific cognitive functions: While nonverbal cognitive performances were to a certain degree predictable, language abilities were not, even though we selected these networks as they have been frequently associated with a diverse set of cognitive functions. The current results, however, rather point to a stronger association with nonverbal than verbal cognitive functions across the two hemispheres.

ML performance may be impacted by different factors, including confounding variables, for example, age, sex, and education. To assess their contribution to the current results, we performed additional confounder analyses and used demographic factors as input features to ML (Dadi et al. 2021; Krämer et al. 2024; Rasero et al. 2021). Analyses were performed based on these features alone and in conjunction with brain features. Results highlighted a general trend for increases in prediction accuracy (*R* ^2^) for models based on demographic variables alone and jointly with brain features. This is in line with prior studies showing significant improvements in prediction performance when demographic variables were included in ML models (Dadi et al. 2021; Rasero et al. 2021; Vieira et al. 2022). In this context, it should be accentuated that adding brain features to the demographic factors did not further improve ML performance, which corresponds to prior findings in the 1000BRAINS study and the UK Biobank and extends it to different cognitive targets and age groups (Krämer et al. 2024; Omidvarnia et al. 2024). Thus, it appeared that brain features only explained a small amount of variability in the data and were clearly outperformed by demographic data. It should, nevertheless, be pointed out that differences emerged across cognitive variables, input data, and samples, even if these effects were small. Furthermore, attention should be drawn to the fact that demographic factors improved prediction accuracies to different degrees across cognitive targets. The main exception arose for verbal fluency, which could only be predicted with a maximum accuracy of *R* ^2^ = 0.13 from demographic factors compared to up to *R* ^2^ = 0.39 for visual working memory. Thus, it appears that verbal fluency may be more strongly linked to other factors not part of this analysis. Simultaneously, it may be argued that age, sex, and education may have a substantial influence on ML prediction performance in younger and older adults; however, not across all cognitive targets. Hence, to improve the prediction performance of cognitive outcomes, it appears fruitful to consider confounding variables like demographic factors in ML models across the lifespan and to examine their relation to different cognitive targets also using other cohort data.

### 4.1 Limitations and Methodological Considerations

The current study concentrated on the prediction of verbal cognitive functions based on SC and FC data. Overall, prediction performance across all verbal and nonverbal cognitive measures remained fairly limited. In future studies, it, thus, might be advisable to include other input modalities, for example, task‐based or dynamic functional connectivity (Jiang et al. 2020b), or to use a multimodal approach (Niu et al. 2020; Rasero et al. 2021; Vieira et al. 2022). Furthermore, as it remains open to what extent brain data may be helpful in the prediction of cognitive measures, other nonimaging data may be added to prediction models to boost performance and disentangle the individual contributions of each factor (Murdaca et al. 2021). In this realm, it may also become relevant to include additional steps in the ML pipeline beyond the embedded feature selection in Elastic Net regression to reduce the feature space and, with it, the potential issues arising due to high dimensionality. It might be particularly advisable to compare prediction performance across a range of different dimensionality reduction techniques, such as principal component analysis (PCA), and filter and wrapper techniques, to select the most meaningful features for prediction (Jollans et al. 2019).

Another methodological aspect to consider is the choice of parcellation, which has been found to exert an influence on ML prediction performance (Dhamala et al. 2021, 2023; Mellema et al. 2022). In the current study, we opted for the functionally derived 400‐node Schaefer parcellation for both FC and SC, as it allows comparability across modalities and has been frequently employed in ML cognition prediction studies and studies across the life span (Schaefer et al. 2018; Yeo et al. 2011). To assess the impact of parcellation resolution, we further assessed the impact of using connectivity data derived from the 800‐node Schaefer parcellation in the prediction. In the direct comparison to the 400‐node parcellation, results were found to show only small differences, particularly for verbal abilities. Nevertheless, it should be pointed out that subcortical and cerebellar regions, which have been shown to be relevant for cognitive processing, have not been included in the current study and thus could be considered in future studies (Jobson et al. 2024; Turker et al. 2023; Weis et al. 2020). Furthermore, as pointed out above, it would be desirable for future studies to investigate different operationalizations of language functions to tackle their complex and comprehensive nature (Desai and Ricciardi 2021), as well as examine their retest reliability to exclude state effects (Gell et al. 2024).

## 5 Conclusions

In the current study, we investigated if language abilities, that is, verbal fluency and vocabulary knowledge, may be predicted based on structural and functional connectivity data in the FPN, DMN, and the whole brain across the lifespan using data from the 1000BRAINS cohort. We found that neither vocabulary knowledge nor verbal fluency could be successfully predicted in the current sample. This result was found to be consistent across modalities [FC and SC], feature sets [DMN, FPN, DMN‐FPN, whole brain], algorithms [EN and SVR], and age [total, younger, and older aged] groups. Low prediction results were found to be circumscribed to language functions, as other nonverbal functions could be moderately predicted, particularly in the total and younger age group. Thus, the findings underscore the distinct role of language abilities among cognitive functions and suggest that factors beyond brain connectivity may significantly influence differences in language performance. To sum up, our results stressed that verbal and nonverbal cognitive functions may differ in their predictability from specific brain network patterns across the life span. This may be due to the networks chosen, the reliance on other factors interfering with that of brain patterns, as well as different processing streams in verbal and nonverbal cognitive functions. Prospective studies should further investigate this and expand on results in younger and older adults.

## Ethics Statement

This study was performed in line with the principles of the Declaration of Helsinki. Approval was granted by the Ethics Committee of the University of Duisburg‐Essen, Germany (Heinz‐Nixdorf‐Recall study: No. 11‐4678; Multi‐Generation‐Study: No. 12‐5199‐BO).

## Consent

The study protocol was approved by the ethics committee of the University of Duisburg‐Essen and all participants provided written informed consent prior to inclusion. The study procedures comply with the Declaration of Helsinki.

## Conflicts of Interest

The authors declare no conflicts of interest.

## Supporting information


**Data S1** Supporting Information.

## Data Availability

Due to local regulations of data acquisition and usage, data of 1000BRAINS are available upon request from the responsible PI.
