# Supplementary material for: Prediction of Verbal Abilities From Brain Connectivity Data Across the Lifespan Using a Machine Learning Approach
Source: Hum Brain Mapp. 2025 Mar 25;46(5):e70191. doi: 10.1002/hbm.70191 (PMC11933761; doi:10.1002/hbm.70191)
Supplement: Supplementary file 1 — Data S1 Supporting Information. [file HBM-46-e70191-s001.docx]

**Supplement**

**Prediction of language comprehension and production from brain connectivity data across the life span using a ML approach**

Deborah Früh^1,2^, Camilla Mendl-Heinisch^1,2^, Nora Bittner^1,2^, Susanne Weis^3,4^ & Svenja Caspers^1,2^

Institutions:

^1^Institute of Neuroscience and Medicine (INM-1), Research Centre Jülich, Jülich, Germany

^2^Institute for Anatomy I, Medical Faculty & University Hospital Düsseldorf, Heinrich Heine University Düsseldorf, Düsseldorf, Germany

^3^Institute of Neuroscience and Medicine, Brain and Behaviour (INM-7), Research Centre Jülich, Jülich, Germany

^4^Institute of Systems Neuroscience, Medical Faculty, Heinrich Heine University Düsseldorf, Düsseldorf, Germany

Email addresses of authors*:* d.frueh@hhu.de, c.mendl-heinisch@fz-juelich.de, n.bittner@fz-juelich.de, s.weis@fz-juelich.de, s.caspers@fz-juelich.de

**Abbreviations**

| Alg. | Algorithm |
| --- | --- |
| Acc. | Accuracy |
| B | Brain data |
| B+D | Brain data + demographics |
| DMN | Default mode network |
| EN | Elastic Net Regression |
| eTIV | Estimated total intracranial volume |
| FPN | Frontoparietal networks |
| FPNDMN | Combined FPN and DMN |
| FSet | Feature set |
| M | Mean |
| Mod. | Modality |
| MAE | Mean Absolute Error |
| PF | Phonematic fluency |
| r | Pearson’s correlation coefficient |
| R^2^ | Coefficient of determination |
| Ridge | Ridge Classifier |
| SF | Semantic fluency |
| SD | Standard deviation |
| SVC | Support Vector Classifier |
| SVR | Support Vector Regression |
| Tar. | Target |
| TMT-A | Trail Making Test - A |
| VER | Combined verbal functions |
| VF | Combined verbal fluency |
| VOC | Vocabulary |
| vWM | Visual working memory |
| WHOLE | Whole brain data |

**Supplementary Methods**

*Supplementary Figure 1.* Display of FPN (orange) and DMN (red) nodes in the left and right hemisphere from the 400-node Schaefer parcellation (7 Networks) used in the ML prediction analyses. Plots were generated using NetPlotBrain and Nilearn, which utilize different python packages (Abraham et al., 2014; Fanton & Thompson, 2023).


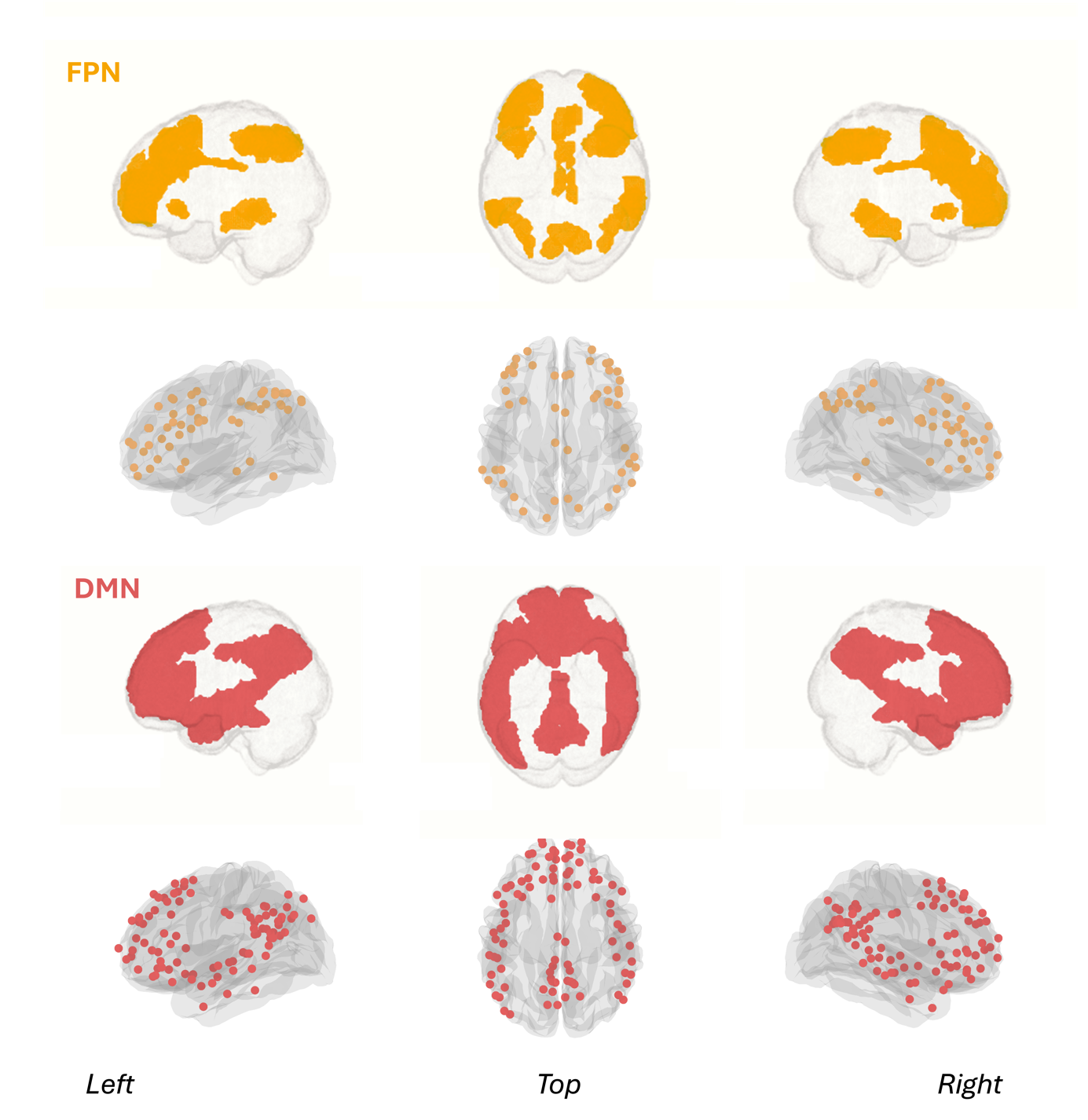


*Supplementary Table 1.* Original labels, centroid coordinates and corresponding anatomical labels of FPN nodes in the left and right hemisphere from the 400-node Schaefer parcellation (7 Networks) used in the ML prediction analyses (Schaefer et al., 2018).


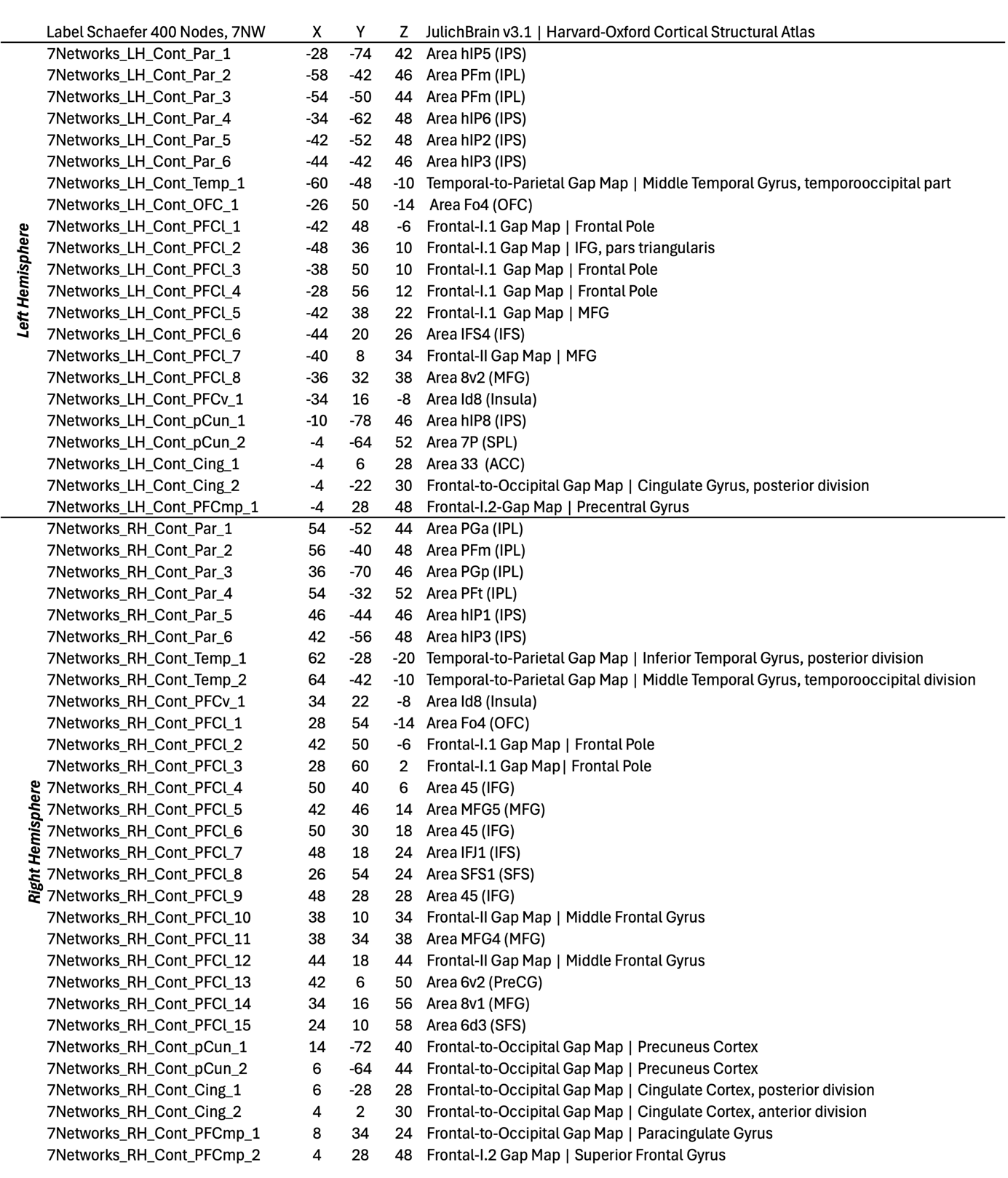


*Supplementary Table 2.* Original labels, centroid coordinates and corresponding anatomical labels of DMN nodes in the left hemisphere from the 400-node Schaefer parcellation (7 Networks) used in the ML prediction analyses (Schaefer et al., 2018).


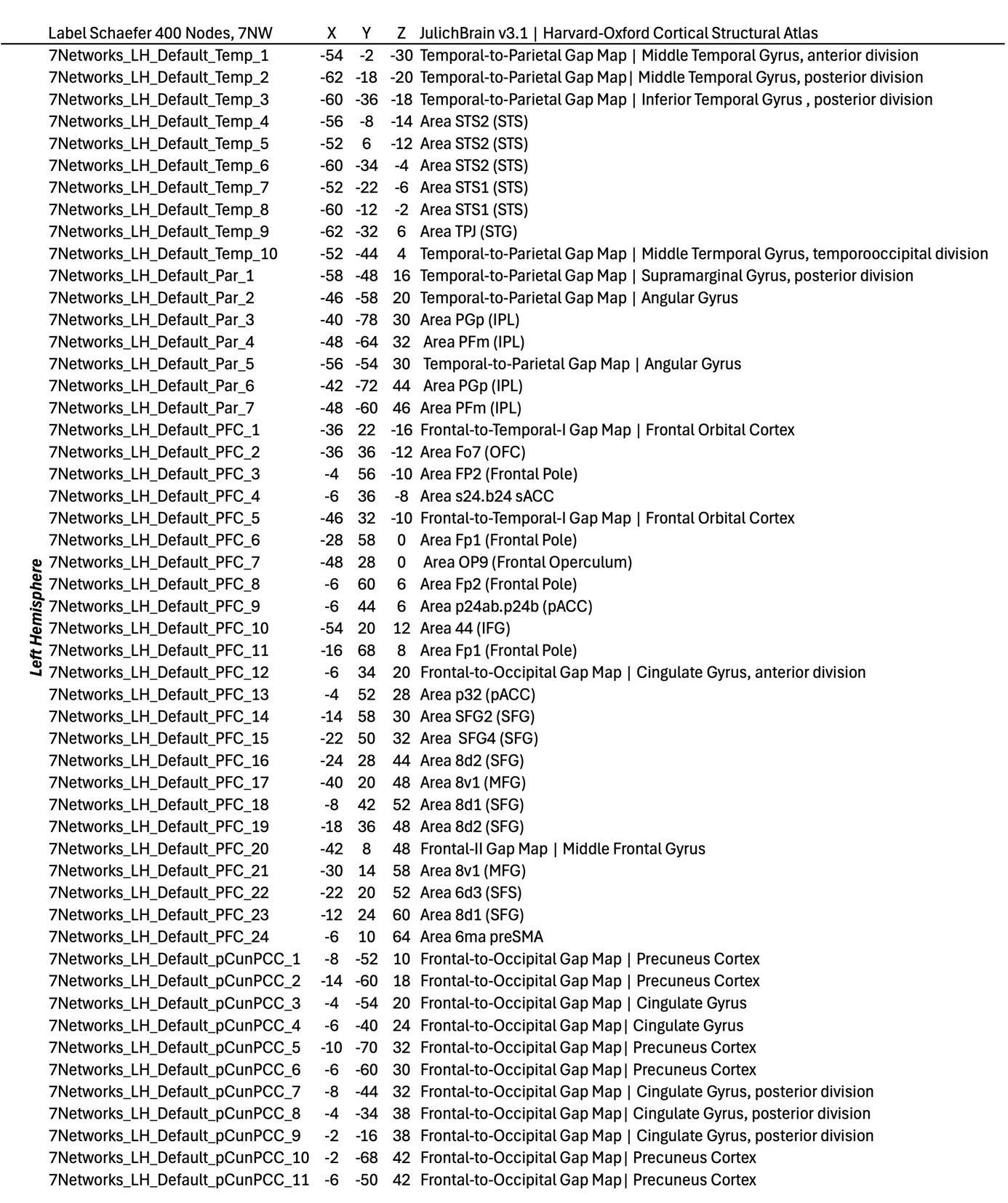


*Supplementary Table 3.* Original Labels, centroid coordinates and corresponding anatomical labels of DMN nodes in the right hemisphere from the 400-node Schaefer parcellation (7 Networks) used in the ML prediction analyses (Schaefer et al., 2018).


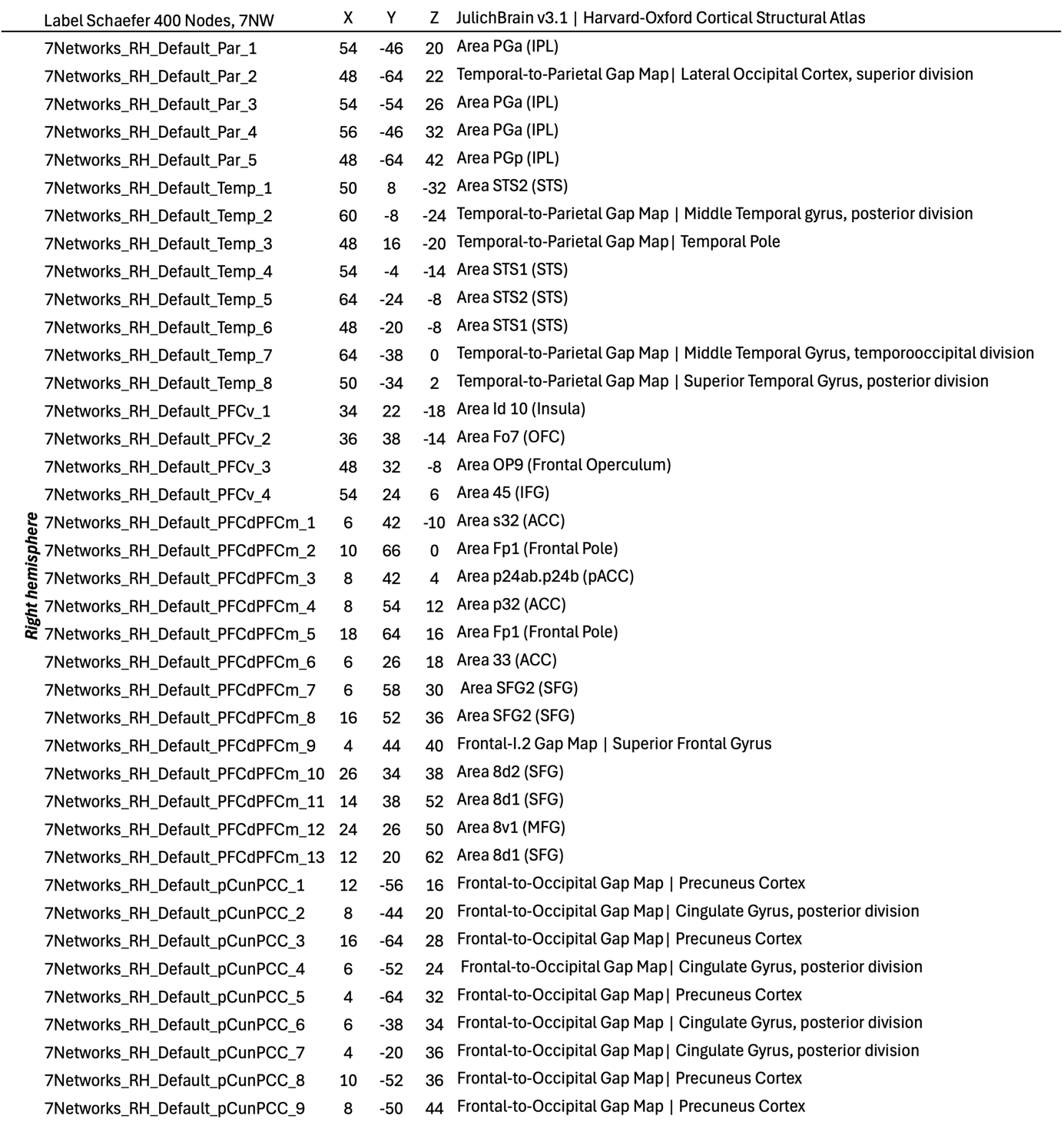


*Supplementary Table 4.* Demographic information of extreme cognitive groups (25% lowest and highest scorers) in the younger, older and total sample.

|  |  | Low | | | | High | | | |
| --- | --- | --- | --- | --- | --- | --- | --- | --- | --- |
| Sample | Test | N | Age | Edu | Score | N | Age | Edu | Score |
| Total | SF | 179  (106 M, 73 F) | 63.6 (12.9) | 6.1  (1.8) | -1.3  (0.5) | 179  (99 M, 80 F) | 54.3 (13.8) | 7.5  (1.8) | 1.3 (0.33) |
|  | PF | 179  (106 M, 73 F) | 61.9 (12.4) | 6.3 (1.9) | -1.2 (0.4) | 179  (89 M, 90 F) | 56.4 (12.5) | 7.5 (1.9) | 1.3 (0.5) |
|  | VF | 179  (108 M, 71 F) | 63.4 (11.9) | 6.2 (1.8) | -1.1 (0.4) | 179  (102 M, 77 F) | 54.0 (12.8) | 7.6 (1.7) | 1.1 (0.4) |
|  | VOC | 179  (87 M, 92 F) | 61.4 (14.2) | 5.8 (1.6) | -1.3 (0.6) | 179  (107 M, 72 F) | 59.7 (12.3) | 8.1 (1.6) | 1.2 (0.4) |
|  | VER | 179  (99 M, 80 F) | 63.6 (11.7) | 5.8 (1.6) | -1.0 (0.4) | 179  (101 M, 78 F) | 56.7 (12.5) | 7.9 (1.7) | 1.0 (0.3) |
| Older | SF | 102  (60 M, 42 F) | 70.0 (5.6) | 5.8 (1.8) | -1.2  ( 0.4) | 102  (53 M, 49 F) | 67.1 (5.2) | 7.3 (1.9) | 1.3 (0.6) |
|  | PF | 102  (64 M, 38 F) | 68.9 (5.3) | 5.9 (1.7) | -1.2 (0.4) | 102  (45 M, 57 F) | 66.9 (5.2) | 7.3 (2.0) | 1.3 (0.6) |
|  | VF | 102  (64 M, 38 F) | 69.9 (5.6) | 5.9 (1.8) | -1.1 (0.4) | 102  (52 M, 50 F) | 66.8 (5.0) | 7.5 (1.9) | 1.1 (0.5) |
|  | VOC | 102  (51 M, 51 F) | 69.5 (5.8) | 5.6 (1.6) | -1.3 (0.6) | 102  (56 M, 46 F) | 68.1 (5.5) | 8.1 (1.7) | 1.2 (0.4) |
|  | VER | 102  (62 M, 40 F) | 69.6 (5.6) | 5.6 (1.5) | -1.0 (0.4) | 102  (53 M, 49 F) | 67.2 (5.3) | 7.7 (1.9) | 1.0 (0.3) |
| Younger | SF | 78  (42 M, 36 F) | 47.6 (10.6) | 6.9 (1.7) | -1.2 (0.5) | 78  (48 M, 30 F) | 46.0 (10.8) | 7.8 (1.6) | 1.3 (0.5) |
|  | PF | 78  (39 M, 39 F) | 47.7 (10.5) | 7.1 (1.8) | -1.3 (0.5) | 78  (48 M, 30 F) | 47.3 (10.0) | 7.6 (1.7) | 1.3 (0.5) |
|  | VF | 78  (39 M, 39 F) | 48.0 (10.5) | 6.8 8( 1.7) | -1.1 (0.4) | 78  (48 M, 30 F) | 47.0 (10.0) | 7.9 (1.6) | 1.1 (0.4) |
|  | VOC | 78  (35 M, 43 F) | 45.6 (11.6) | 6.2 (1.5) | -1.4 (0.7) | 78  (47 M, 31 F) | 49.0 (9.7) | 8.1 (1.5) | 1.2 (0.3) |
|  | VER | 78  (34 M, 44 F) | 47.5 (10.6) | 6.6 (1.7) | -1.0 (0.5) | 78  (50 M, 28 F) | 48.1 (9.7) | 8.1 (1.5) | 0.9 (0.3) |

*Note.* Edu = educational level measured by ISCED97, M= Males, F = Females. Mean values displayed with standard deviations appearing in parentheses.

**Supplementary Results**

*Supplementary Table 5.* Pearson’s correlation between FC & SC network parameters, cognitive tests and age in the total sample.

|  | Age | SF | PF | Vocab | TMT-A | VF | Verbal | vWM |
| --- | --- | --- | --- | --- | --- | --- | --- | --- |
| FC FPN W | -0.04 | 0.10** | 0.07 | 0.07 | 0.09* | 0.10** | 0.11** | 0.07 |
| FC DMN W | -0.07 | 0.01 | 0.00 | 0.00 | 0.03 | 0.01 | 0.01 | 0.06 |
| FC FPN-DMN | -0.01 | 0.03 | 0.06 | 0.04 | 0.04 | 0.05 | 0.05 | 0.04 |
| SC FPN W | -0.46*** | 0.15*** | 0.08* | 0.04 | 0.26*** | 0.14*** | 0.12** | 0.25*** |
| SC DMN W | -0.31*** | 0.14*** | 0.04 | 0.08* | 0.16*** | 0.11** | 0.11** | 0.21*** |
| SC FPN-DMN | -0.42*** | 0.16*** | 0.08* | 0.08* | 0.24*** | 0.14*** | 0.14*** | 0.25*** |
| Age | - | -0.25*** | -0.14*** | -0.06 | -0.59*** | -0.23*** | -0.20*** | -0.58*** |

*Note*. *p<0.05, **p<0.01, **p<0.001, W = Within-network connectivity.


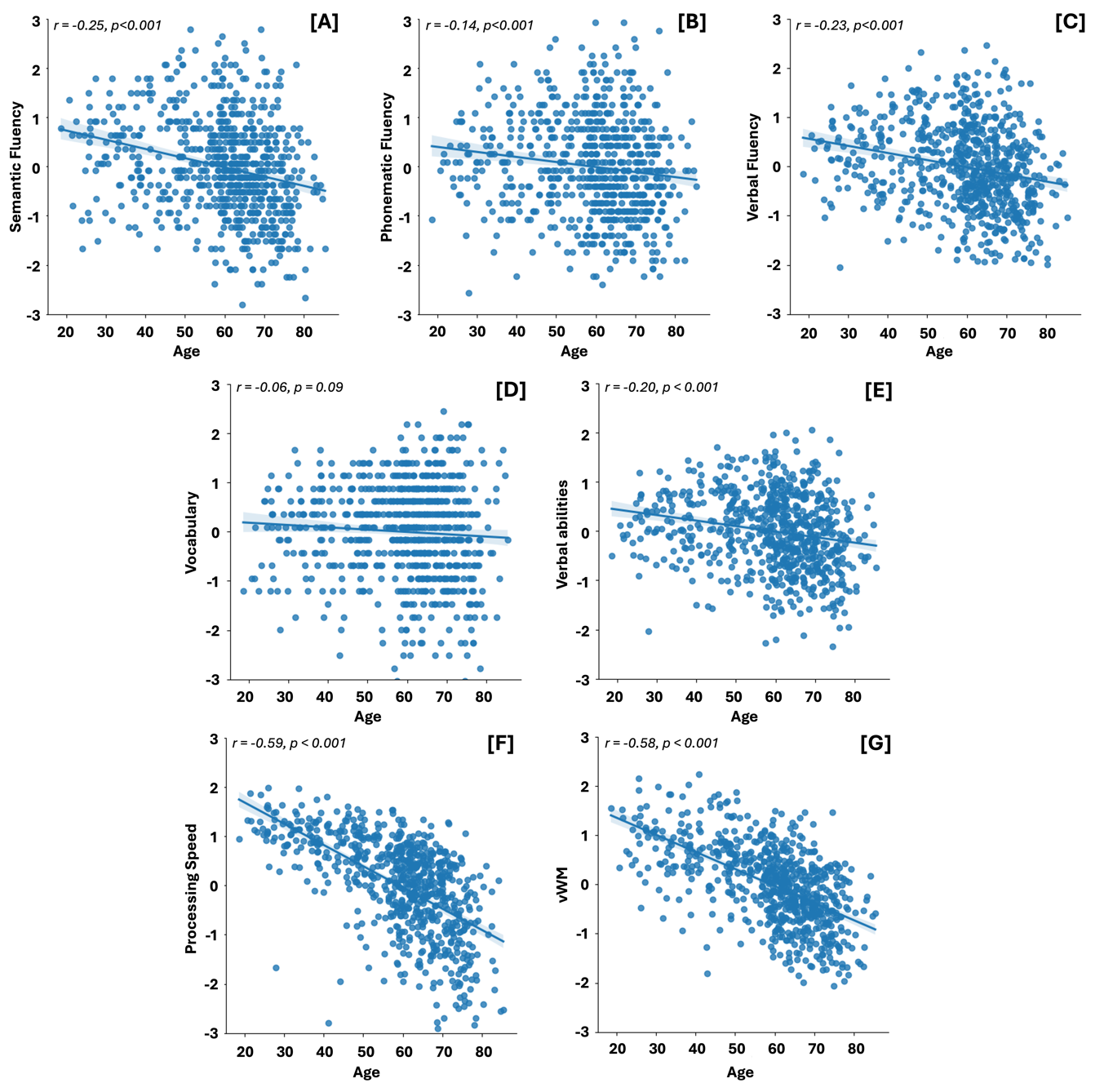
*Supplementary Figure 2.* Pearson’s correlation between cognitive tests [A-G] and age in the total sample.

*Supplementary Table 6.* Independent samples t-test results assessing differences in cognitive performance between younger and older participants in the total sample.

|  | Younger | | Older | |  | *df* | *t* | p-value |
| --- | --- | --- | --- | --- | --- | --- | --- | --- |
|  | *M* | *SD* | *M* | *SD* |  |  |  |  |
| Semantic Fluency | 27.6 | 6.7 | 24.0 | 6.8 |  | 675 | -6.94 | p < 0.001 |
| Phonematic Fluency | 20.4 | 5.9 | 18.7 | 6.0 |  | 671 | -3.66 | p < 0.001 |
| Verbal Fluency | 0.22 | 0.82 | -0.17 | 0.85 |  | 677 | -6.2 | p < 0.001 |
| Vocabulary | 32.0 | 3.5 | 31.4 | 4.0 |  | 703 | -2.2 | p < 0.05 |
| Verbal abilities | 0.18 | 0.74 | -0.14 | 0.78 |  | 685 | -5.5 | p < 0.001 |
| Processing Speed | 28.0 | 9.3 | 39.4 | 11.2 |  | 710 | -14.9 | p < 0.001 |
| Corsi | 5.4 | 1.1 | 4.7 | 1.0 |  | 632 | -9.1 | p < 0.001 |
| Benton | 9.3 | 6.2 | 16.5 | 7.5 |  | 710 | -14.1 | p < 0.001 |
| VPT | 9.7 | 1.9 | 7.7 | 1.7 |  | 628 | -14.3 | p < 0.001 |
| vWM | 0.48 | 0.8 | -0.37 | 0.69 |  | 663 | -16.1 | p < 0.001 |

*Note*. For individual cognitive tests raw scores are displayed. For composite scores standardized values are displayed.

*Supplementary Table 7.* Independent samples t-test results assessing differences in brain network parameters (FC & SC) between younger and older participants in the total sample.

|  | Younger | | Older | |  | *df* | *t* | p-value |
| --- | --- | --- | --- | --- | --- | --- | --- | --- |
|  | *M* | *SD* | *M* | *SD* |  |  |  |  |
| FC DMN W | 0.35 | 0.04 | 0.34 | 0.05 |  | 715 | 0.86 | 0.39 |
| FC FPN W | 0.34 | 0.05 | 0.33 | 0.05 |  | 715 | 1.34 | 0.18 |
| FC FPN-DMN | 0.30 | 0.04 | 0.30 | 0.04 |  | 715 | 0.49 | 0.63 |
| SC DMN W | 0.83 | 0.06 | 0.79 | 0.08 |  | 715 | 7.7 | p < 0.001 |
| SC FPN W | 0.96 | 0.10 | 0.87 | 0.12 |  | 715 | 10.6 | p < 0.001 |
| SC FPN-DMN | 0.80 | 0.07 | 0.73 | 0.09 |  | 715 | 10.0 | p < 0.001 |

*Note*. W = Within-network connectivity.

*Supplementary Figure 3.* Significant Pearson’s correlation between FC network parameters [A-D] and cognitive tests in the total sample.

**
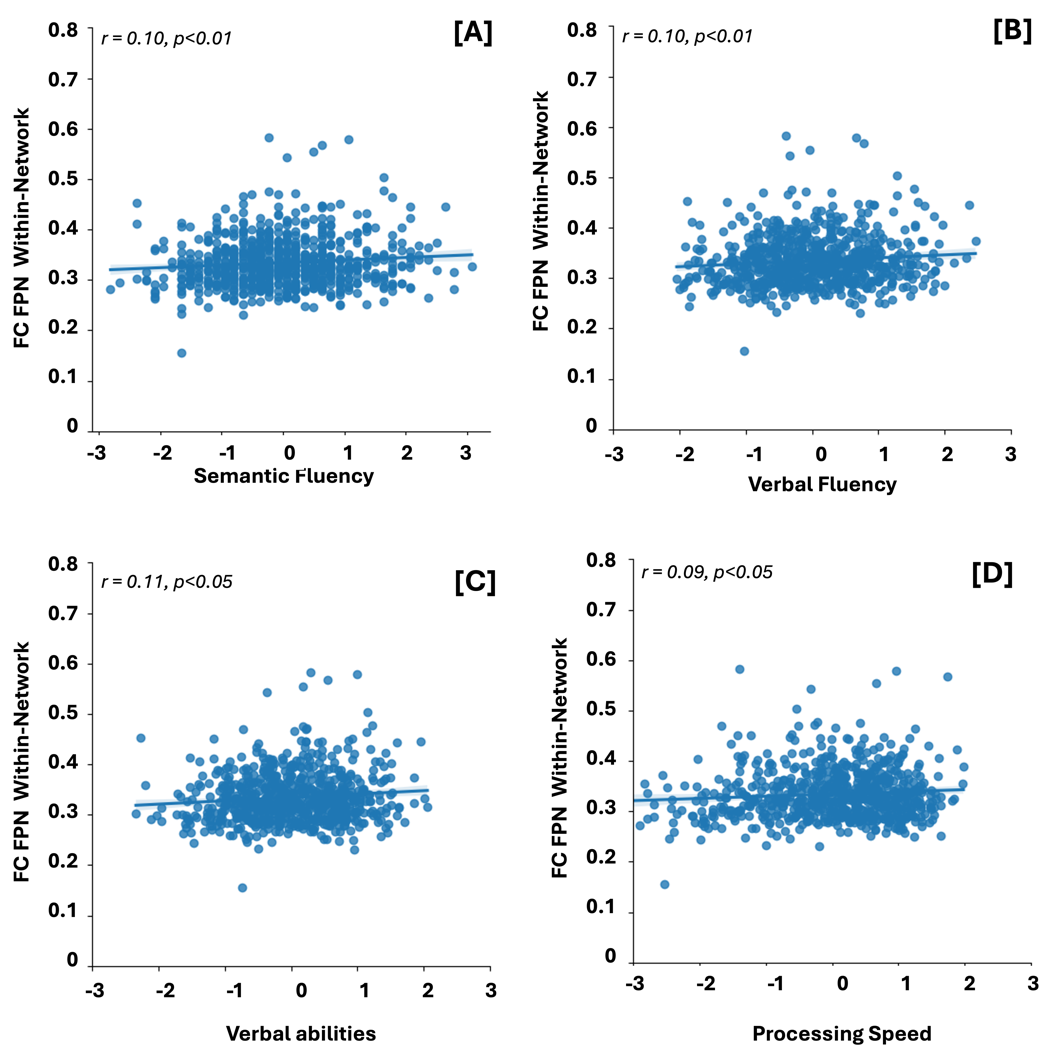
**


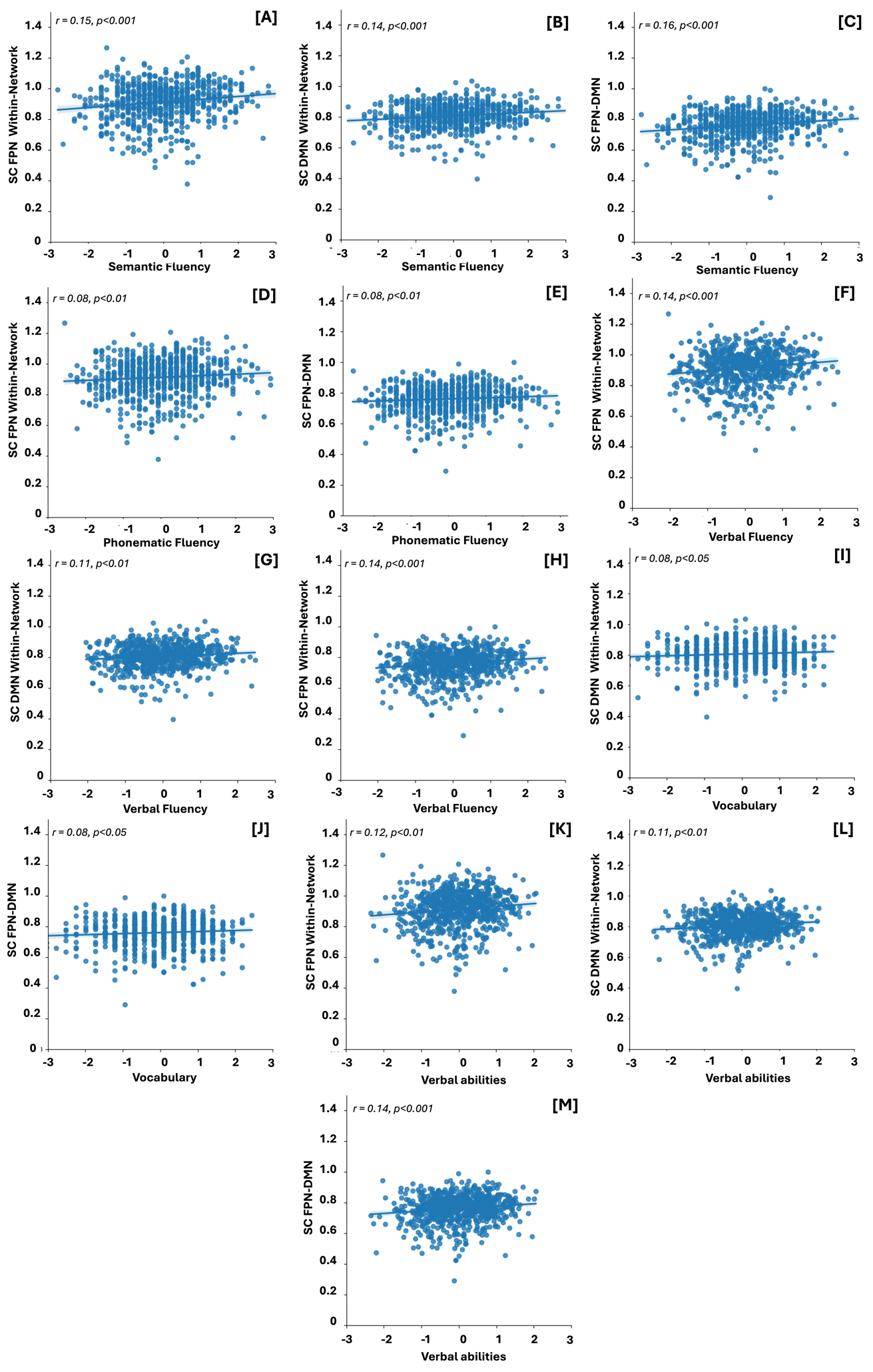
*Supplementary Figure 4.* Significant Pearson’s correlation between SC network parameters [A-M] and verbal cognitive tests in the total sample.

**
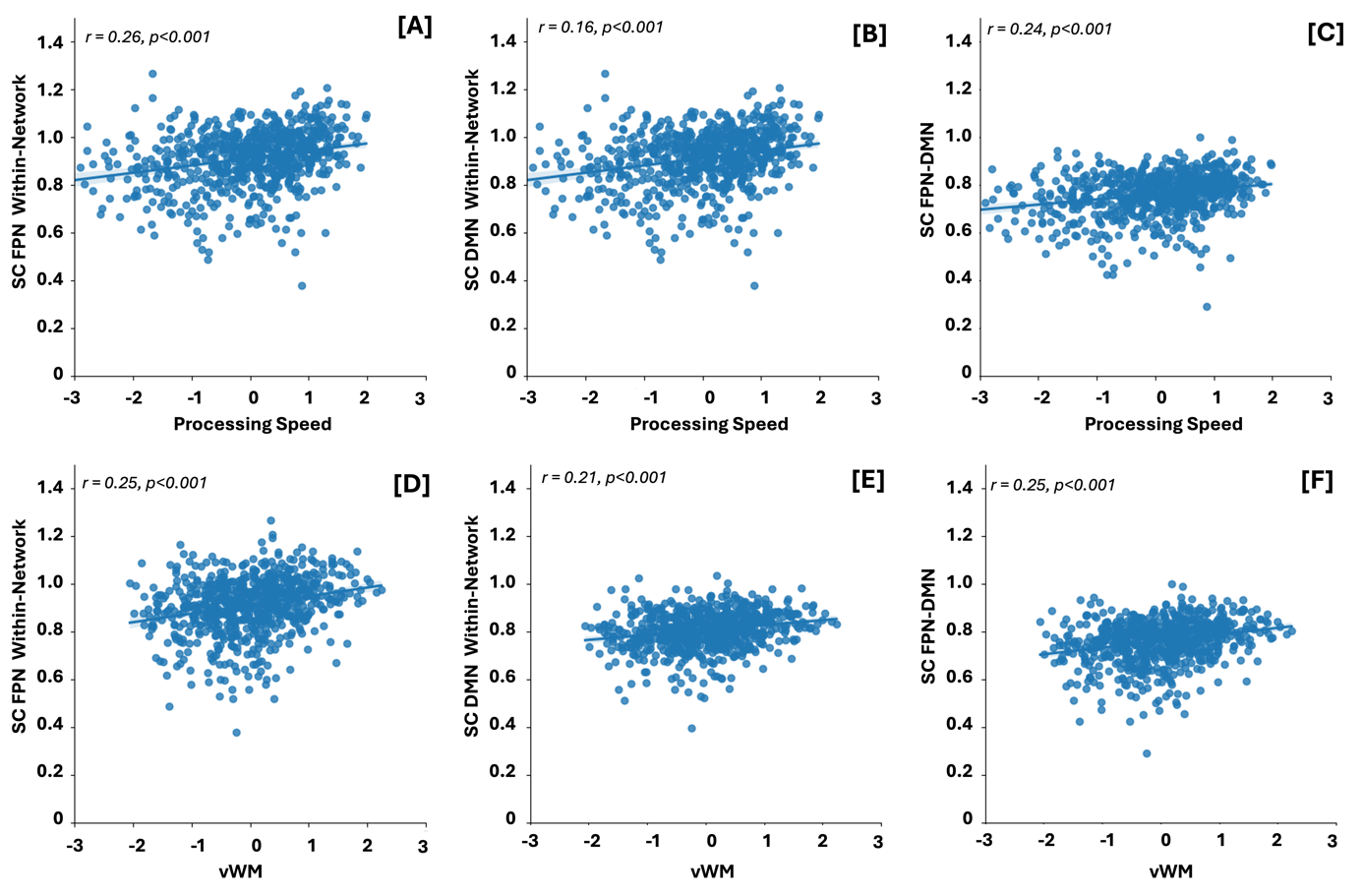
***Supplementary Figure 5.* Significant Pearson’s correlation between SC network parameters [A-F] and non-verbal cognitive tests in the total sample.


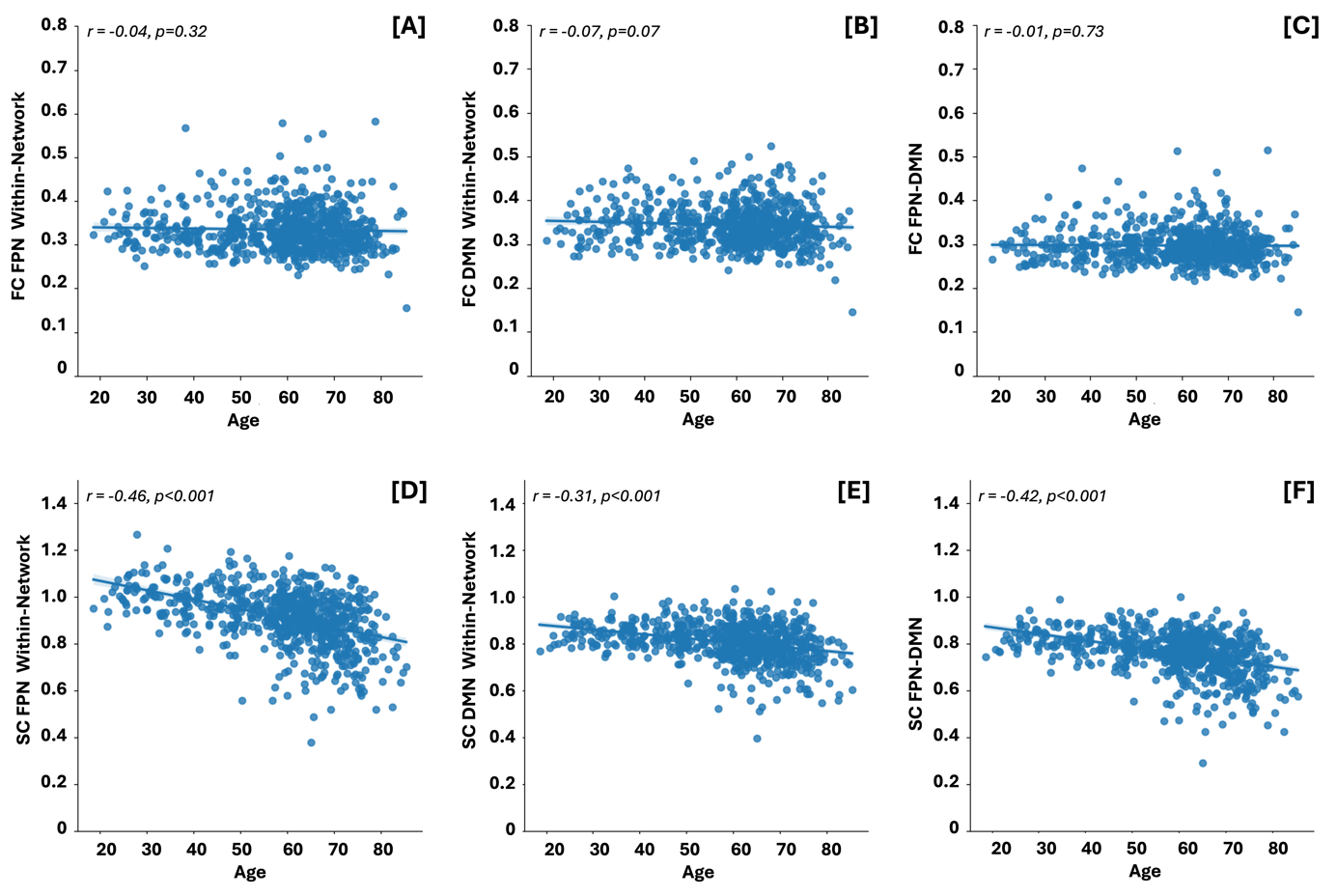
*Supplementary Figure 6.* Pearson’s correlation between FC & SC network parameters [A-F] and age in the total sample.

***ML Prediction Results***

*Supplementary Table 8.* Prediction results (Coefficient of Determination [R^2^]) for verbal and non-verbal cognitive functions from FC across samples, i.e. total, older and younger.

| FC |  | Total | | Older | | Younger | |
| --- | --- | --- | --- | --- | --- | --- | --- |
| Mod. | Tar. | EN | SVR | EN | SVR | EN | SVR |
| DMN | SF | 0.02  (0.03) | 0.00  (0.05) | -0.03  (0.02) | -0.05  (0.05) | -0.03  (0.04) | -0.03  (0.07) |
|  | PF | 0.00  (0.03) | -0.02  (0.05) | -0.01  (0.02) | -0.03  (0.05) | -0.04  (0.05) | -0.09  (0.08) |
|  | VF | 0.02  (0.03) | -0.01  (0.06) | -0.01  (0.04) | -0.01  (0.04) | -0.05  (0.05) | -0.09  (0.07) |
|  | VOC | -0.01  (0.03) | -0.03  (0.04) | -0.01  (0.04) | -0.02  (0.07) | -0.04  (0.09) | -0.09  (0.06) |
|  | VER | 0.03  (0.04) | -0.01  (0.07) | 0.02  (0.04) | 0.00  (0.05) | -0.02  (0.03) | -0.13  (0.07) |
|  | TMT-A | 0.08  (0.05) | 0.05  (0.06) | -0.02  (0.04) | -0.04  (0.06) | -0.02  (0.07) | -0.04  (0.07) |
|  | vWM | 0.15  (0.06) | 0.13  (0.08) | 0.00  (0.03) | -0.03  (0.07) | 0.02  (0.07) | 0.03  (0.08) |
| FPN | SF | 0.01  (0.03) | 0.00  (0.04) | -0.01  (0.03) | -0.01  (0.04) | -0.03  (0.03) | -0.03  (0.04) |
|  | PF | -0.01  (0.02) | 0.00  (0.03) | -0.01  (0.05) | -0.03  (0.05) | -0.03  (0.03) | -0.05 (0.06) |
|  | VF | 0.01  (0.03) | 0.02 (0.04) | 0.00  (0.03) | -0.02  (0.05) | -0.03  (0.02) | -0.05  (0.04) |
|  | VOC | -0.01  (0.02) | -0.01  (0.04) | -0.01  (0.03) | -0.01  (0.04) | -0.05  (0.06) | -0.05  (0.07) |
|  | VER | 0.00  (0.03) | 0.00  (0.03) | -0.01  (0.06) | -0.02  (0.06) | -0.03  (0.02) | -0.05  (0.05) |
|  | TMT-A | 0.09  (0.05) | 0.07  (0.06) | 0.00  (0.03) | -0.02  (0.05) | -0.03  (0.04) | -0.09  (0.06) |
|  | vWM | 0.07  (0.05) | 0.05  (0.04) | 0.03  (0.05) | 0.02  (0.07) | -0.04  (0.05) | -0.03  (0.05) |
| FPNDMN | SF | 0.03  (0.03) | -0.08  (0.06) | -0.02  (0.02) | -0.11  (0.06) | -0.04  (0.04) | -0.12  (0.08) |
|  | PF | 0.00  (0.03) | -0.07  (0.06) | -0.02  (0.03) | -0.07  (0.08) | -0.02  (0.03) | -0.17  (0.09) |
|  | VF | 0.03  (0.03) | -0.07  (0.06) | -0.02  (0.03) | -0.08  (0.09) | -0.03  (0.03) | -0.19  (0.10) |
|  | VOC | 0.00  (0.03) | -0.07  (0.06) | -0.03  (0.06) | -0.09  (0.10) | -0.03  (0.04) | -0.13  (0.08) |
|  | VER | 0.03  (0.03) | -0.06  (0.07) | -0.01  (0.04) | -0.06  (0.09) | -0.03  (0.03) | -0.24  (0.09) |
|  | TMT-A | 0.10  (0.05) | 0.08  (0.06) | -0.02  (0.04) | -0.12  (0.08) | -0.02  (0.04) | -0.06  (0.07) |
|  | vWM | 0.16  (0.05) | 0.12  (0.07) | 0.00  (0.06) | -0.08  (0.09) | 0.01  (0.07) | 0.01  (0.07) |
| WHOLE | SF | 0.02  (0.05) | -0.09  (0.08) | -0.03  (0.05) | -0.10  (0.12) | -0.03  (0.05) | -0.13  (0.10) |
|  | PF | -0.03  (0.04) | -0.13  (0.08) | -0.05  (0.06) | -0.07  (0.09) | -0.04  (0.05) | -0.21  (0.10) |
|  | VF | 0.00  (0.04) | -0.09  (0.08) | -0.04  (0.07) | -0.05  (0.08) | -0.04  (0.05) | -0.16  (0.08) |
|  | VOC | -0.03  (0.03) | -0.14  (0.07) | -0.03  (0.04) | -0.14  (0.08) | -0.03  (0.05) | -0.13  (0.09) |
|  | VER | 0.01  (0.03) | -0.07  (0.07) | -0.01  (0.06) | -0.04  (0.08) | -0.03  (0.04) | -0.14  (0.07) |
|  | TMT-A | 0.14  (0.04) | 0.10  (0.06) | -0.01  (0.02) | -0.10  (0.08) | 0.00  (0.08) | 0.01  (0.09) |
|  | vWM | 0.19  (0.07) | 0.17  (0.07) | -0.01  (0.08) | -0.11  (0.12) | 0.00  (0.09) | 0.03  (0.08) |

*Note.* Standard deviation (SD) appears in parentheses.

*Supplementary Table 9.* Prediction results (Coefficient of Determination [R^2^]) for verbal and non-verbal cognitive functions from SC across samples, i.e. total, older and younger.

| SC |  | Total | | Older | | Younger | |
| --- | --- | --- | --- | --- | --- | --- | --- |
| Mod. | Tar. | EN | SVR | EN | SVR | EN | SVR |
| DMN | SF | 0.03  (0.03) | 0.02  (0.04) | -0.02  (0.03) | -0.05  (0.05) | -0.04  (0.07) | -0.05  (0.04) |
|  | PF | 0.00  (0.03) | -0.04  (0.04) | -0.02  (0.03) | -0.06  (0.05) | -0.02  (0.03) | -0.06  (0.08) |
|  | VF | 0.02  (0.02) | -0.01  (0.05) | -0.03  (0.03) | -0.07  (0.04) | -0.02  (0.02) | -0.06  (0.06) |
|  | VOC | 0.01  (0.02) | -0.02  (0.03) | -0.02  (0.03) | -0.05  (0.05) | -0.03  (0.04) | -0.03  (0.05) |
|  | VER | 0.03  (0.04) | 0.01  (0.05) | -0.03  (0.03) | -0.06  (0.08) | -0.02  (0.03) | -0.06  (0.06) |
|  | TMT-A | 0.17  (0.04) | 0.15  (0.06) | 0.00  (0.03) | -0.05  (0.07) | 0.01  (0.08) | 0.02  (0.08) |
|  | vWM | 0.17  (0.05) | 0.17  (0.06) | -0.03  (0.02) | -0.04  (0.05) | 0.04  (0.05) | 0.03  (0.06) |
| FPN | SF | 0.03  (0.02) | 0.02  (0.02) | -0.01  (0.02) | -0.02  (0.03) | -0.03  (0.04) | -0.02  (0.03) |
|  | PF | -0.01  (0.03) | -0.01  (0.03) | -0.02  (0.01) | -0.04  (0.03) | -0.01  (0.03) | -0.02  (0.03) |
|  | VF | 0.03  (0.03) | 0.03  (0.02) | -0.02  (0.03) | -0.03  (0.04) | -0.02  (0.02) | -0.02  (0.04) |
|  | VOC | -0.01  (0.02) | -0.01  (0.03) | -0.03  (0.04) | -0.04  (0.06) | -0.03  (0.04) | -0.03  (0.06) |
|  | VER | 0.02  (0.03) | 0.01  (0.03) | -0.04  (0.04) | -0.02  (0.03) | -0.02  (0.03) | -0.02  (0.03) |
|  | TMT-A | 0.19  (0.03) | 0.15  (0.04) | 0.01  (0.03) | -0.01  (0.05) | 0.02  (0.08) | -0.01  (0.09) |
|  | vWM | 0.14  (0.04) | 0.13  (0.05) | -0.03  (0.04) | -0.02  (0.04) | 0.00  (0.05) | 0.01  (0.05) |
| FPNDMN | SF | 0.02  (0.02) | -0.05  (0.05) | -0.03  (0.03) | -0.12  (0.06) | -0.04  (0.04) | -0.10  (0.11) |
|  | PF | 0.00  (0.02) | -0.09  (0.06) | -0.03  (0.03) | -0.14  (0.08) | -0.02  (0.03) | -0.08  (0.09) |
|  | VF | 0.02  (0.03) | -0.06  (0.06) | -0.04  (0.04) | -0.15  (0.07) | -0.06  (0.12) | -0.11  (0.11) |
|  | VOC | 0.01  (0.02) | -0.05  (0.05) | -0.02  (0.03) | -0.08  (0.07) | -0.06  (0.06) | -0.07  (0.09) |
|  | VER | 0.02  (0.03) | -0.04  (0.06) | -0.04  (0.05) | -0.13  (0.07) | -0.07  (0.14) | -0.09  (0.13) |
|  | TMT-A | 0.20  (0.05) | 0.18  (0.07) | 0.00  (0.04) | -0.05  (0.09) | 0.05  (0.07) | 0.04  (0.08) |
|  | vWM | 0.19  (0.05) | 0.18  (0.07) | -0.02  (0.03) | -0.06  (0.05) | 0.07  (0.05) | 0.04  (0.09) |
| WHOLE | SF | 0.00  (0.05) | -0.05  (0.07) | -0.03  (0.03) | -0.11  (0.11) | -0.04  (0.05) | -0.11  (0.08) |
|  | PF | -0.01  (0.03) | -0.04  (0.06) | -0.01  (0.06) | -0.01  (0.06) | -0.05  (0.08) | -0.07  (0.08) |
|  | VF | 0.02  (0.05) | -0.02  (0.06) | 0.00  (0.04) | -0.03  (0.07) | -0.05  (0.05) | -0.10  (0.07) |
|  | VOC | -0.01  (0.03) | -0.05  (0.05) | 0.01  (0.05) | -0.05  (0.06) | -0.05  (0.08) | -0.07  (0.07) |
|  | VER | 0.02  (0.04) | -0.01  (0.05) | -0.01  (0.06) | -0.01  (0.09) | -0.02  (0.03) | -0.10  (0.07) |
|  | TMT-A | 0.21  (0.07) | 0.19  (0.08) | 0.01  (0.05) | -0.03  (0.08) | 0.03  (0.10) | 0.06  (0.10) |
|  | vWM | 0.22  (0.05) | 0.21  (0.05) | -0.02  (0.04) | -0.07  (0.07) | 0.01  (0.09) | 0.01  (0.10) |

*Note.* Standard deviation (SD) appears in parentheses.

*Supplementary Table 10.* Prediction results for all targets from functional connectivity (FC) in the total sample.

| Total | FC |  | | DMN | | | | | | FPN | | | | | | FPNDMN | | | | | | WHOLE | | | | |
| --- | --- | --- | --- | --- | --- | --- | --- | --- | --- | --- | --- | --- | --- | --- | --- | --- | --- | --- | --- | --- | --- | --- | --- | --- | --- | --- |
| Tar. | Mod. | Alg. | | MAE | | R^2^ | | r | | MAE | | R^2^ | | r | | MAE | | R^2^ | | r | | MAE | | R^2^ | | r |
| SF | B | SVR | | 0.80 (0.03) | | 0.00 (0.05) | | 0.16 (0.01) | | 0.79 (0.04) | | 0.00 (0.04) | | 0.12 (0.02) | | 0.83 (0.04) | | -0.08 (0.06) | | 0.11 (0.01) | | 0.83 (0.06) | | -0.09 (0.08) | | 0.17 (0.03) |
|  |  | EN | | 0.78 (0.03) | | 0.02  (0.03) | | 0.18 (0.02) | | 0.79 (0.04) | | 0.01 (0.03) | | 0.11 (0.04) | | 0.78 (0.04) | | 0.03 (0.03) | | 0.17 (0.02) | | 0.79 (0.05) | | 0.02 (0.05) | | 0.18 (0.06) |
|  | B+D | SVR | | 0.80 (0.03) | | 0.00 (0.05) | | 0.17 (0.01) | | 0.79 (0.04) | | 0.01 (0.04) | | 0.14 (0.02) | | 0.83 (0.04) | | -0.07 (0.06) | | 0.11 (0.01) | | 0.83 (0.06) | | -0.09 (0.08) | | 0.18 (0.03) |
|  |  | EN | | 0.76 (0.03) | | 0.08 (0.02) | | 0.30 (0.01) | | 0.75 (0.04) | | 0.09 (0.04) | | 0.31 (0.01) | | 0.75 (0.04) | | 0.09 (0.03) | | 0.31 (0.03) | | 0.76 (0.05) | | 0.07 (0.03) | | 0.29 (0.04) |
| PF | B | SVR | | 0.81 (0.05) | | -0.02 (0.05) | | 0.13 (0.01) | | 0.80 (0.04) | | 0.00 (0.03) | | 0.10 (0.01) | | 0.83 (0.05) | | -0.07 (0.06) | | 0.12 (0.03) | | 0.84 (0.05) | | -0.13 (0.08) | | 0.11 (0.02) |
|  |  | EN | | 0.80 (0.05) | | 0.00 (0.03) | | 0.11 (0.01) | | 0.81 (0.04) | | -0.01 (0.02) | | 0.04 (0.03) | | 0.80 (0.04) | | 0.00 (0.03) | | 0.10 (0.02) | | 0.81 (0.05) | | -0.03 (0.04) | | 0.05 (0.03) |
|  | B+D | SVR | | 0.81 (0.05) | | -0.02 (0.05) | | 0.13 (0.01) | | 0.80 (0.04) | | 0.01 (0.03) | | 0.12 (0.01) | | 0.83 (0.05) | | -0.07 (0.06) | | 0.13 (0.03) | | 0.84 (0.05) | | -0.13 (0.08) | | 0.11 (0.02) |
|  |  | EN | | 0.80 (0.04) | | 0.02 (0.02) | | 0.16 (0.01) | | 0.79 (0.04) | | 0.03 (0.03) | | 0.17 (0.03) | | 0.80 (0.04) | | 0.02 (0.03) | | 0.15 (0.00) | | 0.80 (0.05) | | 0.01 (0.03) | | 0.14 (0.02) |
| VF | B | SVR | | 0.69 (0.03) | | -0.01 (0.06) | | 0.18 (0.02) | | 0.69 (0.03) | | 0.02 (0.04) | | 0.12 (0.03) | | 0.72 (0.04) | | -0.07 (0.06) | | 0.14 (0.02) | | 0.71 (0.04) | | -0.09 (0.08) | | 0.16 (0.01) |
|  |  | EN | | 0.69 (0.03) | | 0.02 (0.03) | | 0.17 (0.02) | | 0.69 (0.03) | | 0.01 (0.03) | | 0.12 (0.03) | | 0.69 (0.04) | | 0.03 (0.03) | | 0.17 (0.01) | | 0.69 (0.04) | | 0.00 (0.04) | | 0.14 (0.02) |
|  | B+D | SVR | | 0.69 (0.03) | | 0.00 (0.06) | | 0.19 (0.02) | | 0.69 (0.03) | | 0.01 (0.04) | | 0.14 (0.03) | | 0.72 (0.04) | | -0.07 (0.06) | | 0.14 (0.02) | | 0.71 (0.04) | | -0.09 (0.08) | | 0.16 (0.01) |
|  |  | EN | | 0.67 (0.03) | | 0.07 (0.03) | | 0.28 (0.01) | | 0.66 (0.03) | | 0.09 (0.04) | | 0.30 (0.02) | | 0.67 (0.04) | | 0.07 (0.03) | | 0.28 (0.00) | | 0.67 (0.04) | | 0.07 (0.03) | | 0.28 (0.02) |
| VOC | B | SVR | | 0.80 (0.05) | | -0.03 (0.04) | | 0.12 (0.01) | | 0.79 (0.04) | | -0.01 (0.04) | | 0.10 (0.02) | | 0.81 (0.04) | | -0.07 (0.06) | | 0.12 (0.04) | | 0.85 (0.03) | | -0.14 (0.07) | | 0.10 (0.03) |
|  |  | EN | | 0.80 (0.05) | | -0.01 (0.03) | | 0.06 (0.02) | | 0.80 (0.04) | | -0.01 (0.02) | | 0.06 (0.02) | | 0.80 (0.04) | | 0.00 (0.03) | | 0.09 (0.04) | | 0.81 (0.04) | | -0.03 (0.03) | | 0.03 (0.04) |
|  | B+D | SVR | | 0.79 (0.05) | | -0.01 (0.04) | | 0.15 (0.01) | | 0.79 (0.04) | | 0.00 (0.04) | | 0.17 (0.01) | | 0.80 (0.04) | | -0.05 (0.06) | | 0.14 (0.04) | | 0.85 (0.03) | | -0.14 (0.07) | | 0.10 (0.03) |
|  |  | EN | | 0.69 (0.05) | | 0.21 (0.05) | | 0.47 (0.00) | | 0.69 (0.04) | | 0.21 (0.05) | | 0.47 (0.00) | | 0.70 (0.04) | | 0.20 (0.05) | | 0.46 (0.00) | | 0.70 (0.04) | | 0.19 (0.05) | | 0.45 (0.00) |
| VER | B | SVR | | 0.63 (0.03) | | -0.01 (0.07) | | 0.18 (0.02) | | 0.63 (0.03) | | 0.00 (0.03) | | 0.11 (0.01) | | 0.64 (0.03) | | -0.06 (0.07) | | 0.16 (0.01) | | 0.65 (0.03) | | -0.07 (0.07) | | 0.18 (0.02) |
|  |  | EN | | 0.62 (0.03) | | 0.03 (0.04) | | 0.19 (0.03) | | 0.63 (0.03) | | 0.00 (0.03) | | 0.10 (0.03) | | 0.62 (0.03) | | 0.03 (0.03) | | 0.18 (0.02) | | 0.62 (0.03) | | 0.01 (0.03) | | 0.16 (0.02) |
|  | B+D | SVR | | 0.63 (0.03) | | 0.00 (0.07) | | 0.20 (0.02) | | 0.63 (0.03) | | 0.02 (0.03) | | 0.16 (0.01) | | 0.63 (0.03) | | -0.05 (0.06) | | 0.17 (0.01) | | 0.65 (0.03) | | -0.07 (0.07) | | 0.18 (0.02) |
|  |  | EN | | 0.57 (0.02) | | 0.16 (0.04) | | 0.41 (0.02) | | 0.57 (0.03) | | 0.16 (0.05) | | 0.41 (0.01) | | 0.57 (0.03) | | 0.16 (0.05) | | 0.41 (0.01) | | 0.57 (0.03) | | 0.15 (0.04) | | 0.41 (0.01) |
| TMT-A | B | SVR | | 0.75 (0.06) | | 0.05 (0.06) | | 0.29 (0.01) | | 0.75 (0.05) | | 0.07 (0.06) | | 0.31 (0.01) | | 0.72 (0.04) | | -0.07 (0.06) | | 0.14 (0.02) | | 0.75 (0.03) | | 0.10 (0.06) | | 0.38 (0.01) |
|  |  | EN | | 0.76 (0.05) | | 0.08 (0.05) | | 0.30 (0.03) | | 0.75 (0.05) | | 0.09 (0.05) | | 0.32 (0.02) | | 0.69 (0.04) | | 0.03 (0.03) | | 0.17 (0.01) | | 0.73 (0.03) | | 0.14 (0.04) | | 0.39 (0.01) |
|  | B+D | SVR | | 0.74 (0.06) | | 0.07 (0.06) | | 0.31 (0.01) | | 0.72 (0.05) | | 0.13 (0.08) | | 0.40 (0.03) | | 0.72 (0.04) | | -0.07 (0.06) | | 0.14 (0.02) | | 0.75 (0.03) | | 0.10 (0.06) | | 0.38 (0.01) |
|  |  | EN | | 0.64 (0.05) | | 0.32 (0.06) | | 0.58 (0.00) | | 0.64 (0.04) | | 0.33 (0.05) | | 0.58 (0.00) | | 0.67 (0.04) | | 0.07 (0.03) | | 0.28 (0.00) | | 0.64 (0.03) | | 0.32 (0.04) | | 0.58 (0.01) |
| vWM | B | SVR | | 0.61 (0.04) | | 0.13 (0.08) | | 0.39 (0.02) | | 0.63 (0.04) | | 0.05 (0.04) | | 0.27 (0.01) | | 0.61 (0.03) | | 0.12 (0.07) | | 0.39 (0.04) | | 0.59 (0.03) | | 0.17 (0.07) | | 0.44 (0.02) |
|  |  | EN | | 0.60 (0.04) | | 0.15 (0.06) | | 0.40 (0.01) | | 0.64 (0.04) | | 0.07 (0.05) | | 0.28 (0.01) | | 0.60 (0.03) | | 0.16 (0.05) | | 0.41 (0.02) | | 0.59 (0.03) | | 0.19 (0.07) | | 0.45 (0.01) |
|  | B+D | SVR | | 0.60 (0.04) | | 0.15 (0.08) | | 0.42 (0.02) | | 0.60 (0.04) | | 0.14 (0.05) | | 0.39 (0.01) | | 0.60 (0.03) | | 0.14 (0.07) | | 0.41 (0.03) | | 0.59 (0.03) | | 0.17 (0.07) | | 0.44 (0.02) |
|  |  | EN | 0.51 (0.04) | | 0.37 (0.06) | | 0.62 (0.00) | | 0.51 (0.03) | | 0.37 (0.05) | | 0.62 (0.00) | | 0.51 (0.04) | | 0.37 (0.06) | | 0.62 (0.00) | | 0.52 (0.02) | | 0.36 (0.05) | | 0.61 (0.01) | |

*Note.* Standard deviation (SD) appears in parentheses.

*Supplementary Table 11.* Prediction results for all targets from structural connectivity (SC) in the total sample.

| Total | SC |  | DMN | | | FPN | | | FPNDMN | | | WHOLE | | |
| --- | --- | --- | --- | --- | --- | --- | --- | --- | --- | --- | --- | --- | --- | --- |
| Tar. | Mod. | Alg. | MAE | R^2^ | r | MAE | R^2^ | r | MAE | R^2^ | r | MAE | R^2^ | r |
| SF | B | SVR | 0.78 (0.03) | 0.02 (0.04) | 0.19 (0.03) | 0.79 (0.04) | 0.02 (0.02) | 0.17 (0.02) | 0.81 (0.04) | -0.05 (0.05) | 0.13 (0.05) | 0.81 (0.04) | -0.05 (0.07) | 0.16 (0.01) |
|  |  | EN | 0.78 (0.02) | 0.03 (0.03) | 0.19 (0.03) | 0.78 (0.04) | 0.03 (0.02) | 0.18 (0.01) | 0.79 (0.04) | 0.02 (0.02) | 0.16 (0.03) | 0.79 (0.04) | 0.00 (0.05) | 0.15 (0.01) |
|  | B+D | SVR | 0.78 (0.03) | 0.02 (0.04) | 0.20 (0.03) | 0.79 (0.04) | 0.03 (0.02) | 0.18 (0.02) | 0.81 (0.04) | -0.04 (0.05) | 0.14 (0.05) | 0.81 (0.04) | -0.05 (0.07) | 0.16 (0.01) |
|  |  | EN | 0.76 (0.03) | 0.07 (0.03) | 0.28 (0.02) | 0.76 (0.04) | 0.08 (0.02) | 0.31 (0.02) | 0.77 (0.04) | 0.06 (0.02) | 0.26 (0.01) | 0.78 (0.04) | 0.04 (0.04) | 0.22 (0.03) |
| PF | B | SVR | 0.81 (0.04) | -0.04 (0.04) | 0.08 (0.01) | 0.81 (0.05) | -0.01 (0.03) | 0.09 (0.02) | 0.84 (0.04) | -0.09 (0.06) | 0.07 (0.01) | 0.82 (0.05) | -0.04 (0.06) | 0.14 (0.05) |
|  |  | EN | 0.80 (0.04) | 0.00 (0.03) | 0.08 (0.03) | 0.81 (0.05) | -0.01 (0.03) | 0.06 (0.02) | 0.80 (0.04) | 0.00 (0.02) | 0.08 (0.02) | 0.81 (0.04) | -0.01 (0.03) | 0.08 (0.01) |
|  | B+D | SVR | 0.81 (0.04) | -0.03 (0.04) | 0.08 (0.01) | 0.81 (0.05) | -0.01 (0.03) | 0.10 (0.02) | 0.83 (0.04) | -0.09 (0.06) | 0.07 (0.01) | 0.82 (0.05) | -0.04 (0.06) | 0.14 (0.05) |
|  |  | EN | 0.80 (0.04) | 0.02 (0.03) | 0.16 (0.05) | 0.79 (0.05) | 0.03 (0.03) | 0.19 (0.02) | 0.80 (0.04) | 0.02 (0.02) | 0.16 (0.03) | 0.80 (0.05) | 0.01 (0.03) | 0.13 (0.04) |
| VF | B | SVR | 0.69 (0.03) | -0.01 (0.05) | 0.16 (0.01) | 0.68 (0.04) | 0.03 (0.03) | 0.19 (0.02) | 0.70 (0.04) | -0.06 (0.06) | 0.13 (0.04) | 0.69 (0.04) | -0.02 (0.06) | 0.18 (0.02) |
|  |  | EN | 0.69 (0.03) | 0.02 (0.02) | 0.17 (0.00) | 0.68 (0.04) | 0.03 (0.02) | 0.18 (0.02) | 0.68 (0.04) | 0.02 (0.03) | 0.17 (0.01) | 0.68 (0.04) | 0.02 (0.05) | 0.19 (0.04) |
|  | B+D | SVR | 0.69 (0.03) | 0.00 (0.05) | 0.17 (0.01) | 0.68 (0.04) | 0.03 (0.03) | 0.20 (0.03) | 0.70 (0.04) | -0.06 (0.06) | 0.14 (0.04) | 0.69 (0.04) | -0.02 (0.06) | 0.18 (0.02) |
|  |  | EN | 0.68 (0.03) | 0.05 (0.05) | 0.24 (0.06) | 0.67 (0.04) | 0.07 (0.03) | 0.28 (0.03) | 0.67 (0.04) | 0.06 (0.03) | 0.26 (0.02) | 0.67 (0.04) | 0.05 (0.05) | 0.24 (0.01) |
| VOC | B | SVR | 0.80 (0.04) | -0.02 (0.03) | 0.11 (0.03) | 0.80 (0.04) | -0.01 (0.03) | 0.10 (0.01) | 0.81 (0.04) | -0.05 (0.05) | 0.12 (0.03) | 0.81 (0.04) | -0.05 (0.05) | 0.11 (0.03) |
|  |  | EN | 0.80 (0.03) | 0.01 (0.02) | 0.10 (0.02) | 0.80 (0.04) | -0.01 (0.02) | 0.06 (0.04) | 0.80 (0.04) | 0.01 (0.02) | 0.12 (0.03) | 0.80 (0.04) | -0.01 (0.03) | 0.09 (0.03) |
|  | B+D | SVR | 0.79 (0.03) | 0.00 (0.03) | 0.15 (0.03) | 0.79 (0.03) | 0.00 (0.04) | 0.17 (0.02) | 0.81 (0.04) | -0.03 (0.05) | 0.14 (0.03) | 0.81 (0.04) | -0.05 (0.05) | 0.12 (0.03) |
|  |  | EN | 0.69 (0.04) | 0.22 (0.04) | 0.48 (0.00) | 0.69 (0.04) | 0.21 (0.04) | 0.47 (0.00) | 0.70 (0.05) | 0.21 (0.04) | 0.47 (0.00) | 0.70 (0.03) | 0.20 (0.03) | 0.46 (0.01) |
| VER | B | SVR | 0.62 (0.03) | 0.01 (0.05) | 0.19 (0.02) | 0.62 (0.03) | 0.01 (0.03) | 0.15 (0.02) | 0.63 (0.03) | -0.04 (0.06) | 0.16 (0.02) | 0.62 (0.04) | -0.01 (0.05) | 0.19 (0.02) |
|  |  | EN | 0.62 (0.03) | 0.03 (0.04) | 0.19 (0.03) | 0.63 (0.03) | 0.02 (0.03) | 0.15 (0.03) | 0.62 (0.03) | 0.02 (0.03) | 0.18 (0.01) | 0.62 (0.04) | 0.02 (0.04) | 0.20 (0.03) |
|  | B+D | SVR | 0.61 (0.03) | 0.03 (0.05) | 0.22 (0.02) | 0.62 (0.03) | 0.02 (0.04) | 0.19 (0.02) | 0.63 (0.03) | -0.03 (0.06) | 0.18 (0.02) | 0.62 (0.04) | -0.01 (0.05) | 0.19 (0.02) |
|  |  | EN | 0.58 (0.02) | 0.14 (0.04) | 0.39 (0.02) | 0.58 (0.03) | 0.15 (0.04) | 0.40 (0.00) | 0.57 (0.03) | 0.16 (0.04) | 0.41 (0.01) | 0.58 (0.03) | 0.15 (0.04) | 0.40 (0.00) |
| TMT-A | B | SVR | 0.71 (0.05) | 0.15 (0.06) | 0.42 (0.02) | 0.72 (0.04) | 0.15 (0.04) | 0.42 (0.03) | 0.70 (0.03) | 0.18 (0.07) | 0.45 (0.01) | 0.71 (0.03) | 0.19 (0.08) | 0.46 (0.00) |
|  |  | EN | 0.72 (0.04) | 0.17 (0.04) | 0.43 (0.02) | 0.72 (0.04) | 0.19 (0.03) | 0.44 (0.02) | 0.71 (0.03) | 0.20 (0.05) | 0.45 (0.02) | 0.70 (0.04) | 0.21 (0.07) | 0.46 (0.01) |
|  | B+D | SVR | 0.70 (0.05) | 0.16 (0.06) | 0.43 (0.02) | 0.71 (0.03) | 0.16 (0.04) | 0.43 (0.01) | 0.70 (0.03) | 0.18 (0.06) | 0.45 (0.01) | 0.71 (0.03) | 0.19 (0.08) | 0.46 (0.00) |
|  |  | EN | 0.64 (0.04) | 0.32 (0.05) | 0.58 (0.00) | 0.64 (0.04) | 0.32 (0.03) | 0.58 (0.01) | 0.64 (0.03) | 0.33 (0.04) | 0.58 (0.00) | 0.64 (0.04) | 0.32 (0.06) | 0.58 (0.00) |
| vWM | B | SVR | 0.59 (0.04) | 0.17 (0.06) | 0.42 (0.01) | 0.60 (0.03) | 0.13 (0.05) | 0.38 (0.03) | 0.58 (0.04) | 0.18 (0.07) | 0.44 (0.02) | 0.57 (0.03) | 0.21 (0.05) | 0.46 (0.03) |
|  |  | EN | 0.59 (0.04) | 0.17 (0.05) | 0.42 (0.01) | 0.60 (0.03) | 0.14 (0.04) | 0.39 (0.01) | 0.58 (0.04) | 0.19 (0.05) | 0.44 (0.01) | 0.57 (0.03) | 0.22 (0.05) | 0.47 (0.01) |
|  | B+D | SVR | 0.58 (0.04) | 0.18 (0.06) | 0.44 (0.01) | 0.59 (0.03) | 0.16 (0.05) | 0.40 (0.01) | 0.58 (0.04) | 0.19 (0.07) | 0.45 (0.02) | 0.57 (0.03) | 0.21 (0.05) | 0.47 (0.03) |
|  |  | EN | 0.52 (0.04) | 0.36 (0.05) | 0.61 (0.01) | 0.51 (0.03) | 0.36 (0.06) | 0.62 (0.01) | 0.52 (0.03) | 0.36 (0.04) | 0.61 (0.00) | 0.52 (0.03) | 0.35 (0.02) | 0.61 (0.00) |

*Note.* Standard deviation (SD) appears in parentheses.

*Supplementary Table 12.* Prediction results for all targets from functional connectivity (FC) in the older sample.

| Older | FC |  | DMN | | | FPN | | | FPNDMN | | | WHOLE | | |
| --- | --- | --- | --- | --- | --- | --- | --- | --- | --- | --- | --- | --- | --- | --- |
| Tar. | Mod. | Alg. | MAE | R^2^ | r | MAE | R^2^ | r | MAE | R^2^ | r | MAE | R^2^ | r |
| SF | B | SVR | 0.81 (0.05) | -0.05 (0.05) | 0.05 (0.07) | 0.79 (0.06) | -0.01 (0.04) | 0.09 (0.00) | 0.84 (0.06) | -0.11 (0.06) | 0.04 (0.00) | 0.82 (0.05) | -0.10 (0.12) | 0.12 (0.01) |
|  |  | EN | 0.80 (0.05) | -0.03 (0.02) | -0.04 (0.04) | 0.79 (0.06) | -0.01 (0.03) | 0.03 (0.03) | 0.79 (0.05) | -0.02 (0.02) | -0.05 (0.04) | 0.80 (0.05) | -0.03 (0.05) | 0.01 (0.06) |
|  | B+D | SVR | 0.81 (0.05) | -0.05 (0.05) | 0.05 (0.07) | 0.79 (0.06) | 0.00 (0.04) | 0.10 (0.00) | 0.84 (0.06) | -0.11 (0.06) | 0.04 (0.00) | 0.82 (0.05) | -0.10 (0.12) | 0.12 (0.01) |
|  |  | EN | 0.79 (0.05) | -0.01 (0.03) | 0.05 (0.03) | 0.78 (0.07) | 0.01 (0.04) | 0.14 (0.01) | 0.79 (0.06) | -0.01 (0.02) | 0.07 (0.05) | 0.79 (0.06) | -0.02 (0.04) | 0.05 (0.05) |
| PF | B | SVR | 0.80 (0.06) | -0.03 (0.05) | 0.09 (0.07) | 0.81 (0.06) | -0.03 (0.05) | 0.05 (0.01) | 0.82 (0.06) | -0.07 (0.08) | 0.11 (0.02) | 0.81 (0.06) | -0.07 (0.09) | 0.14 (0.02) |
|  |  | EN | 0.80 (0.07) | -0.01 (0.02) | 0.06 (0.04) | 0.80 (0.06) | -0.01 (0.05) | 0.09 (0.04) | 0.81 (0.06) | -0.02 (0.03) | 0.06 (0.04) | 0.81 (0.06) | -0.05 (0.06) | 0.06 (0.03) |
|  | B+D | SVR | 0.80 (0.06) | -0.03 (0.05) | 0.09 (0.07) | 0.81 (0.06) | -0.03 (0.06) | 0.06 (0.01) | 0.81 (0.06) | -0.06 (0.08) | 0.12 (0.02) | 0.81 (0.06) | -0.07 (0.09) | 0.14 (0.02) |
|  |  | EN | 0.79 (0.06) | 0.03 (0.05) | 0.20 (0.06) | 0.77 (0.06) | 0.06 (0.04) | 0.26 (0.02) | 0.79 (0.06) | 0.01 (0.03) | 0.14 (0.02) | 0.80 (0.06) | -0.02 (0.06) | 0.09 (0.03) |
| VF | B | SVR | 0.68 (0.04) | -0.01 (0.04) | 0.15 (0.03) | 0.69 (0.04) | -0.02 (0.05) | 0.08 (0.03) | 0.71 (0.05) | -0.08 (0.09) | 0.11 (0.01) | 0.69 (0.05) | -0.05 (0.08) | 0.17 (0.01) |
|  |  | EN | 0.69 (0.04) | -0.01 (0.04) | 0.10 (0.05) | 0.69 (0.04) | 0.00 (0.03) | 0.09 (0.04) | 0.69 (0.05) | -0.02 (0.03) | 0.06 (0.02) | 0.69 (0.05) | -0.04 (0.07) | 0.07 (0.05) |
|  | B+D | SVR | 0.68 (0.04) | 0.00 (0.04) | 0.16 (0.03) | 0.69 (0.04) | -0.01 (0.05) | 0.10 (0.03) | 0.70 (0.05) | -0.07 (0.09) | 0.12 (0.01) | 0.69 (0.05) | -0.05 (0.08) | 0.17 (0.01) |
|  |  | EN | 0.67 (0.04) | 0.04 (0.05) | 0.22 (0.07) | 0.66 (0.04) | 0.08 (0.05) | 0.29 (0.00) | 0.67 (0.04) | 0.02 (0.05) | 0.19 (0.05) | 0.68 (0.05) | 0.01 (0.08) | 0.16 (0.09) |
| VOC | B | SVR | 0.80 (0.04) | -0.02 (0.07) | 0.13 (0.03) | 0.81 (0.05) | -0.01 (0.04) | 0.09 (0.03) | 0.83 (0.07) | -0.09 (0.10) | 0.09 (0.02) | 0.84 (0.05) | -0.14 (0.08) | 0.07 (0.02) |
|  |  | EN | 0.81 (0.05) | -0.01 (0.04) | 0.06 (0.02) | 0.81 (0.05) | -0.01 (0.03) | 0.05 (0.07) | 0.81 (0.06) | -0.03 (0.06) | 0.03 (0.02) | 0.82 (0.06) | -0.03 (0.04) | -0.05 (0.05) |
|  | B+D | SVR | 0.80 (0.04) | 0.00 (0.07) | 0.15 (0.03) | 0.81 (0.05) | -0.01 (0.05) | 0.12 (0.03) | 0.82 (0.07) | -0.08 (0.09) | 0.10 (0.02) | 0.84 (0.05) | -0.14 (0.08) | 0.07 (0.02) |
|  |  | EN | 0.70 (0.04) | 0.19 (0.05) | 0.46 (0.01) | 0.70 (0.06) | 0.21 (0.06) | 0.47 (0.01) | 0.71 (0.05) | 0.19 (0.07) | 0.45 (0.01) | 0.71 (0.05) | 0.18 (0.04) | 0.47 (0.03) |
| VER | B | SVR | 0.62 (0.04) | 0.00 (0.05) | 0.19 (0.03) | 0.63 (0.04) | -0.02 (0.06) | 0.08 (0.02) | 0.64 (0.05) | -0.06 (0.09) | 0.15 (0.00) | 0.63 (0.04) | -0.04 (0.08) | 0.18 (0.02) |
|  |  | EN | 0.62 (0.04) | 0.02 (0.04) | 0.18 (0.02) | 0.63 (0.04) | -0.01 (0.06) | 0.10 (0.04) | 0.64 (0.04) | -0.01 (0.04) | 0.11 (0.05) | 0.63 (0.04) | -0.01 (0.06) | 0.13 (0.03) |
|  | B+D | SVR | 0.61 (0.03) | 0.02 (0.05) | 0.21 (0.02) | 0.63 (0.04) | -0.01 (0.07) | 0.13 (0.02) | 0.66 (0.05) | -0.05 (0.09) | 0.16 (0.00) | 0.63 (0.04) | -0.03 (0.08) | 0.18 (0.02) |
|  |  | EN | 0.57 (0.04) | 0.14 (0.05) | 0.39 (0.03) | 0.56 (0.03) | 0.18 (0.05) | 0.44 (0.01) | 0.57 (0.04) | 0.15 (0.06) | 0.41 (0.03) | 0.57 (0.04) | 0.15 (0.06) | 0.40 (0.00) |
| TMT-A | B | SVR | 0.81 (0.05) | -0.04 (0.06) | 0.11 (0.01) | 0.79 (0.05) | -0.02 (0.05) | 0.12 (0.03) | 0.84 (0.05) | -0.12 (0.08) | 0.05 (0.03) | 0.85 (0.06) | -0.10 (0.08) | 0.11 (0.02) |
|  |  | EN | 0.81 (0.05) | -0.02 (0.04) | 0.06 (0.05) | 0.80 (0.05) | 0.00 (0.03) | 0.11 (0.02) | 0.81 (0.04) | -0.02 (0.04) | 0.08 (0.04) | 0.81 (0.05) | -0.01 (0.02) | 0.03 (0.02) |
|  | B+D | SVR | 0.80 (0.05) | -0.03 (0.06) | 0.12 (0.01) | 0.79 (0.05) | -0.01 (0.04) | 0.13 (0.02) | 0.84 (0.05) | -0.11 (0.08) | 0.05 (0.03) | 0.84 (0.06) | -0.10 (0.08) | 0.11 (0.02) |
|  |  | EN | 0.76 (0.05) | 0.07 (0.05) | 0.29 (0.03) | 0.76 (0.05) | 0.10 (0.04) | 0.33 (0.01) | 0.76 (0.04) | 0.08 (0.05) | 0.30 (0.02) | 0.77 (0.05) | 0.08 (0.03) | 0.32 (0.02) |
| vWM | B | SVR | 0.63 (0.04) | -0.03 (0.07) | 0.13 (0.06) | 0.60 (0.04) | 0.02 (0.07) | 0.19 (0.02) | 0.63 (0.05) | -0.08 (0.09) | 0.13 (0.07) | 0.64 (0.06) | -0.11 (0.12) | 0.11 (0.01) |
|  |  | EN | 0.62 (0.04) | 0.00 (0.03) | 0.10 (0.04) | 0.60 (0.04) | 0.03 (0.05) | 0.19 (0.04) | 0.62 (0.05) | 0.00 (0.06) | 0.12 (0.07) | 0.62 (0.07) | -0.01 (0.08) | 0.10 (0.10) |
|  | B+D | SVR | 0.62 (0.04) | -0.01 (0.07) | 0.15 (0.06) | 0.60 (0.04) | 0.03 (0.07) | 0.22 (0.02) | 0.63 (0.05) | -0.07 (0.09) | 0.14 (0.07) | 0.64 (0.06) | -0.10 (0.12) | 0.11 (0.01) |
|  |  | EN | 0.58 (0.03) | 0.10 (0.04) | 0.35 (0.02) | 0.57 (0.04) | 0.14 (0.06) | 0.38 (0.03) | 0.59 (0.04) | 0.08 (0.05) | 0.31 (0.02) | 0.59 (0.05) | 0.09 (0.06) | 0.32 (0.06) |

*Note.* Standard deviation (SD) appears in parentheses.

*Supplementary Table 13.* Prediction results for all targets from structural connectivity (SC) in the older sample.

| Older | SC |  | DMN | | | FPN | | | FPNDMN | | | WHOLE | | |
| --- | --- | --- | --- | --- | --- | --- | --- | --- | --- | --- | --- | --- | --- | --- |
| Tar. | Mod. | Alg. | MAE | R^2^ | r | MAE | R^2^ | r | MAE | R^2^ | r | MAE | R^2^ | r |
| SF | B | SVR | 0.80 (0.04) | -0.05 (0.05) | 0.05 (0.03) | 0.80 (0.05) | -0.02 (0.03) | 0.02 (0.07) | 0.82 (0.06) | -0.12 (0.06) | -0.02 (0.05) | 0.83 (0.07) | -0.11 (0.11) | 0.04 (0.02) |
|  |  | EN | 0.79 (0.05) | -0.02 (0.03) | 0.02 (0.06) | 0.79 (0.05) | -0.01 (0.02) | 0.05 (0.04) | 0.79 (0.05) | -0.03 (0.03) | -0.05 (0.02) | 0.80 (0.07) | -0.03 (0.03) | -0.06 (0.05) |
|  | B+D | SVR | 0.80 (0.04) | -0.04 (0.05) | 0.05 (0.03) | 0.80 (0.05) | -0.02 (0.03) | 0.04 (0.07) | 0.82 (0.06) | -0.12 (0.06) | -0.02 (0.05) | 0.83 (0.07) | -0.11 (0.11) | 0.04 (0.02) |
|  |  | EN | 0.79 (0.05) | -0.01 (0.04) | 0.04 (0.05) | 0.78 (0.05) | 0.03 (0.03) | 0.21 (0.05) | 0.79 (0.05) | -0.01 (0.03) | 0.05 (0.02) | 0.79 (0.07) | -0.02 (0.03) | 0.00 (0.01) |
| PF | B | SVR | 0.82 (0.06) | -0.06 (0.05) | 0.00 (0.01) | 0.81 (0.07) | -0.04 (0.03) | -0.02 (0.03) | 0.86 (0.07) | -0.14 (0.08) | -0.03 (0.02) | 0.81 (0.05) | -0.01 (0.06) | 0.17 (0.02) |
|  |  | EN | 0.81 (0.05) | -0.02 (0.03) | -0.09 (0.01) | 0.81 (0.07) | -0.02 (0.01) | -0.04 (0.01) | 0.81 (0.06) | -0.03 (0.03) | -0.07 (0.03) | 0.81 (0.06) | -0.01 (0.06) | 0.12 (0.05) |
|  | B+D | SVR | 0.82 (0.06) | -0.05 (0.05) | 0.01 (0.01) | 0.81 (0.07) | -0.03 (0.03) | 0.00 (0.04) | 0.85 (0.07) | -0.14 (0.08) | -0.02 (0.02) | 0.81 (0.05) | -0.01 (0.06) | 0.17 (0.02) |
|  |  | EN | 0.79 (0.06) | 0.01 (0.03) | 0.15 (0.02) | 0.78 (0.07) | 0.04 (0.03) | 0.22 (0.06) | 0.80 (0.06) | 0.00 (0.03) | 0.13 (0.03) | 0.79 (0.06) | 0.03 (0.04) | 0.20 (0.03) |
| VF | B | SVR | 0.70 (0.05) | -0.07 (0.04) | 0.00 (0.02) | 0.69 (0.06) | -0.03 (0.04) | 0.03 (0.08) | 0.73 (0.04) | -0.15 (0.07) | -0.04 (0.05) | 0.69 (0.05) | -0.03 (0.07) | 0.15 (0.03) |
|  |  | EN | 0.69 (0.04) | -0.03 (0.03) | -0.04 (0.06) | 0.69 (0.06) | -0.02 (0.03) | -0.02 (0.01) | 0.69 (0.03) | -0.04 (0.04) | -0.11 (0.07) | 0.68 (0.05) | 0.00 (0.04) | 0.14 (0.03) |
|  | B+D | SVR | 0.70 (0.05) | -0.06 (0.04) | 0.01 (0.02) | 0.69 (0.06) | -0.02 (0.04) | 0.05 (0.08) | 0.73 (0.04) | -0.15 (0.07) | -0.03 (0.05) | 0.69 (0.05) | -0.03 (0.07) | 0.15 (0.03) |
|  |  | EN | 0.68 (0.04) | 0.02 (0.04) | 0.18 (0.01) | 0.67 (0.06) | 0.05 (0.04) | 0.25 (0.02) | 0.68 (0.03) | 0.01 (0.04) | 0.18 (0.03) | 0.67 (0.05) | 0.04 (0.04) | 0.24 (0.05) |
| VOC | B | SVR | 0.83 (0.06) | -0.05 (0.05) | 0.01 (0.06) | 0.81 (0.07) | -0.04 (0.06) | 0.04 (0.03) | 0.83 (0.07) | -0.08 (0.07) | 0.06 (0.02) | 0.81 (0.06) | -0.05 (0.06) | 0.11 (0.04) |
|  |  | EN | 0.81 (0.06) | -0.02 (0.03) | 0.01 (0.05) | 0.82 (0.07) | -0.03 (0.04) | -0.01 (0.04) | 0.81 (0.07) | -0.02 (0.03) | 0.03 (0.05) | 0.80 (0.06) | 0.01 (0.05) | 0.13 (0.04) |
|  | B+D | SVR | 0.82 (0.06) | -0.04 (0.05) | 0.05 (0.06) | 0.80 (0.07) | -0.01 (0.05) | 0.14 (0.01) | 0.83 (0.07) | -0.07 (0.07) | 0.08 (0.02) | 0.81 (0.06) | -0.04 (0.06) | 0.11 (0.04) |
|  |  | EN | 0.70 (0.05) | 0.20 (0.07) | 0.47 (0.03) | 0.71 (0.05) | 0.19 (0.06) | 0.45 (0.02) | 0.71 (0.05) | 0.19 (0.05) | 0.48 (0.01) | 0.70 (0.06) | 0.20 (0.04) | 0.48 (0.03) |
| VER | B | SVR | 0.64 (0.04) | -0.06 (0.08) | 0.06 (0.04) | 0.63 (0.06) | -0.02 (0.03) | 0.04 (0.07) | 0.66 (0.04) | -0.13 (0.07) | 0.02 (0.04) | 0.63 (0.05) | -0.01 (0.09) | 0.18 (0.01) |
|  |  | EN | 0.64 (0.04) | -0.03 (0.03) | 0.01 (0.00) | 0.64 (0.06) | -0.04 (0.04) | -0.04 (0.04) | 0.64 (0.04) | -0.04 (0.05) | -0.07 (0.03) | 0.63 (0.05) | -0.01 (0.06) | 0.14 (0.04) |
|  | B+D | SVR | 0.64 (0.04) | -0.04 (0.07) | 0.09 (0.04) | 0.63 (0.06) | -0.02 (0.07) | 0.08 (0.09) | 0.66 (0.04) | -0.12 (0.07) | 0.03 (0.04) | 0.63 (0.05) | -0.01 (0.09) | 0.18 (0.01) |
|  |  | EN | 0.57 (0.04) | 0.13 (0.06) | 0.37 (0.02) | 0.57 (0.05) | 0.14 (0.06) | 0.39 (0.05) | 0.57 (0.03) | 0.13 (0.04) | 0.39 (0.01) | 0.58 (0.05) | 0.12 (0.07) | 0.38 (0.02) |
| TMT-A | B | SVR | 0.81 (0.06) | -0.05 (0.07) | 0.10 (0.03) | 0.79 (0.05) | -0.01 (0.05) | 0.16 (0.03) | 0.82 (0.07) | -0.05 (0.09) | 0.13 (0.04) | 0.82 (0.05) | -0.03 (0.08) | 0.16 (0.02) |
|  |  | EN | 0.80 (0.06) | 0.00 (0.03) | 0.12 (0.02) | 0.80 (0.05) | 0.01 (0.03) | 0.15 (0.04) | 0.81 (0.06) | 0.00 (0.04) | 0.14 (0.03) | 0.80 (0.05) | 0.01 (0.05) | 0.15 (0.02) |
|  | B+D | SVR | 0.80 (0.06) | -0.03 (0.04) | 0.13 (0.02) | 0.78 (0.05) | 0.00 (0.05) | 0.18 (0.00) | 0.82 (0.07) | -0.05 (0.09) | 0.14 (0.04) | 0.81 (0.05) | -0.03 (0.08) | 0.16 (0.02) |
|  |  | EN | 0.77 (0.06) | 0.08 (0.04) | 0.32 (0.01) | 0.76 (0.06) | 0.08 (0.06) | 0.31 (0.04) | 0.77 (0.05) | 0.08 (0.04) | 0.30 (0.02) | 0.77 (0.06) | 0.09 (0.04) | 0.34 (0.05) |
| vWM | B | SVR | 0.62 (0.04) | -0.04 (0.05) | 0.06 (0.03) | 0.62 (0.04) | -0.02 (0.04) | 0.06 (0.02) | 0.62 (0.04) | -0.06 (0.05) | 0.09 (0.03) | 0.63 (0.05) | -0.07 (0.07) | 0.07 (0.04) |
|  |  | EN | 0.62 (0.03) | -0.03 (0.02) | -0.01 (0.01) | 0.62 (0.04) | -0.03 (0.04) | -0.01 (0.07) | 0.62 (0.04) | -0.02 (0.03) | 0.02 (0.03) | 0.62 (0.05) | -0.02 (0.04) | 0.06 (0.03) |
|  | B+D | SVR | 0.62 (0.04) | -0.04 (0.05) | 0.08 (0.04) | 0.62 (0.04) | -0.02 (0.04) | 0.08 (0.00) | 0.62 (0.04) | -0.05 (0.05) | 0.10 (0.03) | 0.62 (0.05) | -0.07 (0.07) | 0.08 (0.04) |
|  |  | EN | 0.58 (0.03) | 0.10 (0.03) | 0.35 (0.03) | 0.58 (0.04) | 0.10 (0.05) | 0.36 (0.03) | 0.59 (0.04) | 0.08 (0.03) | 0.31 (0.02) | 0.59 (0.05) | 0.07 (0.03) | 0.32 (0.01) |

*Note.* Standard deviation (SD) appears in parentheses.

*Supplementary Table 14.* Prediction results for all targets from functional connectivity (FC) in the younger sample.

| Younger | FC |  | DMN | | | FPN | | | FPNDMN | | | WHOLE | | |
| --- | --- | --- | --- | --- | --- | --- | --- | --- | --- | --- | --- | --- | --- | --- |
| Tar. | Mod. | Alg. | MAE | R^2^ | r | MAE | R^2^ | r | MAE | R^2^ | r | MAE | R^2^ | r |
| SF | B | SVR | 0.80 (0.07) | -0.03 (0.07) | 0.09 (0.05) | 0.81 (0.06) | -0.03 (0.04) | 0.02 (0.02) | 0.84 (0.06) | -0.12 (0.08) | 0.03 (0.07) | 0.84 (0.06) | -0.13 (0.10) | 0.07 (0.04) |
|  |  | EN | 0.81 (0.06) | -0.03 (0.04) | -0.01 (0.01) | 0.81 (0.06) | -0.03 (0.03) | -0.04 (0.03) | 0.82 (0.05) | -0.04 (0.04) | -0.04 (0.07) | 0.81 (0.06) | -0.03 (0.05) | -0.03 (0.01) |
|  | B+D | SVR | 0.80 (0.07) | -0.03 (0.07) | 0.09 (0.05) | 0.81 (0.06) | -0.03 (0.04) | 0.02 (0.02) | 0.84 (0.06) | -0.12 (0.08) | 0.03 (0.07) | 0.84 (0.06) | -0.13 (0.10) | 0.07 (0.04) |
|  |  | EN | 0.81 (0.06) | -0.02 (0.04) | 0.05 (0.01) | 0.81 (0.06) | -0.02 (0.03) | 0.05 (0.01) | 0.81 (0.05) | -0.03 (0.03) | 0.00 (0.04) | 0.81 (0.06) | -0.04 (0.05) | -0.03 (0.08) |
| PF | B | SVR | 0.84 (0.08) | -0.09 (0.08) | -0.04 (0.03) | 0.82 (0.09) | -0.05 (0.06) | -0.04 (0.05) | 0.86 (0.06) | -0.17 (0.09) | -0.06 (0.05) | 0.87 (0.06) | -0.21 (0.10) | 0.14 (0.02) |
|  |  | EN | 0.82 (0.08) | -0.04 (0.05) | -0.10 (0.04) | 0.81 (0.09) | -0.03 (0.03) | -0.11 (0.08) | 0.81 (0.07) | -0.02 (0.03) | -0.12 (0.02) | 0.81 (0.07) | -0.04 (0.05) | 0.06 (0.03) |
|  | B+D | SVR | 0.84 (0.08) | -0.09 (0.08) | -0.04 (0.03) | 0.82 (0.09) | -0.05 (0.06) | -0.04 (0.05) | 0.86 (0.06) | -0.17 (0.09) | -0.06 (0.05) | 0.87 (0.06) | -0.21 (0.10) | 0.14 (0.02) |
|  |  | EN | 0.82 (0.08) | -0.04 (0.05) | -0.11 (0.05) | 0.81 (0.09) | -0.03 (0.03) | -0.11 (0.04) | 0.81 (0.07) | -0.04 (0.05) | -0.11 (0.06) | 0.81 (0.07) | -0.04 (0.05) | 0.09 (0.03) |
| VF | B | SVR | 0.72 (0.06) | -0.09 (0.07) | 0.00 (0.02) | 0.70 (0.06) | -0.05 (0.04) | -0.04 (0.03) | 0.75 (0.06) | -0.19 (0.10) | -0.04 (0.03) | 0.73 (0.05) | -0.16 (0.08) | 0.03 (0.03) |
|  |  | EN | 0.70 (0.06) | -0.05 (0.05) | -0.08 (0.05) | 0.69 (0.06) | -0.03 (0.02) | -0.06 (0.01) | 0.70 (0.06) | -0.03 (0.03) | -0.07 (0.04) | 0.70 (0.06) | -0.04 (0.05) | -0.10 (0.04) |
|  | B+D | SVR | 0.72 (0.06) | -0.09 (0.07) | 0.00 (0.02) | 0.70 (0.06) | -0.05 (0.05) | -0.03 (0.03) | 0.75 (0.06) | -0.19 (0.10) | -0.03 (0.03) | 0.73 (0.05) | -0.16 (0.08) | 0.03 (0.03) |
|  |  | EN | 0.70 (0.06) | -0.04 (0.04) | -0.05 (0.03) | 0.70 (0.06) | -0.03 (0.03) | -0.02 (0.05) | 0.70 (0.06) | -0.03 (0.03) | -0.06 (0.06) | 0.70 (0.06) | -0.04 (0.07) | -0.08 (0.07) |
| VOC | B | SVR | 0.80 (0.08) | -0.09 (0.06) | -0.03 (0.06) | 0.79 (0.07) | -0.05 (0.07) | 0.01 (0.05) | 0.82 (0.08) | -0.13 (0.08) | -0.01 (0.00) | 0.83 (0.08) | -0.13 (0.09) | 0.04 (0.02) |
|  |  | EN | 0.80 (0.08) | -0.04 (0.09) | -0.09 (0.03) | 0.80 (0.07) | -0.05 (0.06) | -0.06 (0.03) | 0.80 (0.07) | -0.03 (0.04) | -0.13 (0.03) | 0.80 (0.08) | -0.03 (0.05) | -0.07 (0.03) |
|  | B+D | SVR | 0.79 (0.08) | -0.08 (0.06) | -0.01 (0.06) | 0.79 (0.07) | -0.05 (0.08) | 0.04 (0.05) | 0.82 (0.08) | -0.12 (0.08) | 0.00 (0.00) | 0.83 (0.08) | -0.13 (0.09) | 0.04 (0.02) |
|  |  | EN | 0.73 (0.08) | 0.12 (0.07) | 0.37 (0.04) | 0.72 (0.06) | 0.13 (0.06) | 0.38 (0.02) | 0.72 (0.06) | 0.13 (0.04) | 0.40 (0.02) | 0.73 (0.07) | 0.12 (0.06) | 0.37 (0.03) |
| VER | B | SVR | 0.66 (0.05) | -0.13 (0.07) | -0.09 (0.03) | 0.63 (0.06) | -0.05 (0.05) | -0.05 (0.02) | 0.69 (0.06) | -0.24 (0.09) | -0.11 (0.02) | 0.66 (0.05) | -0.14 (0.07) | 0.18 (0.02) |
|  |  | EN | 0.62 (0.05) | -0.02 (0.03) | -0.07 (0.04) | 0.62 (0.05) | -0.03 (0.02) | -0.11 (0.04) | 0.63 (0.05) | -0.03 (0.03) | -0.10 (0.06) | 0.62 (0.05) | -0.03 (0.04) | 0.13 (0.03) |
|  | B+D | SVR | 0.66 (0.05) | -0.12 (0.07) | -0.08 (0.03) | 0.63 (0.06) | -0.04 (0.05) | -0.03 (0.02) | 0.64 (0.06) | -0.23 (0.09) | -0.10 (0.02) | 0.66 (0.03) | -0.07 (0.07) | 0.18 (0.02) |
|  |  | EN | 0.60 (0.05) | 0.04 (0.04) | 0.24 (0.01) | 0.60 (0.06) | 0.05 (0.04) | 0.25 (0.06) | 0.60 (0.05) | 0.04 (0.03) | 0.23 (0.02) | 0.61 (0.05) | 0.01 (0.05) | 0.40 (0.00) |
| TMT-A | B | SVR | 0.76 (0.08) | -0.04 (0.07) | 0.12 (0.01) | 0.77 (0.09) | -0.09 (0.06) | -0.08 (0.03) | 0.78 (0.06) | -0.06 (0.07) | 0.12 (0.02) | 0.77 (0.05) | 0.01 (0.09) | 0.11 (0.02) |
|  |  | EN | 0.77 (0.07) | -0.02 (0.07) | 0.11 (0.04) | 0.78 (0.07) | -0.03 (0.04) | -0.08 (0.09) | 0.78 (0.05) | -0.02 (0.04) | 0.10 (0.05) | 0.78 (0.05) | 0.00 (0.08) | 0.03 (0.02) |
|  | B+D | SVR | 0.76 (0.08) | -0.03 (0.07) | 0.14 (0.01) | 0.77 (0.09) | -0.08 (0.06) | -0.03 (0.03) | 0.78 (0.06) | -0.05 (0.07) | 0.13 (0.02) | 0.77 (0.05) | 0.01 (0.09) | 0.11 (0.02) |
|  |  | EN | 0.69 (0.07) | 0.14 (0.06) | 0.40 (0.03) | 0.69 (0.07) | 0.15 (0.10) | 0.43 (0.04) | 0.70 (0.05) | 0.14 (0.07) | 0.40 (0.02) | 0.70 (0.05) | 0.14 (0.04) | 0.32 (0.02) |
| vWM | B | SVR | 0.59 (0.06) | 0.03 (0.08) | 0.22 (0.04) | 0.61 (0.05) | -0.03 (0.05) | 0.01 (0.03) | 0.60 (0.05) | 0.01 (0.07) | 0.23 (0.03) | 0.60 (0.04) | 0.03 (0.08) | 0.27 (0.05) |
|  |  | EN | 0.60 (0.05) | 0.02 (0.07) | 0.19 (0.06) | 0.61 (0.05) | -0.04 (0.05) | -0.10 (0.03) | 0.60 (0.05) | 0.01 (0.07) | 0.16 (0.09) | 0.60 (0.05) | 0.00 (0.09) | 0.20 (0.04) |
|  | B+D | SVR | 0.59 (0.05) | 0.05 (0.07) | 0.25 (0.03) | 0.61 (0.05) | -0.02 (0.05) | 0.05 (0.03) | 0.60 (0.05) | -0.07 (0.09) | 0.14 (0.07) | 0.60 (0.04) | 0.03 (0.08) | 0.28 (0.05) |
|  |  | EN | 0.56 (0.05) | 0.12 (0.05) | 0.37 (0.04) | 0.56 (0.05) | 0.13 (0.05) | 0.38 (0.03) | 0.57 (0.04) | 0.08 (0.05) | 0.31 (0.02) | 0.58 (0.05) | 0.06 (0.10) | 0.28 (0.09) |

*Note.* Standard deviation (SD) appears in parentheses.

*Supplementary Table 15.* Prediction results for all targets from structural connectivity (SC) in the younger sample.

| Younger | SC |  | DMN | | | FPN | | | FPNDMN | | | WHOLE | | |
| --- | --- | --- | --- | --- | --- | --- | --- | --- | --- | --- | --- | --- | --- | --- |
| Tar. | Mod. | Alg. | MAE | R^2^ | r | MAE | R^2^ | r | MAE | R^2^ | r | MAE | R^2^ | r |
| SF | B | SVR | 0.81 (0.05) | -0.05 (0.04) | 0.04 (0.02) | 0.81 (0.05) | -0.02 (0.03) | 0.04 (0.03) | 0.84 (0.06) | -0.10 (0.11) | 0.02 (0.06) | 0.84 (0.05) | -0.11 (0.08) | 0.03 (0.04) |
|  |  | EN | 0.82 (0.05) | -0.04 (0.07) | -0.05 (0.03) | 0.82 (0.05) | -0.03 (0.04) | 0.01 (0.08) | 0.82 (0.06) | -0.04 (0.04) | -0.07 (0.05) | 0.82 (0.04) | -0.04 (0.05) | -0.02 (0.05) |
|  | B+D | SVR | 0.82 (0.05) | -0.06 (0.08) | 0.03 (0.02) | 0.80 (0.05) | -0.02 (0.03) | 0.05 (0.05) | 0.84 (0.06) | -0.10 (0.10) | 0.03 (0.06) | 0.84 (0.05) | -0.11 (0.08) | 0.03 (0.04) |
|  |  | EN | 0.82 (0.05) | -0.04 (0.05) | -0.01 (0.06) | 0.81 (0.05) | -0.02 (0.02) | 0.04 (0.03) | 0.82 (0.07) | -0.04 (0.05) | -0.07 (0.05) | 0.82 (0.04) | -0.05 (0.04) | -0.01 (0.06) |
| PF | B | SVR | 0.83 (0.07) | -0.06 (0.08) | -0.04 (0.06) | 0.80 (0.08) | -0.02 (0.03) | 0.02 (0.06) | 0.83 (0.09) | -0.08 (0.09) | 0.03 (0.03) | 0.82 (0.08) | -0.07 (0.08) | 0.04 (0.02) |
|  |  | EN | 0.81 (0.07) | -0.02 (0.03) | -0.04 (0.07) | 0.81 (0.08) | -0.01 (0.03) | -0.02 (0.04) | 0.81 (0.09) | -0.02 (0.03) | -0.03 (0.04) | 0.82 (0.08) | -0.05 (0.08) | -0.07 (0.07) |
|  | B+D | SVR | 0.82 (0.07) | -0.05 (0.04) | -0.03 (0.06) | 0.81 (0.08) | -0.02 (0.03) | 0.02 (0.05) | 0.83 (0.09) | -0.08 (0.07) | 0.03 (0.03) | 0.82 (0.08) | -0.07 (0.08) | 0.04 (0.02) |
|  |  | EN | 0.81 (0.07) | -0.03 (0.04) | -0.04 (0.04) | 0.81 (0.08) | -0.02 (0.04) | -0.03 (0.03) | 0.81 (0.09) | -0.02 (0.02) | -0.05 (0.02) | 0.82 (0.07) | -0.05 (0.07) | -0.09 (0.02) |
| VF | B | SVR | 0.71 (0.06) | -0.06 (0.06) | 0.00 (0.05) | 0.70 (0.06) | -0.02 (0.04) | 0.08 (0.04) | 0.71 (0.06) | -0.11 (0.11) | 0.03 (0.01) | 0.71 (0.06) | -0.10 (0.07) | 0.02 (0.05) |
|  |  | EN | 0.70 (0.06) | -0.02 (0.02) | -0.04 (0.01) | 0.69 (0.06) | -0.02 (0.02) | 0.05 (0.03) | 0.71 (0.07) | -0.06 (0.12) | -0.09 (0.04) | 0.70 (0.06) | -0.05 (0.05) | -0.07 (0.02) |
|  | B+D | SVR | 0.70 (0.06) | -0.05 (0.05) | 0.02 (0.01) | 0.69 (0.06) | -0.02 (0.04) | 0.09 (0.03) | 0.71 (0.06) | -0.11 (0.11) | 0.03 (0.01) | 0.71 (0.06) | -0.10 (0.07) | 0.02 (0.05) |
|  |  | EN | 0.69 (0.06) | -0.02 (0.03) | -0.01 (0.07) | 0.70 (0.06) | -0.02 (0.03) | 0.06 (0.03) | 0.71 (0.06) | -0.07 (0.12) | -0.07 (0.04) | 0.70 (0.06) | -0.06 (0.07) | -0.08 (0.01) |
| VOC | B | SVR | 0.79 (0.07) | -0.03 (0.05) | 0.07 (0.03) | 0.78 (0.08) | -0.03 (0.06) | 0.06 (0.08) | 0.80 (0.08) | -0.07 (0.09) | 0.08 (0.02) | 0.81 (0.08) | -0.07 (0.07) | 0.04 (0.01) |
|  |  | EN | 0.80 (0.07) | -0.03 (0.04) | -0.03 (0.02) | 0.80 (0.07) | -0.03 (0.04) | 0.01 (0.06) | 0.81 (0.06) | -0.06 (0.06) | -0.04 (0.02) | 0.81 (0.06) | -0.05 (0.08) | 0.02 (0.05) |
|  | B+D | SVR | 0.79 (0.07) | -0.03 (0.07) | 0.09 (0.04) | 0.78 (0.08) | -0.02 (0.06) | 0.10 (0.08) | 0.79 (0.08) | -0.06 (0.09) | 0.09 (0.02) | 0.81 (0.08) | -0.07 (0.07) | 0.04 (0.01) |
|  |  | EN | 0.72 (0.06) | 0.12 (0.05) | 0.37 (0.04) | 0.72 (0.07) | 0.12 (0.06) | 0.38 (0.03) | 0.74 (0.06) | 0.09 (0.05) | 0.33 (0.02) | 0.73 (0.07) | 0.12 (0.05) | 0.38 (0.07) |
| VER | B | SVR | 0.64 (0.05) | -0.06 (0.06) | 0.00 (0.04) | 0.62 (0.04) | -0.02 (0.03) | 0.06 (0.07) | 0.64 (0.05) | -0.09 (0.13) | 0.06 (0.03) | 0.65 (0.06) | -0.10 (0.07) | 0.00 (0.05) |
|  |  | EN | 0.62 (0.05) | -0.02 (0.03) | -0.01 (0.00) | 0.62 (0.04) | -0.02 (0.03) | 0.02 (0.03) | 0.63 (0.06) | -0.07 (0.14) | -0.11 (0.06) | 0.62 (0.06) | -0.02 (0.03) | -0.01 (0.04) |
|  | B+D | SVR | 0.63 (0.05) | -0.05 (0.06) | 0.02 (0.04) | 0.63 (0.05) | -0.01 (0.05) | 0.11 (0.02) | 0.64 (0.05) | -0.09 (0.12) | 0.06 (0.02) | 0.65 (0.06) | -0.10 (0.07) | 0.00 (0.05) |
|  |  | EN | 0.60 (0.05) | 0.06 (0.03) | 0.29 (0.02) | 0.60 (0.05) | 0.04 (0.03) | 0.23 (0.02) | 0.60 (0.05) | 0.03 (0.04) | 0.24 (0.04) | 0.61 (0.06) | 0.02 (0.04) | 0.17 (0.02) |
| TMT-A | B | SVR | 0.72 (0.08) | 0.02 (0.08) | 0.25 (0.03) | 0.74 (0.08) | -0.01 (0.09) | 0.20 (0.02) | 0.73 (0.07) | 0.04 (0.08) | 0.27 (0.04) | 0.74 (0.07) | 0.06 (0.10) | 0.28 (0.03) |
|  |  | EN | 0.76 (0.07) | 0.01 (0.08) | 0.16 (0.05) | 0.76 (0.07) | 0.02 (0.08) | 0.20 (0.06) | 0.75 (0.07) | 0.05 (0.07) | 0.25 (0.00) | 0.75 (0.07) | 0.03 (0.10) | 0.24 (0.03) |
|  | B+D | SVR | 0.72 (0.08) | 0.02 (0.07) | 0.26 (0.03) | 0.73 (0.07) | 0.00 (0.08) | 0.21 (0.03) | 0.72 (0.07) | 0.05 (0.08) | 0.27 (0.04) | 0.74 (0.07) | 0.06 (0.10) | 0.28 (0.03) |
|  |  | EN | 0.70 (0.05) | 0.12 (0.08) | 0.38 (0.03) | 0.70 (0.06) | 0.14 (0.07) | 0.40 (0.01) | 0.70 (0.07) | 0.12 (0.08) | 0.38 (0.06) | 0.71 (0.06) | 0.10 (0.08) | 0.34 (0.03) |
| vWM | B | SVR | 0.59 (0.05) | 0.03 (0.06) | 0.24 (0.03) | 0.59 (0.06) | 0.01 (0.05) | 0.17 (0.01) | 0.59 (0.04) | 0.04 (0.09) | 0.27 (0.05) | 0.59 (0.07) | 0.01 (0.10) | 0.24 (0.04) |
|  |  | EN | 0.59 (0.06) | 0.04 (0.05) | 0.23 (0.02) | 0.60 (0.05) | 0.00 (0.05) | 0.13 (0.06) | 0.58 (0.04) | 0.07 (0.05) | 0.28 (0.03) | 0.59 (0.06) | 0.01 (0.09) | 0.21 (0.06) |
|  | B+D | SVR | 0.59 (0.05) | 0.04 (0.06) | 0.24 (0.03) | 0.59 (0.06) | 0.02 (0.05) | 0.20 (0.00) | 0.59 (0.04) | 0.04 (0.09) | 0.27 (0.05) | 0.59 (0.07) | 0.01 (0.10) | 0.24 (0.04) |
|  |  | EN | 0.57 (0.06) | 0.11 (0.05) | 0.36 (0.04) | 0.56 (0.05) | 0.12 (0.05) | 0.37 (0.02) | 0.58 (0.04) | 0.09 (0.05) | 0.33 (0.05) | 0.58 (0.07) | 0.05 (0.07) | 0.28 (0.05) |

*Note.* Standard deviation (SD) appears in parentheses.

*Supplementary Table 16.* Prediction results of all targets from demographics, i.e. age, education and sex, across samples.

| Demos |  | SVR | | | EN | | |
| --- | --- | --- | --- | --- | --- | --- | --- |
| Mod. | Tar. | MAE | R^2^ | r | MAE | R^2^ | r |
| Total | SF | 0.75 (0.04) | 0.10 (0.05) | 0.32 (0.01) | 0.75 (0.04) | 0.10 (0.04) | 0.33 (0.01) |
|  | PF | 0.78 (0.04) | 0.06 (0.04) | 0.25 (0.01) | 0.78 (0.04) | 0.06 (0.04) | 0.26 (0.01) |
|  | VF | 0.65 (0.04) | 0.11 (0.05) | 0.34 (0.00) | 0.65 (0.03) | 0.11 (0.05) | 0.34 (0.00) |
|  | VOC | 0.68 (0.04) | 0.20 (0.06) | 0.45 (0.00) | 0.68 (0.04) | 0.20 (0.05) | 0.46 (0.00) |
|  | VER | 0.56 (0.03) | 0.18 (0.06) | 0.43 (0.00) | 0.56 (0.03) | 0.18 (0.05) | 0.43 (0.00) |
|  | TMT-A | 0.63 (0.04) | 0.32 (0.05) | 0.58 (0.00) | 0.63 (0.04) | 0.33 (0.05) | 0.58 (0.00) |
|  | vWM | 0.52 (0.03) | 0.35 (0.06) | 0.59 (0.00) | 0.52 (0.03) | 0.35 (0.05) | 0.59 (0.00) |
| Older | SF | 0.76 (0.06) | 0.07 (0.06) | 0.28 (0.02) | 0.76 (0.06) | 0.07 (0.05) | 0.28 (0.01) |
|  | PF | 0.76 (0.06) | 0.08 (0.07) | 0.32 (0.01) | 0.76 (0.06) | 0.10 (0.06) | 0.33 (0.01) |
|  | VF | 0.64  (0.04) | 0.11 (0.08) | 0.36 (0.01) | 0.64 (0.04) | 0.12 (0.06) | 0.36 (0.01) |
|  | VOC | 0.68 (0.05) | 0.21 (0.08) | 0.47 (0.01) | 0.68 (0.05) | 0.21 (0.07) | 0.47 (0.01) |
|  | VER | 0.55 (0.04) | 0.21 (0.08) | 0.46 (0.01) | 0.55 (0.04) | 0.21 (0.07) | 0.47 (0.01) |
|  | TMT-A | 0.74 (0.06) | 0.12 (0.08) | 0.36 (0.01) | 0.74 (0.05) | 0.13 (0.07) | 0.37 (0.01) |
|  | vWM | 0.57 (0.04) | 0.16 (0.07) | 0.41 (0.01) | 0.57 (0.04) | 0.16 (0.07) | 0.42 (0.01) |
| Younger | SF | 0.75 (0.04) | 0.10 (0.05) | 0.32 (0.01) | 0.75 (0.04) | 0.02 (0.04) | 0.33 (0.01) |
|  | PF | 0.78 (0.04) | 0.06 (0.04) | 0.25 (0.01) | 0.78 (0.04) | 0.06 (0.04) | 0.26 (0.01) |
|  | VF | 0.65 (0.04) | 0.11 (0.05) | 0.34 (0.00) | 0.65 (0.03) | 0.11 (0.05) | 0.34 (0.00) |
|  | VOC | 0.68 (0.04) | 0.20 (0.06) | 0.45 (0.00) | 0.68 (0.04) | 0.20 (0.05) | 0.46 (0.00) |
|  | VER | 0.56 (0.03) | 0.18 (0.06) | 0.43 (0.00) | 0.56 (0.03) | 0.18 (0.05) | 0.43 (0.00) |
|  | TMT-A | 0.63 (0.04) | 0.32 (0.05) | 0.58 (0.00) | 0.63 (0.04) | 0.33 (0.05) | 0.58 (0.00) |
|  | vWM | 0.52 (0.03) | 0.35 (0.06) | 0.59 (0.00) | 0.52 (0.03) | 0.35 (0.05) | 0.59 (0.00) |

*Note.* Standard deviation (SD) appears in parentheses.

**Validation analyses**

*Supplementary Table 17.* Prediction results for age from brain connectivity data across samples.

| Age |  |  | EN | | | SVR | | |
| --- | --- | --- | --- | --- | --- | --- | --- | --- |
| Sample | Mod. | FSet | MAE | R^2^ | r | MAE | R^2^ | r |
| Total | FC | DMN | 8.83 (0.36) | 0.31 (0.05) | 0.56 (0.02) | 9.02 (0.36) | 0.25 (0.05) | 0.52 (0.02 |
|  |  | FPN | 8.98 (0.40) | 0.27 (0.05) | 0.53 (0.00) | 8.93 (0.32) | 0.23 (0.04) | 0.50 (0.01) |
|  |  | FPNDMN | 8.16 (0.41) | 0.40 (0.05) | 0.64 (0.02) | 8.21 (0.44) | 0.37 (0.05) | 0.62 (0.02) |
|  |  | WHOLE | 7.51 (0.58) | 0.48 (0.05) | 0.70 (0.01) | 7.40 (0.50) | 0.49 (0.04) | 0.71 (0.01) |
|  | SC | DMN | 6.68 (0.51) | 0.60 (0.05) | 0.78 (0.01) | 6.84 (0.54) | 0.57 (0.05) | 0.76 (0.00) |
|  |  | FPN | 7.36 (0.32) | 0.52 (0.03) | 0.73 (0.00) | 7.28 (0.33) | 0.51 (0.03) | 0.72 (0.01) |
|  |  | FPNDMN | 6.53 (0.46) | 0.62 (0.04) | 0.79 (0.01) | 6.46 (0.45) | 0.62 (0.04) | 0.79 (0.01) |
|  |  | WHOLE | 5.45 (0.58) | 0.48 (0.05) | 0.70 (0.01) | 5.50 (0.36) | 0.72 (0.03) | 0.86 (0.00) |
| Older | FC | DMN | 4.51 (0.39) | 0.07 (0.05) | 0.28 (0.04) | 4.58 (0.37) | 0.02 (0.04) | 0.22 (0.04) |
|  |  | FPN | 4.57 (0.36) | 0.04 (0.05) | 0.23 (0.02) | 4.61 (0.34) | 0.01 (0.04) | 0.19 (0.00) |
|  |  | FPNDMN | 4.49 (0.34) | 0.07 (0.06) | 0.29 (0.02) | 4.54 (0.33) | 0.03 (0.06) | 0.25 (0.00) |
|  |  | WHOLE | 4.50 (0.27) | 0.05 (0.07) | 0.29 (0.01) | 4.40 (0.30) | 0.07 (0.07) | 0.31 (0.04) |
|  | SC | DMN | 4.01 (0.35) | 0.23 (0.08) | 0.49 (0.04) | 3.98 (0.34) | 0.22 (0.07) | 0.49 (0.01) |
|  |  | FPN | 4.03 (0.33) | 0.22 (0.10) | 0.49 (0.04) | 4.05 (0.31) | 0.21 (0.11) | 0.47 (0.04) |
|  |  | FPNDMN | 3.88 (0.30) | 0.26 (0.08) | 0.52 (0.01) | 3.93 (0.33) | 0.24 (0.08) | 0.51 (0.02) |
|  |  | WHOLE | 3.68 (0.30) | 0.34 (0.10) | 0.59 (0.02) | 3.61 (0.30) | 0.37 (0.09) | 0.61 (0.02) |
| Younger | FC | DMN | 8.77 (0.48) | 0.04 (0.12) | 0.28 (0.06) | 8.65 (0.50) | 0.00 (0.09) | 0.26 (0.04) |
|  |  | FPN | 8.86 (0.47) | 0.03 (0.09) | 0.26 (0.01) | 8.86 (0.47) | -0.04 (0.09) | 0.20 (0.06) |
|  |  | FPNDMN | 8.03 (0.50) | 0.17 (0.08) | 0.44 (0.05) | 8.04 (0.47) | 0.15 (0.08) | 0.42 (0.04) |
|  |  | WHOLE | 7.82 (0.73) | 0.21 (0.13) | 0.29 (0.01) | 7.82 (0.66) | 0.19 (0.10) | 0.31 (0.04) |
|  | SC | DMN | 6.73 (0.53) | 0.39 (0.07) | 0.63 (0.02) | 6.68 (0.59) | 0.36 (0.09) | 0.61 (0.01) |
|  |  | FPN | 7.18 (0.64) | 0.33 (0.07) | 0.58 (0.01 | 7.07 (0.74) | 0.33 (0.09) | 0.58 (0.02 |
|  |  | FPNDMN | 6.43 (0.57) | 0.43 (0.08) | 0.66 (0.00) | 6.38 (0.52) | 0.42 (0.07) | 0.66 (0.01) |
|  |  | WHOLE | 6.25 (0.51) | 0.45 (0.05) | 0.69 (0.01) | 6.25 (0.55) | 0.45 (0.06) | 0.69 (0.02) |

*Note.* Standard deviation (SD) appears in parentheses.

*Supplementary Table 18.* Sex classification results from brain connectivity data across samples.

|  |  |  | Acc. | |
| --- | --- | --- | --- | --- |
| Mod. | FSet | Tar. | Ridge | SVC |
| Total | FC | DMN | 68.87 (3.26) | 67.73 (3.09) |
|  |  | FPN | 65.75 (2.55) | 64.07 (3.45) |
|  |  | FPNDMN | 72.63 (3.94) | 70.85 (3.16) |
|  |  | WHOLE | 79.58 (3.00) | 75.14 (4.20) |
|  | SC | DMN | 80.73 (3.33) | 78.58 (3.52) |
|  |  | FPN | 73.98 (2.24) | 71.55 (3.94) |
|  |  | FPNDMN | 80.64 (2.89) | 79.00 (2.68) |
|  |  | WHOLE | 86.70 (2.86) | 82.68 (2.45) |
| Older | FC | DMN | 69.36 (3.68) | 67.64 (4.32) |
|  |  | FPN | 65.90 (4.66) | 61.63 (4.79) |
|  |  | FPNDMN | 72.41 (3.74) | 66.70 (3.97) |
|  |  | WHOLE | 74.93 (5.00) | 68.61 (4.96) |
|  | SC | DMN | 77.49 (4.35) | 72.47 (4.22) |
|  |  | FPN | 71.63 (4.96) | 70.30 (3.93) |
|  |  | FPNDMN | 78.77 (3.89) | 70.55 (4.28) |
|  |  | WHOLE | 85.17 (3.27) | 82.12 (2.69) |
| Younger | FC | DMN | 70.04 (3.99) | 66.23 (5.22) |
|  |  | FPN | 60.64 (4.89) | 57.81 (6.59) |
|  |  | FPNDMN | 70.85 (6.00) | 67.45 (5.07) |
|  |  | WHOLE | 72.54 (4.69) | 70.04 (6.00) |
|  | SC | DMN | 80.84 (3.62) | 75.95 (5.10) |
|  |  | FPN | 73.83 (4.60) | 69.72 (6.17) |
|  |  | FPNDMN | 82.96 (3.46) | 78.90 (4.47) |
|  |  | WHOLE | 82.91 (5.25) | 76.99 (3.58) |

*Note.* Standard deviation (SD) appears in parentheses.

*Supplementary Table 19.* Extreme group classification results for semantic fluency from brain connectivity data across samples.

|  |  |  | Acc. | |
| --- | --- | --- | --- | --- |
| Sample | FSet | Mod. | Ridge | SVC |
| Total | FC | DMN | 61.23 (4.72) | 59.27 (3.69) |
|  |  | FPN | 56.25 (6.01) | 54.58 (4.93) |
|  |  | FPNDMN | 57.76 (5.11) | 55.53 (6.49) |
|  |  | WHOLE | 58.88 (4.94) | 59.94 (4.92) |
|  | SC | DMN | 62.24 (4.75) | 60.95 (5.56) |
|  |  | FPN | 60.18 (5.54) | 57.15 (5.12) |
|  |  | FPNDMN | 62.19 (4.2) | 58.45 (4.37) |
|  |  | WHOLE | 63.58 (5.86) | 58.32 (5.47) |
| Older | FC | DMN | 58.23 (7.04) | 50.87 (5.96) |
|  |  | FPN | 55.78 (5.56) | 47.63 (7.53) |
|  |  | FPNDMN | 55.8 (6.03) | 55.57 (7.8) |
|  |  | WHOLE | 58.83 (6.05) | 52.73 (6.8) |
|  | SC | DMN | 57.46 (5.84) | 52.66 (7.84) |
|  |  | FPN | 51.97 (6.16) | 49.92 (6.84) |
|  |  | FPNDMN | 55.99 (6.81) | 51.76 (7.3) |
|  |  | WHOLE | 54.12 (6.83) | 48.83 (6.03) |
| Younger | FC | DMN | 52.81 (6.68) | 51.00 (7.75) |
|  |  | FPN | 53.48 (6.91) | 50.00 (6.36) |
|  |  | FPNDMN | 54.5 (7.94) | 50.16 (8.7) |
|  |  | WHOLE | 55.91 (8.1) | 51.24 (8.69) |
|  | SC | DMN | 54.08 (9.86) | 53.84 (6.74) |
|  |  | FPN | 57.53 (6.54) | 56.27 (8.59) |
|  |  | FPNDMN | 55.48 (7.24) | 53.47 (6.96) |
|  |  | WHOLE | 56.52 (8.48) | 56.69 (7.49) |

*Note.* Standard deviation (SD) appears in parentheses.

*Supplementary Table 20.* Extreme group classification results for phonematic fluency from brain connectivity data across samples.

|  |  |  | Acc. | |
| --- | --- | --- | --- | --- |
| Sample | FSet | Mod. | Ridge | SVC |
| Total | FC | DMN | 54.53 (6.34) | 52.23 (5.39) |
|  |  | FPN | 56.36 (4.77) | 54.46 (5.81) |
|  |  | FPNDMN | 55.30 (5.89) | 54.41 (5.64) |
|  |  | WHOLE | 56.93 (4.94) | 53.86 (3.74) |
|  | SC | DMN | 58.27 (4.92) | 54.58 (5.68) |
|  |  | FPN | 59.27 (5.60) | 53.29 (3.47) |
|  |  | FPNDMN | 59.44 (4.74) | 57.09 (6.35) |
|  |  | WHOLE | 63.30 (5.68) | 56.10 (4.02) |
| Older | FC | DMN | 51.58 (5.55) | 51.29 (6.81) |
|  |  | FPN | 56.36 (5.41) | 49.72 (7.26) |
|  |  | FPNDMN | 49.73 (6.33) | 48.54 (5.63) |
|  |  | WHOLE | 57.44 (8.88) | 51.95 (7.07) |
|  | SC | DMN | 50 .00  (7.09) | 46.55 (7.98) |
|  |  | FPN | 49.31 (7.07) | 47.65 (7.80) |
|  |  | FPNDMN | 48.71 (7.20) | 52.15 (7.40) |
|  |  | WHOLE | 56.85 (7.85) | 57.25 (6.78) |
| Younger | FC | DMN | 44.98 (6.96) | 47.06  (6.00) |
|  |  | FPN | 51.65 (7.49) | 50.61 (6.43) |
|  |  | FPNDMN | 44.75 (7.81) | 47.84 (4.69) |
|  |  | WHOLE | 43.95 (7.26) | 45.1  (9.6) |
|  | SC | DMN | 44.98 (7.81) | 47.06 (4.69) |
|  |  | FPN | 51.65 (7.26) | 50.61 (9.6) |
|  |  | FPNDMN | 44.75  (6.000) | 47.84 (7.94) |
|  |  | WHOLE | 43.95 (6.43) | 45.1 (9.09) |

*Note.* Standard deviation (SD) appears in parentheses.

*Supplementary Table 21.* Extreme group classification results for verbal fluency from brain connectivity data across samples.

|  |  |  | Acc. | |
| --- | --- | --- | --- | --- |
| Sample | FSet | Mod. | Ridge | SVC |
| Total | FC | DMN | 58.11 (5.91) | 56.43 (4.84) |
|  |  | FPN | 59.95 (5.09) | 57.09 (5.49) |
|  |  | FPNDMN | 59.33 (5.25) | 55.41 (6.24) |
|  |  | WHOLE | 60.23 (3.82) | 58.17 (5.35) |
|  | SC | DMN | 59.62 (4.58) | 57.15 (5.25) |
|  |  | FPN | 60.17 (3.95) | 57.59 (6.51) |
|  |  | FPNDMN | 60.57 (4.84) | 58.44 (4.19) |
|  |  | WHOLE | 64.14 (5.69) | 60.28 (5.34) |
| Older | FC | DMN | 57.35 (5.84) | 52.63 (6.84) |
|  |  | FPN | 56.34 (7.28) | 54.81 (6.50) |
|  |  | FPNDMN | 54.59 (5.61) | 54.53 (5.48) |
|  |  | WHOLE | 57.44 (6.68) | 52.63 (6.62) |
|  | SC | DMN | 51.77 (7.10) | 52.06 (6.03) |
|  |  | FPN | 54.81 (4.8) | 47.65 (6.08) |
|  |  | FPNDMN | 50.99 (6.26) | 49.11 (8.19) |
|  |  | WHOLE | 56.17 (5.42) | 55.10 (6.87) |
| Younger | FC | DMN | 47.2 (7.94) | 50.53 (9.12) |
|  |  | FPN | 54.22 (9.09) | 51.29 (8.44) |
|  |  | FPNDMN | 51.16 (8.64) | 54.11 (8.40) |
|  |  | WHOLE | 54.1 (7.64) | 52.19 (8.14) |
|  | SC | DMN | 47.20 (8.64) | 50.53 (8.4) |
|  |  | FPN | 54.22 (7.64) | 51.29 (8.14) |
|  |  | FPNDMN | 51.16 (9.12) | 54.11 (7.63) |
|  |  | WHOLE | 54.1 (8.44) | 52.19 (8.33) |

*Note.* Standard deviation (SD) appears in parentheses.

*Supplementary Table 22.* Extreme group classification results for vocabulary from brain connectivity data across samples.

|  |  |  | Acc. | |
| --- | --- | --- | --- | --- |
| Sample | FSet | Mod. | Ridge | SVC |
| Total | FC | DMN | 54.7 (5.56) | 52.75 (4.68) |
|  |  | FPN | 57.55 (5.6) | 57.88 (5.78) |
|  |  | FPNDMN | 55.09 (6.78) | 54.93 (4.45) |
|  |  | WHOLE | 56.26 (5.36) | 53.06 (6.46) |
|  | SC | DMN | 54.97 (4.34) | 55.7 (5.55) |
|  |  | FPN | 53.68 (4.9) | 53.8 (4.41) |
|  |  | FPNDMN | 54.75 (5.16) | 52.9 (6.15) |
|  |  | WHOLE | 55.64 (4.49) | 52.79 (4.61) |
| Older | FC | DMN | 58.35 (7.84) | 50.00 (5.74) |
|  |  | FPN | 55.5 (5.49) | 53.93 (5.97) |
|  |  | FPNDMN | 58.63 (7.73) | 51.27 (7.61) |
|  |  | WHOLE | 60.5 (5.59) | 56.19 (6.74) |
|  | SC | DMN | 59.3 (6.04) | 56.36 (6.80) |
|  |  | FPN | 52.24 (5.69) | 53.44 (5.84) |
|  |  | FPNDMN | 59.22 (5.98) | 56.47 (6.35) |
|  |  | WHOLE | 56.84 (7.03) | 55.80 (7.99) |
| Younger | FC | DMN | 48.88 (7.63) | 46.81 (4.11) |
|  |  | FPN | 54.88 (8.33) | 60.23 (6.60) |
|  |  | FPNDMN | 51.05 (8.90) | 50.79 (8.38) |
|  |  | WHOLE | 52.42 (8.66) | 52.70 (8.17) |
|  | SC | DMN | 48.88 (8.90) | 46.81 (8.38) |
|  |  | FPN | 54.88 (8.66) | 60.23 (8.17) |
|  |  | FPNDMN | 51.05 (4.11) | 50.79 (5.02) |
|  |  | WHOLE | 52.42 (6.60) | 52.70 (8.23) |

*Note.* Standard deviation (SD) appears in parentheses.

*Supplementary Table 23.* Extreme group classification results for verbal abilities from brain connectivity data across samples.

|  |  |  | Acc. | |
| --- | --- | --- | --- | --- |
| Sample | FSet | Mod. | Ridge | SVC |
| Total | FC | DMN | 59.71 (4.19) | 57.19 (5.51) |
|  |  | FPN | 56.15 (4.92) | 55.87 (5.07) |
|  |  | FPNDMN | 57.67 (4.97) | 56.37 (5.31) |
|  |  | WHOLE | 59.33 (6.73) | 56.27 (6.65) |
|  | SC | DMN | 60.00 (5.90) | 55.59 (4.79) |
|  |  | FPN | 61.23 (4.44) | 56.81 (5.19) |
|  |  | FPNDMN | 62.52 (5.91) | 55.86 (4.14) |
|  |  | WHOLE | 64.65 (4.72) | 59.45 (5.75) |
| Older | FC | DMN | 56.67 (9.26) | 55.29 (6.86) |
|  |  | FPN | 59.5 (6.85) | 57.74 (6.06) |
|  |  | FPNDMN | 57.84 (8.45) | 53.21 (6.94) |
|  |  | WHOLE | 61.38 (6.18) | 61.08 (7.36) |
|  | SC | DMN | 55.68 (6.35) | 55.29 (5.55) |
|  |  | FPN | 57.06 (7.34) | 53.36 (5.94) |
|  |  | FPNDMN | 56.46 (6.17) | 51.96  (0) |
|  |  | WHOLE | 60.6 (7.90) | 53.54  (0) |
| Younger | FC | DMN | 46.79 (5.02) | 48.20 (5.86) |
|  |  | FPN | 46.31 (8.23) | 51.15 (6.7) |
|  |  | FPNDMN | 44.35 (8.50) | 47.07 (4.75) |
|  |  | WHOLE | 53.23 (8.43) | 48.97 (7.18) |
|  | SC | DMN | 46.79 (8.50) | 48.20 (4.75) |
|  |  | FPN | 46.31 (8.43) | 51.15 (7.18) |
|  |  | FPNDMN | 44.35 (5.86) | 47.07  (0) |
|  |  | WHOLE | 53.23 (6.70) | 48.97  (0) |

*Note.* Standard deviation (SD) appears in parentheses.

*Supplementary Table 24.*Prediction results (Coefficient of Determination [R^2^]) for verbal and non-verbal cognitive functions from FC in the total sample using the 800-node Schaefer parcellation.

| FC 800 Nodes |  | Total | |
| --- | --- | --- | --- |
| Mod. | Tar. | EN | SVR |
| DMN | SF | -0.03 (0.05) | -0.14 (0.09) |
|  | PF | -0.02 (0.03) | -0.2 (0.11) |
|  | VF | -0.03 (0.04) | -0.18 (0.11) |
|  | VOC | -0.02 (0.02) | -0.14 (0.08) |
|  | VER | -0.03 (0.03) | -0.2 (0.09) |
|  | TMT-A | -0.03 (0.05) | -0.07 (0.09) |
|  | vWM | -0.01 (0.06) | -0.07 (0.08) |
| FPN | SF | 0.03 (0.02) | -0.05 (0.04) |
|  | PF | -0.01 (0.02) | -0.05 (0.05) |
|  | VF | 0.03 (0.02) | -0.06 (0.05) |
|  | VOC | 0  (0.02) | -0.04 (0.05) |
|  | VER | 0.01 (0.02) | -0.05 (0.05) |
|  | TMT-A | 0.32 (0.05) | 0.09 (0.06) |
|  | vWM | 0.34 (0.05) | 0.15 (0.04) |
| FPNDMN | SF | 0.01 (0.02) | -0.24 (0.11) |
|  | PF | -0.04 (0.04) | -0.21 (0.1) |
|  | VF | 0  (0.03) | -0.17 (0.1) |
|  | VOC | -0.03 (0.05) | -0.18 (0.1) |
|  | VER | 0  (0.04) | 0.05 (0.09) |
|  | TMT-A | 0.11 (0.07) | 0.09 (0.06) |
|  | vWM | 0.16 (0.04) | -0.24 (0.11) |

*Note.* Standard deviation (SD) appears in parentheses.

*Supplementary Table 25.* Prediction results (Coefficient of Determination [R^2^]) for verbal and non-verbal cognitive functions from SC in the total sample using the 800-node Schaefer parcellation.

| SC 800 Nodes |  | Total | |
| --- | --- | --- | --- |
| Mod. | Tar. | EN | SVR |
| DMN | SF | 0.05  (0.03) | 0.01 (0.06) |
|  | PF | 0  (0.02) | -0.06 (0.06) |
|  | VF | 0.03  (0.02) | -0.01 (0.05) |
|  | VOC | -0.01 (0.01) | -0.11 (0.1) |
|  | VER | 0.03  (0.03) | -0.02 (0.06) |
|  | TMT-A | 0.17  (0.05) | 0.12 (0.07) |
|  | vWM | 0.17  (0.05) | 0.15 (0.06) |
| FPN | SF | 0.03  (0.03) | 0.02 (0.06) |
|  | PF | 0  (0.02) | -0.03 (0.04) |
|  | VF | 0.03  (0.03) | 0  (0.03) |
|  | VOC | -0.01 (0.02) | -0.04 (0.03) |
|  | VER | 0.02  (0.02) | -0.01 (0.04) |
|  | TMT-A | 0.31  (0.04) | 0.16 (0.06) |
|  | vWM | 0.34  (0.05) | 0.11 (0.05) |
| FPNDMN | SF | 0.05  (0.02) | 0  (0.06) |
|  | PF | 0  (0.03) | -0.08 (0.16) |
|  | VF | 0.05  (0.03) | 0.01 (0.04) |
|  | VOC | -0.01 (0.02) | -0.13 (0.08) |
|  | VER | 0.03  (0.03) | -0.02 (0.07) |
|  | TMT-A | 0  (0.91) | 0.15 (0.08) |
|  | vWM | 0.18  (0.04) | 0.16 (0.05) |

*Note.* Standard deviation (SD) appears in parentheses.

*Supplementary Table 26.* Prediction results (Coefficient of Determination [R^2^]) for verbal and non-verbal cognitive functions from FC from the left hemisphere across samples, i.e. total, older and younger.

| FC Left |  | Total | | Older | | Younger | |
| --- | --- | --- | --- | --- | --- | --- | --- |
| Mod. | Tar. | EN | SVR | EN | SVR | EN | SVR |
| DMN | SF | 0.01 (0.02) | 0  (0.03) | -0.01 (0.01) | -0.03 (0.03) | -0.02 (0.03) | -0.03 (0.07) |
|  | PF | -0.02 (0.02) | -0.02 (0.03) | -0.02 (0.02) | -0.04 (0.03) | -0.03 (0.04) | -0.03 (0.04) |
|  | VF | 0  (0.03) | 0  (0.04) | -0.02 (0.02) | -0.03 (0.03) | -0.03 (0.03) | -0.04 (0.06) |
|  | VOC | -0.01 (0.03) | -0.02 (0.04) | -0.02 (0.03) | -0.03 (0.03) | -0.02 (0.02) | -0.05 (0.05) |
|  | VER | 0  (0.03) | 0.01 (0.04) | -0.01 (0.03) | -0.03 (0.04) | -0.02 (0.03) | -0.04 (0.05) |
|  | TMT-A | 0.05 (0.05) | 0.01 (0.06) | -0.03 (0.04) | -0.06 (0.05) | -0.04 (0.05) | -0.05 (0.08) |
|  | vWM | 0.1  (0.05) | 0.07 (0.06) | -0.02 (0.03) | -0.02 (0.04) | 0  (0.07) | 0  (0.08) |
| FPN | SF | 0.01 (0.02) | 0  (0.02) | -0.02 (0.02) | -0.01 (0.02) | -0.02 (0.03) | -0.03 (0.03) |
|  | PF | -0.02 (0.02) | -0.02 (0.03) | -0.02 (0.02) | -0.03 (0.03) | -0.03 (0.03) | -0.03 (0.03) |
|  | VF | 0.01 (0.02) | 0  (0.02) | -0.02 (0.03) | -0.02 (0.05) | -0.04 (0.04) | -0.03 (0.03) |
|  | VOC | -0.01 (0.02) | -0.01 (0.03) | -0.03 (0.03) | -0.02 (0.03) | -0.04 (0.06) | -0.02 (0.04) |
|  | VER | 0  (0.02) | 0  (0.02) | -0.02 (0.04) | -0.02 (0.05) | -0.05 (0.05) | -0.06 (0.07) |
|  | TMT-A | 0.02 (0.06) | -0.01 (0.06) | -0.04 (0.05) | -0.05 (0.06) | -0.03 (0.03) | -0.08 (0.08) |
|  | vWM | 0.03 (0.03) | 0.02 (0.03) | -0.02 (0.02) | -0.01 (0.02) | -0.03 (0.04) | -0.03 (0.04) |
| FPNDMN | SF | 0  (0.02) | 0  (0.03) | -0.01 (0.02) | -0.02 (0.03) | -0.03 (0.03) | -0.03 (0.03) |
|  | PF | 0  (0.03) | 0  (0.04) | -0.02 (0.04) | -0.03 (0.04) | -0.02 (0.03) | -0.03 (0.04) |
|  | VF | 0  (0.02) | 0  (0.04) | -0.01 (0.02) | -0.01 (0.03) | -0.02 (0.03) | -0.04 (0.04) |
|  | VOC | 0  (0.02) | -0.02 (0.04) | -0.02 (0.02) | -0.03 (0.03) | -0.03 (0.04) | -0.05 (0.08) |
|  | VER | 0  (0.04) | 0.01 (0.04) | 0  (0.03) | -0.03 (0.06) | -0.02 (0.02) | -0.05 (0.03) |
|  | TMT-A | 0.08 (0.04) | 0.02 (0.05) | -0.04 (0.03) | -0.05 (0.05) | -0.03 (0.04) | -0.08 (0.08) |
|  | vWM | 0.11 (0.04) | 0.11 (0.05) | -0.01 (0.04) | 0  (0.04) | 0.01 (0.06) | -0.01 (0.09) |
| WHOLE | SF | 0.02 (0.03) | -0.12 (0.07) | -0.02 (0.02) | -0.13 (0.09) | -0.04 (0.04) | -0.17 (0.11) |
|  | PF | -0.02 (0.03) | -0.14 (0.08) | -0.03 (0.04) | -0.15 (0.07) | -0.02 (0.03) | -0.19 (0.09) |
|  | VF | 0.01 (0.04) | -0.14 (0.09) | -0.02 (0.02) | -0.13 (0.07) | -0.05 (0.04) | -0.18 (0.08) |
|  | VOC | -0.01 (0.03) | -0.11 (0.07) | -0.03 (0.04) | -0.16 (0.13) | -0.04 (0.05) | -0.1 (0.09) |
|  | VER | 0  (0.04) | -0.12 (0.09) | -0.01 (0.04) | -0.1 (0.08) | -0.04 (0.05) | -0.15 (0.1) |
|  | TMT-A | 0.11 (0.05) | 0.03 (0.08) | -0.03 (0.06) | -0.11 (0.07) | -0.03 (0.05) | -0.08 (0.11) |
|  | vWM | 0.18 (0.04) | 0.13 (0.05) | 0.01 (0.03) | -0.11 (0.1) | 0.06 (0.06) | 0.04 (0.1) |

*Note.* Standard deviation (SD) appears in parentheses.

*Supplementary Table 27.* Prediction results (Coefficient of Determination [R^2^]) for verbal and non-verbal cognitive functions from FC from the right hemisphere across samples, i.e. total, older and younger.

| FC Right |  | Total | | Older | | Younger | |
| --- | --- | --- | --- | --- | --- | --- | --- |
| Mod. | Tar. | EN | SVR | EN | SVR | EN | SVR |
| DMN | SF | 0.03 (0.03) | 0.02 (0.03) | 0  (0.02) | 0  (0.02) | -0.03 (0.03) | -0.03 (0.05) |
|  | PF | 0.01 (0.03) | 0.01 (0.03) | 0  (0.05) | -0.01 (0.04) | -0.02 (0.04) | -0.01 (0.04) |
|  | VF | 0.03 (0.03) | 0.02 (0.04) | -0.01 (0.04) | 0.01 (0.05) | -0.03 (0.03) | -0.03 (0.04) |
|  | VOC | -0.02 (0.02) | -0.01 (0.03) | -0.02 (0.03) | 0  (0.03) | -0.01 (0.02) | -0.05 (0.04) |
|  | VER | 0.02 (0.03) | 0.02 (0.03) | 0.01 (0.05) | 0.01 (0.12) | -0.03 (0.03) | -0.04 (0.04) |
|  | TMT-A | 0.07 (0.03) | 0.03 (0.05) | -0.02 (0.05) | -0.04 (0.07) | -0.01 (0.06) | -0.04 (0.08) |
|  | vWM | 0.14 (0.05) | 0.13 (0.04) | 0  (0.05) | 0.01 (0.04) | 0.01 (0.04) | 0.01 (0.05) |
| FPN | SF | 0  (0.01) | 0  (0.02) | -0.01 (0.02) | -0.01 (0.03) | -0.02 (0.04) | -0.03 (0.05) |
|  | PF | -0.02 (0.02) | -0.02 (0.04) | -0.02 (0.03) | -0.03 (0.04) | -0.03 (0.03) | -0.04 (0.07) |
|  | VF | 0  (0.03) | 0  (0.03) | -0.02 (0.04) | -0.02 (0.05) | -0.03 (0.03) | -0.03 (0.03) |
|  | VOC | -0.01 (0.02) | -0.02 (0.03) | -0.03 (0.04) | -0.02 (0.06) | -0.02 (0.02) | -0.04 (0.03) |
|  | VER | 0  (0.02) | 0  (0.03) | -0.02 (0.04) | -0.01 (0.04) | -0.02 (0.01) | -0.03 (0.03) |
|  | TMT-A | 0.06 (0.04) | 0.03 (0.06) | -0.01 (0.05) | -0.05 (0.07) | -0.03 (0.04) | -0.07 (0.08) |
|  | vWM | 0.05 (0.03) | 0.04 (0.04) | -0.01 (0.02) | -0.01 (0.02) | -0.03 (0.04) | -0.02 (0.05) |
| FPNDMN | SF | 0  (0.02) | 0.01 (0.02) | -0.02 (0.02) | -0.03 (0.03) | -0.03 (0.05) | -0.04 (0.1) |
|  | PF | -0.01 (0.02) | -0.01 (0.05) | -0.04 (0.03) | -0.05 (0.05) | -0.03 (0.04) | -0.02 (0.05) |
|  | VF | 0  (0.03) | 0  (0.04) | -0.03 (0.03) | -0.03 (0.04) | -0.03 (0.04) | -0.03 (0.07) |
|  | VOC | -0.01 (0.02) | 0  (0.04) | -0.02 (0.04) | -0.02 (0.04) | -0.04 (0.05) | -0.05 (0.06) |
|  | VER | 0.01 (0.03) | 0.01 (0.05) | -0.02 (0.04) | -0.03 (0.06) | -0.04 (0.04) | -0.03 (0.04) |
|  | TMT-A | 0.06 (0.05) | 0.02 (0.06) | -0.03 (0.05) | -0.05 (0.06) | -0.01 (0.03) | -0.06 (0.08) |
|  | vWM | 0.1  (0.04) | 0.08 (0.04) | -0.02 (0.06) | -0.01 (0.05) | -0.01 (0.05) | 0  (0.08) |
| WHOLE | SF | 0.05 (0.02) | -0.03 (0.05) | -0.01 (0.05) | -0.09 (0.1) | -0.06 (0.06) | -0.13 (0.09) |
|  | PF | 0  (0.03) | -0.09 (0.06) | -0.03 (0.05) | -0.07 (0.08) | -0.03 (0.05) | -0.16 (0.1) |
|  | VF | 0.03 (0.04) | -0.04 (0.05) | 0  (0.07) | -0.06 (0.1) | -0.04 (0.06) | -0.16 (0.08) |
|  | VOC | -0.01 (0.02) | -0.15 (0.07) | -0.03 (0.05) | -0.09 (0.07) | -0.02 (0.04) | -0.26 (0.09) |
|  | VER | 0.03 (0.04) | -0.07 (0.06) | 0  (0.07) | -0.03 (0.1) | -0.02 (0.02) | -0.24 (0.08) |
|  | TMT-A | 0.11 (0.04) | 0.04 (0.06) | -0.02 (0.04) | -0.2 (0.1) | -0.03 (0.09) | -0.09 (0.12) |
|  | vWM | 0.17 (0.05) | 0.1 (0.06) | -0.01 (0.04) | -0.19 (0.11) | -0.01 (0.07) | -0.08 (0.11) |

*Note.* Standard deviation (SD) appears in parentheses.

*Supplementary Table 28.* Prediction results (Coefficient of Determination [R^2^]) for verbal and non-verbal cognitive functions from SC from the left hemisphere across samples, i.e. total, older and younger.

| SC Left |  | Total | | Older | | Younger | |
| --- | --- | --- | --- | --- | --- | --- | --- |
| Mod. | Tar. | EN | SVR | EN | SVR | EN | SVR |
| DMN | SF | 0.02  (0.02) | 0.02 (0.02) | -0.02 (0.02) | -0.03 (0.03) | -0.03 (0.04) | -0.03 (0.04) |
|  | PF | 0  (0.01) | -0.01 (0.02) | -0.02 (0.02) | -0.03 (0.03) | -0.02 (0.03) | -0.04 (0.04) |
|  | VF | 0.01  (0.02) | 0.01 (0.02) | -0.02 (0.02) | -0.03 (0.05) | -0.02 (0.03) | -0.04 (0.04) |
|  | VOC | 0  (0.02) | -0.01 (0.03) | -0.02 (0.02) | -0.02 (0.03) | -0.02 (0.03) | -0.03 (0.03) |
|  | VER | 0.02  (0.02) | 0.02 (0.02) | -0.03 (0.03) | -0.03 (0.05) | -0.02 (0.03) | -0.06 (0.04) |
|  | TMT-A | 0.14  (0.05) | 0.11 (0.07) | -0.02 (0.06) | -0.06 (0.08) | 0.02 (0.08) | 0.01 (0.08) |
|  | vWM | 0.16  (0.05) | 0.13 (0.06) | -0.04 (0.04) | -0.03 (0.08) | 0.03 (0.06) | 0.04 (0.06) |
| FPN | SF | 0.02  (0.02) | 0.01 (0.02) | -0.03 (0.03) | -0.02 (0.03) | -0.03 (0.03) | -0.03 (0.05) |
|  | PF | -0.01 (0.02) | -0.01 (0.02) | -0.02 (0.04) | -0.03 (0.05) | -0.02 (0.02) | -0.03 (0.03) |
|  | VF | 0.02  (0.02) | 0.02 (0.02) | -0.03 (0.04) | -0.03 (0.05) | -0.03 (0.03) | -0.05 (0.07) |
|  | VOC | -0.01 (0.02) | -0.01 (0.02) | -0.01 (0.02) | -0.02 (0.02) | -0.02 (0.03) | -0.05 (0.05) |
|  | VER | 0.02  (0.02) | 0.01 (0.02) | -0.03 (0.04) | -0.05 (0.06) | -0.02 (0.02) | -0.03 (0.04) |
|  | TMT-A | 0.13  (0.05) | 0.1 (0.05) | -0.02 (0.06) | -0.06 (0.07) | -0.01 (0.06) | -0.05 (0.08) |
|  | vWM | 0.12  (0.04) | 0.09 (0.05) | -0.01 (0.02) | -0.02 (0.04) | -0.02 (0.04) | -0.02 (0.05) |
| FPNDMN | SF | 0.01  (0.02) | 0.01 (0.02) | -0.02 (0.01) | -0.02 (0.02) | -0.04 (0.05) | -0.05 (0.07) |
|  | PF | 0  (0.02) | -0.01 (0.02) | -0.03 (0.03) | -0.03 (0.04) | -0.02 (0.02) | -0.05 (0.03) |
|  | VF | 0.01  (0.03) | 0.02 (0.02) | -0.03 (0.03) | -0.02 (0.03) | -0.04 (0.06) | -0.05 (0.09) |
|  | VOC | 0  (0.02) | -0.01 (0.02) | -0.02 (0.02) | -0.02 (0.03) | -0.02 (0.02) | -0.05 (0.05) |
|  | VER | 0.01  (0.02) | 0.01 (0.02) | -0.04 (0.04) | -0.03 (0.06) | -0.03 (0.03) | -0.05 (0.07) |
|  | TMT-A | 0.16  (0.05) | 0.12 (0.07) | -0.01 (0.06) | -0.03 (0.07) | 0.06 (0.06) | 0.01 (0.09) |
|  | vWM | 0.15  (0.05) | 0.14 (0.06) | -0.02 (0.05) | -0.01 (0.06) | 0.03 (0.06) | 0.03 (0.09) |
| WHOLE | SF | 0.05  (0.03) | -0.07 (0.09) | -0.02 (0.02) | -0.21 (0.09) | -0.03 (0.04) | -0.08 (0.08) |
|  | PF | 0.01  (0.03) | -0.09 (0.05) | -0.01 (0.04) | -0.04 (0.08) | -0.03 (0.05) | -0.14 (0.1) |
|  | VF | 0.05  (0.03) | -0.06 (0.06) | -0.02 (0.03) | -0.13 (0.09) | -0.04 (0.06) | -0.11 (0.1) |
|  | VOC | -0.01 (0.02) | -0.09 (0.06) | -0.01 (0.04) | -0.1 (0.08) | -0.05 (0.08) | -0.1 (0.09) |
|  | VER | 0.04  (0.03) | -0.06 (0.06) | 0  (0.05) | -0.1 (0.09) | -0.03 (0.06) | -0.13 (0.11) |
|  | TMT-A | 0.23  (0.06) | 0.18 (0.08) | 0.02 (0.07) | 0  (0.09) | 0.03 (0.08) | 0.03 (0.09) |
|  | vWM | 0.23  (0.04) | 0.19 (0.06) | -0.03 (0.05) | -0.12 (0.11) | 0.01 (0.08) | -0.03 (0.13) |

*Note.* Standard deviation (SD) appears in parentheses.

*Supplementary Table 29.* Prediction results (Coefficient of Determination [R^2^]) for verbal and non-verbal cognitive functions from SC from the right hemisphere across samples, i.e. total, older and younger.

| SC Right |  | Total | | Older | | Younger | |
| --- | --- | --- | --- | --- | --- | --- | --- |
| Mod. | Tar. | EN | SVR | EN | SVR | EN | SVR |
| DMN | SF | 0.04  (0.02) | 0.03 (0.02) | -0.02 (0.02) | -0.02 (0.04) | -0.03 (0.04) | -0.04 (0.05) |
|  | PF | -0.01 (0.02) | -0.01 (0.02) | -0.02 (0.02) | -0.04 (0.03) | -0.03 (0.04) | -0.02 (0.04) |
|  | VF | 0.02  (0.02) | 0.02 (0.03) | -0.03 (0.03) | -0.02 (0.03) | -0.03 (0.04) | -0.03 (0.05) |
|  | VOC | 0  (0.02) | -0.02 (0.04) | -0.03 (0.04) | -0.02 (0.05) | -0.02 (0.03) | -0.04 (0.03) |
|  | VER | 0.02  (0.03) | 0.02 (0.02) | -0.03 (0.05) | -0.02 (0.04) | -0.03 (0.05) | -0.02 (0.03) |
|  | TMT-A | 0.11  (0.04) | 0.08 (0.07) | -0.05 (0.06) | -0.06 (0.06) | 0.03 (0.04) | -0.02 (0.08) |
|  | vWM | 0.16  (0.05) | 0.14 (0.05) | -0.02 (0.02) | -0.01 (0.03) | 0.03 (0.04) | 0.01 (0.06) |
| FPN | SF | 0.01  (0.01) | 0.01 (0.01) | -0.01 (0.02) | -0.01 (0.02) | -0.02 (0.04) | -0.04 (0.03) |
|  | PF | -0.01 (0.02) | -0.02 (0.02) | -0.02 (0.02) | -0.03 (0.03) | -0.02 (0.03) | -0.02 (0.03) |
|  | VF | 0  (0.02) | 0  (0.03) | -0.02 (0.02) | -0.02 (0.03) | -0.04 (0.06) | -0.05 (0.07) |
|  | VOC | -0.02 (0.02) | -0.02 (0.03) | -0.03 (0.04) | -0.03 (0.05) | -0.02 (0.03) | -0.03 (0.05) |
|  | VER | -0.01 (0.01) | -0.01 (0.03) | -0.03 (0.04) | -0.02 (0.03) | -0.03 (0.03) | -0.07 (0.15) |
|  | TMT-A | 0.11  (0.05) | 0.08 (0.06) | -0.04 (0.05) | -0.05 (0.06) | 0  (0.06) | -0.03 (0.08) |
|  | vWM | 0.09  (0.05) | 0.09 (0.05) | -0.03 (0.04) | -0.02 (0.04) | 0  (0.04) | 0.01 (0.05) |
| FPNDMN | SF | 0.03  (0.02) | 0.02 (0.02) | -0.02 (0.03) | -0.02 (0.02) | -0.02 (0.02) | -0.04 (0.04) |
|  | PF | 0.01  (0.02) | -0.02 (0.02) | -0.02 (0.02) | -0.05 (0.03) | -0.03 (0.08) | -0.01 (0.05) |
|  | VF | 0.03  (0.02) | 0.01 (0.02) | -0.01 (0.02) | -0.03 (0.03) | -0.03 (0.04) | -0.03 (0.04) |
|  | VOC | 0  (0.01) | -0.02 (0.03) | -0.03 (0.05) | -0.02 (0.04) | -0.03 (0.02) | -0.02 (0.04) |
|  | VER | 0.03  (0.03) | 0.01 (0.03) | -0.03 (0.04) | -0.04 (0.05) | -0.03 (0.03) | -0.03 (0.06) |
|  | TMT-A | 0.16  (0.03) | 0.11 (0.06) | -0.02 (0.06) | -0.05 (0.06) | 0.03 (0.06) | 0.03 (0.07) |
|  | vWM | 0.14  (0.05) | 0.13 (0.07) | -0.04 (0.04) | -0.03 (0.05) | 0.08 (0.06) | 0.07 (0.08) |
| WHOLE | SF | 0.05  (0.03) | -0.03 (0.08) | -0.01 (0.03) | -0.03 (0.07) | -0.02 (0.04) | -0.18 (0.1) |
|  | PF | 0  (0.02) | -0.14 (0.07) | -0.02 (0.03) | -0.09 (0.06) | -0.03 (0.04) | -0.12 (0.08) |
|  | VF | 0.04 (  0.03) | -0.07 (0.07) | -0.01 (0.04) | -0.02 (0.06) | -0.02 (0.02) | -0.16 (0.09) |
|  | VOC | -0.01 (0.04) | -0.1 (0.07) | -0.02 (0.03) | -0.1 (0.08) | -0.05 (0.07) | -0.09 (0.07) |
|  | VER | 0.03  (0.03) | -0.08 (0.08) | 0  (0.04) | -0.02 (0.06) | -0.03 (0.03) | -0.15 (0.07) |
|  | TMT-A | 0.2  (0.06) | 0.17 (0.07) | -0.02 (0.05) | -0.09 (0.1) | 0.03 (0.06) | 0.02 (0.08) |
|  | vWM | 0.21  (0.04) | 0.18 (0.05) | -0.02 (0.02) | -0.11 (0.09) | 0.06 (0.07) | 0.01 (0.11) |

*Note.* Standard deviation (SD) appears in parentheses.

*Supplementary Table 30.* Prediction results (Coefficient of Determination [R^2^]) for verbal and non-verbal cognitive functions from FC across samples, i.e. total, older and younger, controlled for eTIV.

| FC eTIV |  | Total | | Older | | Younger | |
| --- | --- | --- | --- | --- | --- | --- | --- |
| Mod. | Tar. | EN | SVR | EN | SVR | EN | SVR |
| DMN | SF | 0.02 (0.02) | 0  (0.04) | -0.01 (0.02) | -0.03 (0.04) | -0.02 (0.03) | -0.05 (0.05) |
|  | PF | 0.01 (0.02) | -0.01 (0.04) | 0  (0.02) | -0.01 (0.04) | 0  (0.01) | -0.07 (0.04) |
|  | VF | 0.03 (0.02) | 0.02 (0.05) | 0.01 (0.02) | 0.02 (0.05) | 0  (0.01) | -0.08 (0.05) |
|  | VOC | -0.01 (0.02) | 0  (0.05) | 0  (0.04) | 0  (0.06) | -0.01 (0.04) | -0.06 (0.05) |
|  | VER | -0.01 (0.02) | 0.08 (0.04) | 0.02 (0.04) | 0.03 (0.06) | 0  (0.01) | -0.1 (0.05) |
|  | TMT-A | 0.02 (0.03) | -0.01 (0.03) | 0  (0.03) | -0.01 (0.05) | 0  (0.03) | -0.02 (0.05) |
|  | vWM | 0.1  (0.05) | 0  (0.03) | 0.01 (0.05) | 0  (0.05) | 0.02 (0.05) | 0.03 (0.06) |
| FPN | SF | 0.14 (0.05) | 0  (0.03) | 0  (0.02) | 0  (0.03) | -0.01 (0.02) | -0.03 (0.04) |
|  | PF | 0.15 (0.06) | -0.03 (0.04) | 0  (0.02) | -0.01 (0.03) | -0.01 (0.02) | -0.02 (0.05) |
|  | VF | 0.01 (0.02) | 0.01 (0.02) | 0.01 (0.02) | 0  (0.03) | -0.01 (0.02) | -0.03 (0.03) |
|  | VOC | 0  (0.02) | 0.08 (0.05) | 0  (0.03) | 0.01 (0.02) | 0  (0.01) | -0.03 (0.03) |
|  | VER | 0.02 (0.03) | 0.13 (0.05) | 0.01 (0.02) | 0  (0.04) | -0.01 (0.04) | -0.02 (0.03) |
|  | TMT-A | 0  (0.02) | -0.1 (0.05) | 0  (0.06) | -0.01 (0.05) | -0.01 (0.01) | -0.07 (0.03) |
|  | vWM | 0.01 (0.03) | -0.05 (0.05) | 0.02 (0.06) | 0.02 (0.08) | 0  (0.01) | -0.02 (0.02) |
| FPNDMN | SF | 0.1  (0.05) | -0.06 (0.04) | 0  (0.01) | -0.09 (0.09) | -0.01 (0.03) | -0.15 (0.07) |
|  | PF | 0.06 (0.04) | 0  (0.02) | -0.01 (0.02) | -0.09 (0.07) | -0.01 (0.02) | -0.16 (0.09) |
|  | VF | 0.02 (0.02) | -0.04 (0.06) | 0.01 (0.03) | -0.05 (0.07) | 0  (0.01) | -0.17 (0.08) |
|  | VOC | 0.01 (0.02) | 0.1 (0.06) | -0.01 (0.03) | -0.06 (0.06) | -0.01 (0.02) | -0.12 (0.07) |
|  | VER | 0.02 (0.02) | 0.05 (0.04) | 0.01 (0.04) | -0.04 (0.08) | -0.01 (0.03) | -0.22 (0.09) |
|  | TMT-A | 0.01 (0.02) | -0.14 (0.07) | -0.01 (0.06) | -0.08 (0.07) | 0  (0.04) | -0.05 (0.07) |
|  | vWM | 0.02 (0.03) | -0.14 (0.08) | 0.01 (0.04) | 0  (0.08) | 0.03 (0.05) | 0.06 (0.07) |
| WHOLE | SF | 0.01 (0.02) | -0.13 (0.08) | -0.01 (0.03) | -0.08 (0.06) | -0.02 (0.04) | -0.19 (0.1) |
|  | PF | -0.02 (0.04) | -0.07 (0.06) | -0.02 (0.05) | -0.08 (0.07) | 0  (0) | -0.21 (0.1) |
|  | VF | 0.01 (0.03) | -0.14 (0.07) | -0.01 (0.03) | -0.05 (0.06) | 0  (0) | -0.23 (0.09) |
|  | VOC | -0.01 (0.02) | -0.1 (0.08) | -0.02 (0.05) | -0.15 (0.1) | -0.01 (0.04) | -0.18 (0.09) |
|  | VER | 0  (0.03) | 0.1 (0.06) | 0  (0.05) | -0.05 (0.07) | 0  (0) | -0.24 (0.09) |
|  | TMT-A | 0.15 (0.05) | 0.12 (0.06) | -0.02 (0.03) | -0.09 (0.08) | -0.01 (0.04) | -0.01 (0.08) |
|  | vWM | 0.15 (0.06) | 0.1 (0.07) | 0.01 (0.04) | -0.09 (0.08) | -0.01 (0.08) | -0.01 (0.08) |

*Note.* Standard deviation (SD) appears in parentheses.

*Supplementary Table 31.* Prediction results (Coefficient of Determination [R^2^]) for verbal and non-verbal cognitive functions from SC across samples, i.e. total, older and younger, controlled for eTIV.

| SC eTIV |  | Total | | Older | | Younger | |
| --- | --- | --- | --- | --- | --- | --- | --- |
| Mod. | Tar. | EN | SVR | EN | SVR | EN | SVR |
| DMN | SF | 0.03  (0.03) | 0.03 (0.05) | 0  (0.02) | -0.02 (0.05) | 0  (0.02) | -0.01 (0.05) |
|  | PF | 0.01  (0.02) | -0.02 (0.04) | 0  (0.01) | -0.05 (0.04) | -0.02 (0.03) | -0.03 (0.04) |
|  | VF | 0.04  (0.02) | 0.02 (0.04) | 0  (0.02) | -0.03 (0.04) | 0  (0.03) | -0.02 (0.04) |
|  | VOC | 0  (0.01) | 0.03 (0.03) | -0.01 (0.02) | -0.03 (0.05) | -0.01 (0.02) | -0.03 (0.05) |
|  | VER | 0  (0.02) | 0.17 (0.05) | -0.01 (0.03) | -0.01 (0.04) | -0.02 (0.04) | -0.04 (0.04) |
|  | TMT-A | 0.04  (0.02) | 0.02 (0.02) | 0.03 (0.03) | -0.01 (0.05) | 0.03 (0.06) | 0.01 (0.06) |
|  | vWM | 0.19  (0.05) | 0  (0.04) | -0.02 (0.03) | -0.03 (0.07) | 0.03 (0.06) | 0.03 (0.07) |
| FPN | SF | 0.17  (0.04) | 0.03 (0.04) | 0  (0.01) | -0.01 (0.02) | -0.01 (0.02) | -0.01 (0.04) |
|  | PF | 0.19  (0.06) | -0.03 (0.04) | -0.01 (0.02) | -0.02 (0.03) | -0.01 (0.03) | -0.01 (0.03) |
|  | VF | 0.03  (0.01) | 0.02 (0.04) | 0  (0.03) | 0  (0.03) | -0.01 (0.03) | -0.03 (0.09) |
|  | VOC | 0.01  (0.02) | 0.16 (0.04) | -0.01 (0.02) | -0.01 (0.04) | -0.03 (0.08) | -0.02 (0.05) |
|  | VER | 0.03  (0.03) | 0.17 (0.05) | -0.01 (0.03) | 0  (0.03) | -0.02 (0.05) | -0.02 (0.03) |
|  | TMT-A | 0  (0.02) | -0.04 (0.03) | 0.03 (0.03) | 0  (0.05) | 0.02 (0.05) | 0.01 (0.06) |
|  | vWM | 0.02  (0.02) | -0.07 (0.05) | 0  (0.02) | 0  (0.04) | 0.02 (0.05) | 0.01 (0.07) |
| FPNDMN | SF | 0.18  (0.04) | -0.03 (0.04) | -0.02 (0.03) | -0.1 (0.06) | -0.01 (0.02) | -0.06 (0.05) |
|  | PF | 0.13  (0.03) | 0  (0.02) | -0.01 (0.03) | -0.12 (0.07) | -0.04 (0.08) | -0.06 (0.06) |
|  | VF | 0.03  (0.02) | -0.02 (0.04) | -0.02 (0.04) | -0.11 (0.07) | -0.01 (0.02) | -0.06 (0.05) |
|  | VOC | 0.01  (0.02) | 0.19 (0.07) | -0.01 (0.02) | -0.06 (0.06) | -0.03 (0.07) | -0.06 (0.07) |
|  | VER | 0.03  (0.02) | 0.13 (0.04) | -0.01 (0.03) | -0.07 (0.07) | -0.03 (0.07) | -0.07 (0.08) |
|  | TMT-A | 0.01  (0.02) | -0.02 (0.05) | 0.05 (0.03) | 0.01 (0.05) | 0.03 (0.09) | 0.03 (0.09) |
|  | vWM | 0.02  (0.03) | 0  (0.05) | -0.01 (0.02) | -0.03 (0.06) | 0.05 (0.06) | 0.03 (0.1) |
| WHOLE | SF | 0.02  (0.03) | 0.01 (0.05) | -0.01 (0.02) | -0.07 (0.06) | -0.01 (0.03) | -0.06 (0.06) |
|  | PF | 0.02  (0.02) | -0.05 (0.05) | 0  (0.04) | 0.01 (0.05) | -0.02 (0.06) | -0.04 (0.07) |
|  | VF | 0.04  (0.03) | -0.05 (0.04) | 0.01 (0.03) | 0  (0.06) | -0.01 (0.03) | -0.07 (0.05) |
|  | VOC | 0  (0.02) | 0.01 (0.04) | 0.01 (0.03) | -0.04 (0.04) | -0.02 (0.05) | -0.08 (0.05) |
|  | VER | 0.04  (0.03) | 0.22 (0.06) | 0.01 (0.03) | 0.01 (0.05) | -0.01 (0.04) | -0.06 (0.07) |
|  | TMT-A | 0.23  (0.05) | 0.15 (0.07) | 0.01 (0.04) | 0  (0.08) | 0.05 (0.08) | 0.06 (0.1) |
|  | vWM | 0.19  (0.06) | 0.18 (0.06) | 0  (0.02) | -0.06 (0.07) | 0.01 (0.07) | 0.01 (0.1) |

*Note.* Standard deviation (SD) appears in parentheses.

References

Abraham, A., Pedregosa, F., Eickenberg, M., Gervais, P., Mueller, A., Kossaifi, J., Gramfort, A., Thirion, B., & Varoquaux, G. (2014). Machine learning for neuroimaging with scikit-learn. *Frontiers in Neuroinformatics*, *8*. https://doi.org/10.3389/fninf.2014.00014

Fanton, S., & Thompson, W. H. (2023). *NetPlotBrain*: A Python package for visualizing networks and brains. *Network Neuroscience*, *7*(2), 461–477. https://doi.org/10.1162/netn_a_00313

Schaefer, A., Kong, R., Gordon, E. M., Laumann, T. O., Zuo, X.-N., Holmes, A. J., Eickhoff, S. B., & Yeo, B. T. T. (2018). Local-Global Parcellation of the Human Cerebral Cortex from Intrinsic Functional Connectivity MRI. *Cerebral Cortex*, *28*(9), 3095–3114. https://doi.org/10.1093/cercor/bhx179
